# Targeting Regnase-1 in B7-H3-CAR T cells reprograms the tumor microenvironment and enhances antitumor efficacy for osteosarcoma

**DOI:** 10.1016/j.xcrm.2026.103008

**Published:** 2026-08-25

**Authors:** Adeleye O. Adeshakin, Hao Shi, S. Scott Perry, Heather Sheppard, Phuong Nguyen, Xiang Sun, Peipei Zhou, Jean-Yves Métais, Trevor Cunningham, Anil KC, Liqing Tian, Vivek Peche, Mollie S. Prater, Deanna M. Langfitt, Carla O'Reilly, Jeoungeun J. Park, Ashley Chabot, Sheng Zhou, Azusa Matsubara, GaHyun Lee, Shengdar Q. Tsai, Shondra M. Pruett-Miller, Lindsay J. Talbot, Jason T. Yustein, Giedre Krenciute, Christopher DeRenzo, Hongbo Chi, Stephen Gottschalk

**Affiliations:** 1 Department of Bone Marrow Transplantation and Cellular Therapy, St. Jude Children’s Research Hospital, Memphis, TN 38105, USA; 2 Department of Immunology, St. Jude Children’s Research Hospital, Memphis, TN 38105, USA; 3 Department of Pathology, St. Jude Children’s Research Hospital, Memphis, TN 38105, USA; 4 Flow Cytometry and Cell Sorting Shared Resource, St. Jude Children’s Research Hospital, Memphis, TN 38105, USA; 5 Center for Advanced Genome Engineering, St. Jude Children’s Research Hospital, Memphis, TN 38105, USA; 6 Experimental Cellular Therapeutics Laboratory, St. Jude Children’s Research Hospital, Memphis, TN 38105, USA; 7 Department of Surgery, St. Jude Children’s Research Hospital, Memphis, TN 38105, USA; 8 Department of Hematology, St. Jude Children’s Research Hospital, Memphis, TN 38105, USA; 9 Aflac Cancer and Blood Disorders Center and the Winship Cancer Institute, Emory University, Atlanta, GA 30322, USA; 10 Center of Excellence for Pediatric Immuno-Oncology, St. Jude Children’s Research Hospital, Memphis, TN 38105, USA

**Keywords:** B7-H3, Regnase-1, CAR, T cell therapy, tumor microenvironment, osteosarcoma, gene editing, immunotherapy

## Abstract

The microenvironment in solid tumors represents an immunosuppressive therapeutic barrier to CAR T cell therapy, and it is currently unknown whether it can be reshaped by the deletion of negative regulators in CAR T cells. To address this knowledge gap, we evaluated the intrinsic and extrinsic effects of deleting the negative regulator Regnase-1 (Reg-1) in B7-H3-CAR T cells for the immunotherapy of osteosarcoma. Reg-1 knockout (KO) improved the antitumor activity of human and murine B7-H3-CAR T cells *in vivo*. In immune-competent models, Reg-1 KO also endowed murine B7-H3-CAR T cells with the ability to create a proinflammatory landscape characterized by an influx of interferon gamma (IFN-γ)-producing endogenous T cells and natural killer (NK) cells and a reduction of inhibitory myeloid cells, including M2-like macrophages. Thus, deleting Reg-1 has cell- and non-cell-autonomous benefits, nominating Reg-1 KO B7-H3-CAR T cells as a promising cell product for early-phase clinical testing in patients with solid tumors.

## Highlights

• Regnase-1 deletion has cell-autonomous and non-cell-autonomous effects
• Regnase-1 KO enhances the effector function of B7-H3-CAR T cells
• Regnase-1 KO B7-H3-CAR T cells create a pro-inflammatory tumor microenvironment
• Human Regnase-1 KO B7-H3-CAR T cells demonstrate strong translational potential

## Graphical abstract

**Figure:**
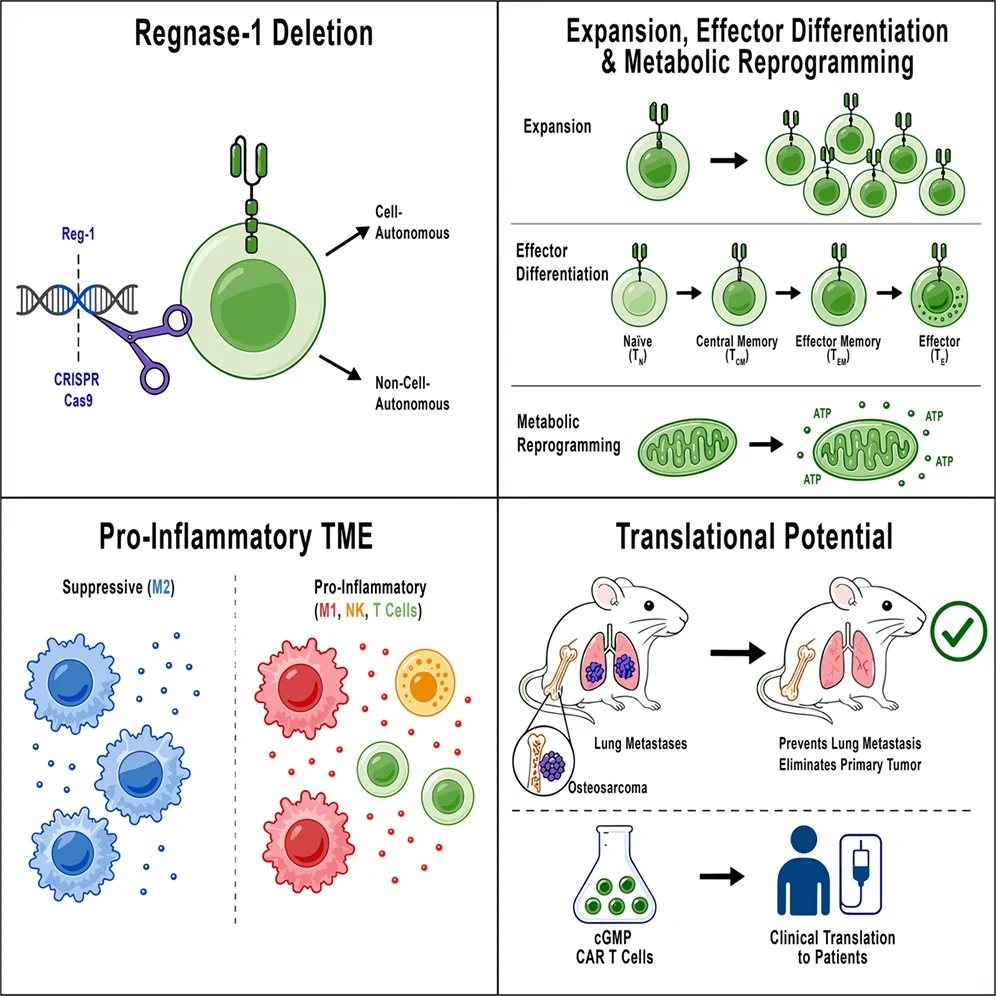

## Teaser

Adeshakin et al. demonstrated that deletion of Regnase-1 in B7-H3-CAR T cells enhanced their antitumor activity and endowed them with the capability to induce a pro-inflammatory tumor microenvironment. These findings nominate Regnase-1 knockout B7-H3-CAR T cells as a promising cell product for clinical testing in patients with solid tumors.

## Introduction

Adoptive immunotherapy with chimeric antigen receptor (CAR)-expressing T cells has been successful for CD19- or BCMA-positive hematological malignancies, leading to their Food and Drug Administration (FDA) approval.^1^ However, CAR T cell therapy has been less effective in early-phase clinical studies for solid tumors.^2^^,^^3^^,^^4^^,^^5^^,^^6^^,^^7^^,^^8^^,^^9^ Second genetic modification of CAR T cells has evolved as a promising approach to augment their function, including expression of immunostimulatory molecules or the deletion of negative regulators.^3^^,^^10^^,^^11^^,^^12^^,^^13^^,^^14^^,^^15^^,^^16^^,^^17^^,^^18^^,^^19^^,^^20^

Among established negative regulators, Regnase-1 (Reg-1), a ribonuclease that regulates immune responses,^21^^,^^22^^,^^23^ has emerged as a promising candidate. We and other investigators have demonstrated that, upon Reg-1 knockout (KO), tumor-specific CD19-CAR T or T cell receptor (TCR)-transgenic CD8^+^ T cells persist longer, express more effector molecules, and have increased oxidative phosphorylation (OXPHOS) compared to unmodified T cells.^10^^,^^11^^,^^24^^,^^25^ Mechanistic studies revealed that the basic leucine zipper ATF-like transcription factor (BATF) is the key molecule that mediates Reg-1 KO-mediated reprogramming of CD8^+^ T cells.^10^

While these studies highlight the benefit of Reg-1 KO on improving CAR T cell function, little is known about the efficacy of Reg-1 KO CAR T cells in immune-competent models for solid tumors, with only one study demonstrating the benefit of Reg-1 KO in murine CD19-CAR T cells.^11^ In addition, the effect of Reg-1 KO CAR T cells on endogenous immune cells, a critical barrier of therapeutic efficacy,^26^ has not been examined in immune-competent tumor models.

Here, we evaluated the effects of Reg-1 KO on the cell-autonomous and non-cell-autonomous functions of murine CAR T cells targeting B7-H3 (CD276), an antigen expressed on a broad range of solid tumors,^27^^,^^28^^,^^29^^,^^30^^,^^31^ in immune-competent osteosarcoma models and the cell-autonomous effects of Reg-1 KO in human B7-H3-CAR T cells. We show that human and murine Reg-1 KO B7-H3-CAR T cells have improved effector function *in vivo* and that murine Reg-1 KO B7-H3-CAR T cells create an immunostimulatory lung microenvironment. Thus, deleting Reg-1 has cell-autonomous and non-cell-autonomous effects, not only improving the effector function of CAR T cells but also enabling them to create a proinflammatory microenvironment.

## Results

### Reg-1 KO improves the antitumor activity of B7-H3-CAR T cells

**Figure 1:**
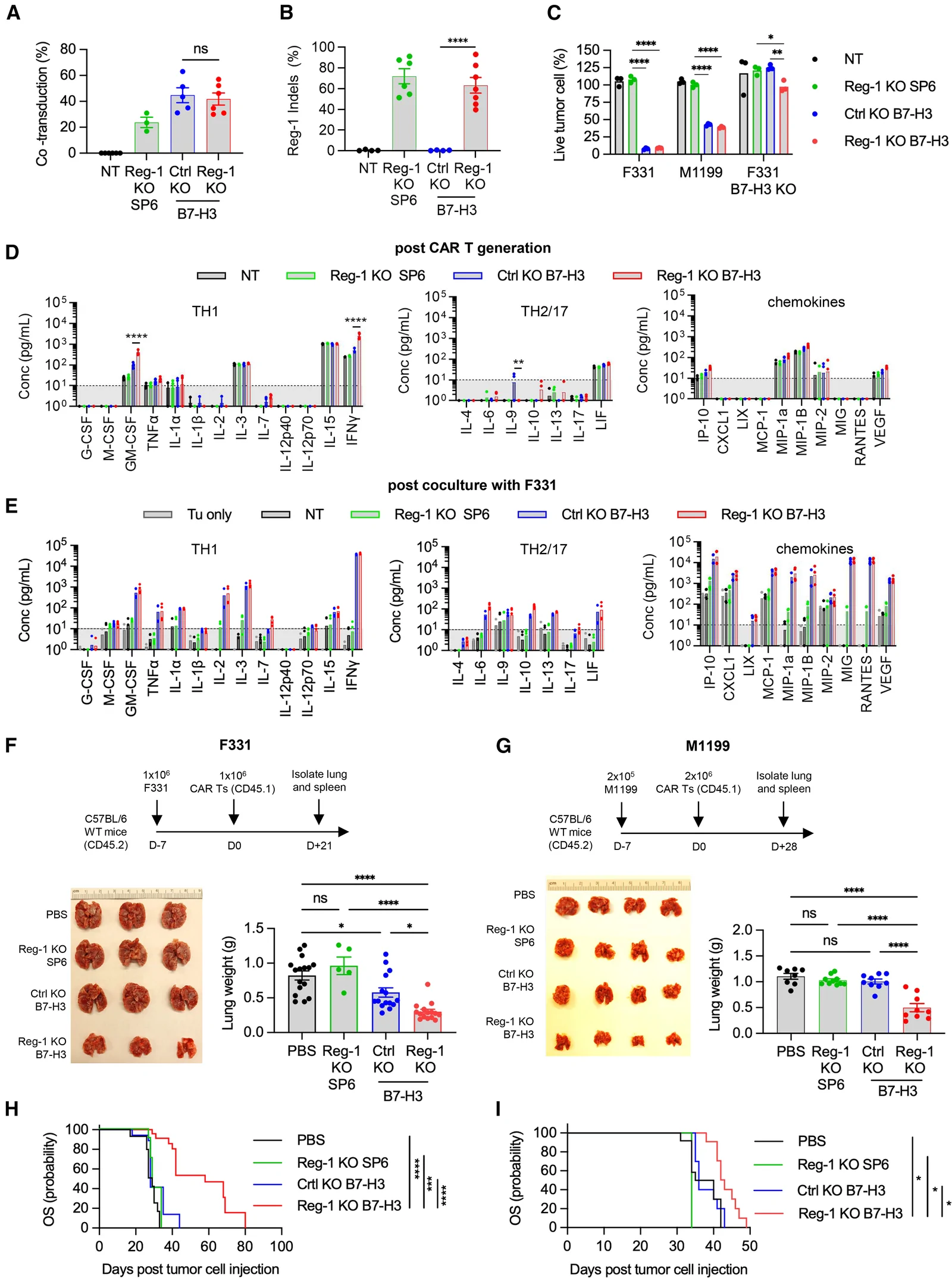
Reg-1 KO improves the antitumor activity of B7-H3-CAR T cells (A) Co-transduction efficiency of indicated sgRNA (ametrine) and CAR in NT, Ctrl KO B7-H3-CAR, Reg-1 KO SP6-CAR, or Reg-1 KO B7-H3-CAR T cells. *n* = 3–6 (one-way ANOVA with Tukey’s test for multiple comparisons). (B) Insertion and deletion (Indel) analysis of Reg-1. *n* = 4–7 (one-way ANOVA with Tukey’s test for multiple comparisons). (C) Cytotoxicity assay with B7-H3-positive (F331, M1199) and B7-H3-negative (B7-H3 KO F331) targets at an E:T ratio of 2:1. *n* = 3 (two-way ANOVA with Tukey’s test for multiple comparisons). (D and E) Multiplex analysis of cytokines and chemokines post (D) CAR T cell production (two-way ANOVA with Tukey’s test for multiple comparisons) and (E) 48 h after stimulation with F331 cells at 1:1 E:T ratio. Tumor cell and NT T cell groups, *n* = 3; all other conditions, *n* = 5. (F–I) C57BL/6 mice (CD45.2^+^) were injected with 1 × 10^6^ F331 or 2 × 10^5^ M1199 OS cells i.v. and received a single i.v. dose of indicated CAR T cells (Cas9^+^CD45.1^+^) or PBS on day 7. (F and G) Experimental scheme (upper), representative macroscopic images of lungs (lower left), and lungs weight (lower right) on (F) day 21 for F331 or (G) day 28 for M1199 (*n* = 5–15 per group for lungs weight analysis). Data are represented as mean ± SEM. (H and I) Kaplan-Meier survival curve, OS: overall survival. (H) F331, log rank Mantel-Cox test, *n* = 8 for Reg-1 KO SP6-CAR T cells; all other groups, *n* = 13. (I) M1199, log rank Mantel-Cox test, *n* = 5 for Reg-1 KO SP6-CAR T cells; all other groups, *n* = 10; two-way ANOVA with Tukey’s test for multiple comparisons. ∗*p* < 0.05, ∗∗*p* < 0.01, ∗∗∗*p* < 0.001, ∗∗∗∗*p* < 0.0001.

To establish the therapeutic effects of targeting Reg-1 for CAR T cell therapy, we generated Reg-1 and control (Ctrl) KO B7-H3-CAR T cells and Reg-1 KO CAR T cells targeting an irrelevant antigen (SP6). Naive CD8^+^ T cells from Cas9-transgenic mice (CD45.1^+^) were co-transduced with retroviral vectors encoding the respective CAR or single-guide (sg) RNA and an ametrine reporter. Median double transduction efficacy was 42% (Figures 1A and S1A), and Reg-1 gene editing efficiency ranged from 40% to 93% (Figures 1B, S1B, and S1C). To evaluate the specificity of B7-H3-CAR T cells, we used murine B7-H3-positive (F331, M1199) or B7-H3-negative (F331 B7-H3-KO) osteosarcoma cell lines (Figures S2A–S2C). Reg-1 and Ctrl KO B7-H3-CAR T cells killed F331 and M1199 cell lines, in contrast to non-transduced (NT) or Reg-1 KO SP6-CAR T cells (Figures 1C and S3). Likewise, Ctrl or Reg-1 KO B7-H3-CAR T cells showed no or minimal killing of F331 B7-H3 KO cells, verifying antigen specificity (Figures 1C and S3). To examine the effects of Reg-1 KO on cytokine/chemokine production, we analyzed CAR T cells post-generation (Figure 1D) and after coculture with F331 cells (Figure 1E). Post-generation, Reg-1 KO B7-H3-CAR T cells produced significantly more GM-CSF and interferon gamma (IFN-γ) than Ctrl KO B7-H3-CAR T cells (Figure 1D). After coculture with F331, Reg-1 and Ctrl KO B7-H3-CAR T cells produced more TH1 (granulocyte-macrophage colony-stimulating factor [GM-CSF], IFN-γ, tumor necrosis factor alpha [TNF-α], interleukin [IL]-1α, IL-2, IL-3, and IL-15), and TH2 (IL-6, IL-10, IL-13, and leukemia inhibitory factor [LIF]) cytokines compared to Reg-1 KO SP6-CAR T cells (Figure 1E; Table S1). However, there were no significant differences between Ctrl and Reg-1 KO B7-H3 CAR T cells, except that Reg-1 KO B7-H3-CAR T cells produced higher levels of IL-7 compared to Ctrl KO B7-H3-CAR T cells (Figure 1E; Table S1). The concentration of several chemokines (IP10, LIX, monocyte chemoattractant protein-1 [MCP-1], macrophage inflammatory protein-1α [MIP-1α], MIP-1β, MIG, and RANTES) was also increased after F331/Reg-1 and Ctrl KO B7-H3-CAR T cell co-cultures (Figure 1E; Table S1). Since osteosarcoma cell line can increase their endogenous chemokine production in response to TH1 cytokines, further studies are needed to determine their source.^32^

To evaluate the effects of Reg-1 KO on the antitumor activity of B7-H3-CAR T cells *in vivo*, we focused on models for osteosarcoma tumor colonization in the lung, which is the primary site of metastasis.^33^^,^^34^^,^^35^ F331 or M1199 lung-tumor-bearing congenic mice (CD45.2^+^) received a single intravenous (i.v.) injection of Reg-1 KO B7-H3-CAR, Ctrl KO B7-H3-CAR, Reg-1 KO SP6-CAR T cells, or PBS only (Figures 1F and 1G). Infusion of Reg-1 KO B7-H3-CAR T cells had no overt side effects in mice (Figure S4). Reg-1 KO B7-H3-CAR T cells had superior antitumor activity compared to all other experimental groups, as revealed by lung weight comparison, and visual and histopathological observations, on day 21 (F331) or day 28 (M1199) post-infusion (Figures 1F, 1G, and S5). This resulted in a significant survival advantage of mice treated with Reg-1 KO B7-H3-CAR T cells (Figures 1H and 1I). Immunohistochemistry (IHC) analysis revealed that F331 and M1199 tumors continued to express B7-H3 compared to F331 B7-H3 KO tumors (Figure S6A). Moreover, after Reg-1 KO B7-H3-CAR T cell therapy, progressive tumors continued to express B7-H3, excluding antigen loss as a mechanism of immune escape (Figure S6B). We also observed improved antitumor activity of Reg-1 KO B7-H3-CAR T cells in a subcutaneous (s.c.) F331 model (Figures S7A–S7C) and when Reg-1 KO B7-H3-CAR T cells were prepared from CD4- and CD8-selected T cells in the i.v. F331 model (Figures S8A–S8F). Reg-1 KO B7-H3 CAR T cells also had enhanced antitumor activity in the B7-H3-positive Lewis lung carcinoma (LLC) model, indicating that the benefit of Reg-1 KO is not restricted to B7-H3-positive osteosarcoma (Figures S9A–S9E).

### Reg-1 KO enhances B7-H3-CAR T cell expansion

**Figure 2:**
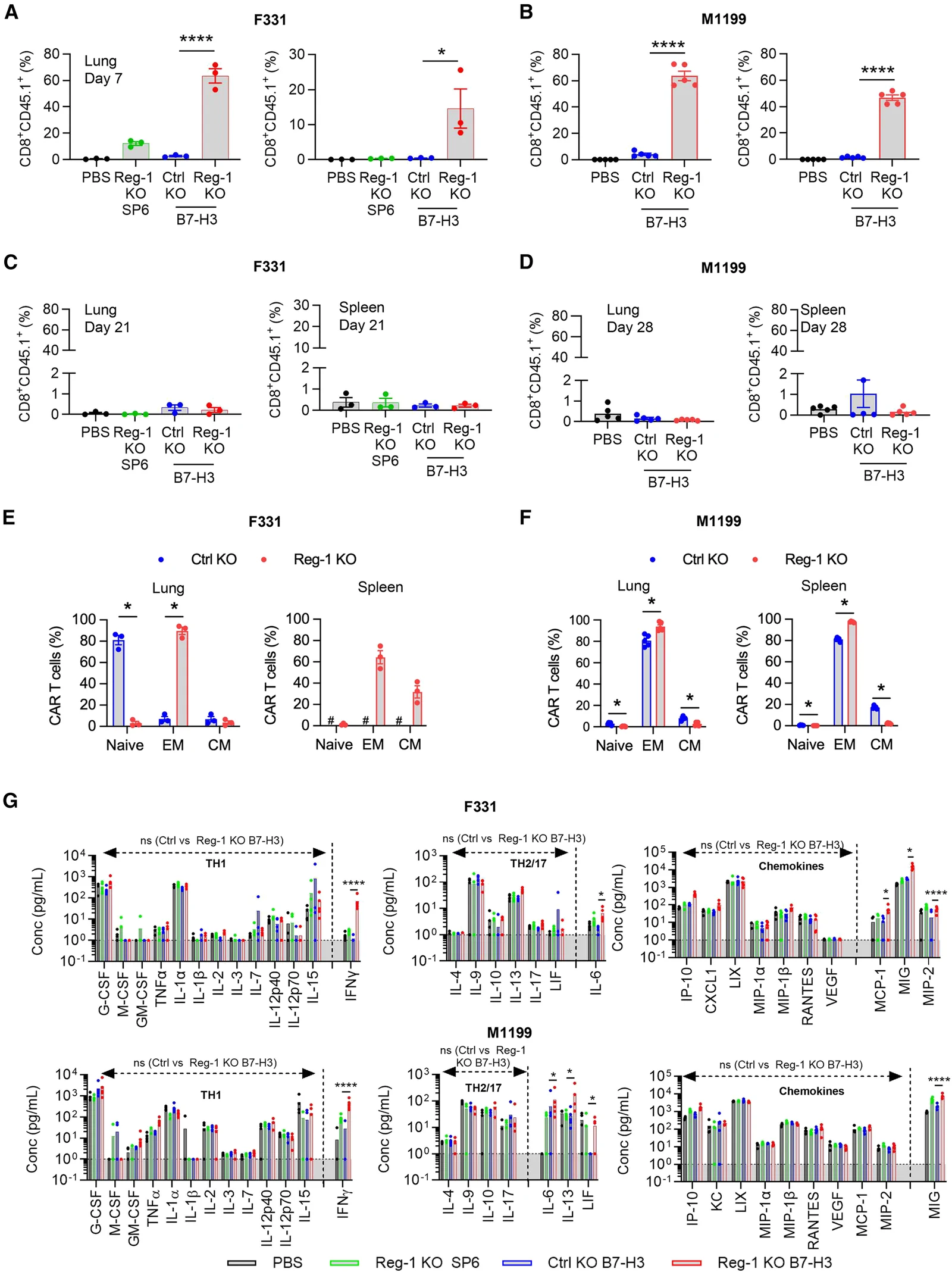
Reg-1 KO enhances B7-H3-CAR T cell expansion C57BL/6 mice were injected with 1 × 10^6^ F331 or 2 × 10^5^ M1199 OS cells (i.v.) and received a single i.v. dose of indicated CAR T cell population or PBS on day 7. (A–D) Quantification of lung- and spleen-infiltrating CD8^+^CD45.1^+^ T cells on day 7 for (A) F331 or (B) M1199, (C) on day 21 for F331, and (D) on day 28 for M1119 post-T-cell injection (F331: *n* = 3, M1199: *n* = 5; one-way ANOVA with Tukey’s test for multiple comparisons. (E and F) Phenotyping of CAR T cells: naive (CD62L^+^CD44^–^), effector memory (EM; CD62L^–^CD44^+^), and central memory (CM; CD62L^+^CD44^+^) in (E) F331 tumor-bearing mice (#: not done) and (F) M1199 tumor-bearing mice on day 7 post-CAR T cell infusion (F331; *n* = 3, M1199: *n* = 5; unpaired Student’s *t* test). (G) Multiplex analysis of cytokines in serum on day 7 post-CAR T cell infusion or PBS in the F331 (top) and M1199 (bottom) model; *n* = 5; mean ± SEM. Two-way ANOVA with Tukey’s test for multiple comparisons; ∗*p* < 0.05; ∗∗*p* < 0.01; ∗∗∗*p* < 0.001; ∗∗∗∗*p* < 0.0001.

To gain insight into the functional state of Reg-1 KO B7-H3-CAR, Ctrl KO B7-H3-CAR, or Reg-1 KO SP6-CAR T cells and their effects on endogenous immune cells, we euthanized F331 tumor-bearing mice on day 7 or day 21 or M1199 tumor-bearing mice on day 7 or day 28 post-CAR T cell infusion to perform phenotypic analysis of immune cells from lungs and spleens. In addition, blood samples were collected for complete blood counts (CBCs) and for cytokine/chemokine analysis post-CAR T cell infusion. Flow cytometric analysis of lungs and spleens revealed a significantly higher frequency of Reg-1 KO B7-H3-CAR T cells compared to Ctrl KO B7-H3-CAR or Reg-1 KO SP6-CAR T cells on day 7 in F331 and M119 tumor-bearing mice (Figures 2A and 2B), which declined to baseline levels by day 21/28 (Figures 2C and 2D). An increased frequency of Reg-1 KO B7-H3-CAR T cells in tumors was confirmed by ISH (*in situ* hybridization) (Figure S10). On day 7, phenotypic analysis of Reg-1 KO B7-H3-CAR versus Ctrl KO B7-H3-CAR T cells revealed enhanced differentiation to an effector memory (EM; CD62L^−^CD44^+^) phenotype, with more pronounced effects observed in the F331 model (Figures 2E and 2F); no differences in programmed cell death protein 1 (PD-1)-expression were observed between Ctrl and Reg-1 KO B7-H3-CAR T cells (Figures S11A and S11B).

Expansion of Reg-1 KO B7-H3-CAR T cells coincided with significant splenomegaly on day 7, which resolved by day 21/28 (Figures S12A and S12B), and was mirrored by transient lymphopenia and monocytopenia (only F331 model) (Figures S12C and S12D). Lymphopenia was not specific to Reg-1 KO B7-H3-CAR T cells since it also was observed post-Reg-1 KO SP6-CAR T cells. In both models, Reg-1 KO B7-H3-CAR T cells induced higher serum levels of IFN-γ, IL-6, and MIG compared to Ctrl KO B7-H3-CAR T cells on day 7 post-infusion. In addition, model-specific increases of cytokines (IL-13 and LIF) or chemokines (MCP-1 and MIP-2) were observed (Figure 2G). Reg-1KO B7-H3 CAR T cells also demonstrated greater expansion and effector differentiation, accompanied by splenomegaly, lymphopenia, and increased serum IFN-γ concentrations, compared to Ctrl KO B7-H3 CAR T cells in the LLC model (Figures S13A–S13F).

Reg-1 KO B7-H3-CAR T cells caused distinct changes in the composition of the lung immune cell infiltrates, including a higher percentage of natural killer (NK) cells and a lower percentage of B cells (in M1199 model) (Figures S14A and S14B). Changes in the myeloid compartment (neutrophils: CD11b^+^Ly6C^+^Ly6G^+^; monocytes: CD11b^+^Ly6C^+^Ly6G^−^; M1-like macrophages: CD11b^+^F4/80^+^CD206^−^; M2-like macrophages: CD11b^+^F4/80^+^CD206^+^; and dendritic cells [DCs]: CD11b^+^CD11c^+^) were model-dependent. For instance, lungs from F331 mice had increased neutrophils and reduced M2-like macrophages following infusion of Reg-1 KO B7-H3-CAR T cells compared to Ctrl KO B7-H3-CAR T cells, while in the M1199 model no difference in neutrophils but increased M2-like macrophages were observed (Figures S14A and S14B). Among endogenous T cell populations, we did not observe consistent changes between the F331 and M1199 models regarding the percentages of CD3^+^, CD4^+^, and CD8^+^ T cells (Figures S14A and S14B) or various CD4^+^ T cell subsets (naive, EM, and central memory [CM]) (Figures S15A and S15B). Within the endogenous CD8^+^ T cells, Reg-1 KO B7-H3-CAR T cells increased EM and decreased naive subsets in both the lungs and spleens from F331 mice (Figure S15A), whereas no significant differences were observed in the M1199 model (Figure S15B). Finally, Reg-1 KO B7-H3-CAR T cells induced expression of PD-1- and programmed death-ligand 1 (PD-L1) in endogenous T cells and myeloid cells (Figures S15A and S15B). Changes in the endogenous immune cell composition were also observed in the LLC model, including a higher percentage of NK and CD4^+^ EM T cells and increased expression of PD-1 on CD8^+^ T cells and PD-L1 on monocytes and DCs (Figures S16A and S16B).

In summary, Reg-1 KO induced antigen-dependent expansion and effector differentiation of B7-H3-CAR T cells and created a systemic and pulmonary-specific proinflammatory environment. While the increased percentage of myeloid cells expressing PD-L1 raised the possibility of reprogramming of the myeloid compartment to an inhibitory state, combining Reg-1 KO B7-H3-CAR T cells with anti-PD-L1 treatment did not further improve their antitumor activity (Figures S17A and S17B).

### Reg-1 KO enhances proinflammatory and metabolic signaling pathways in B7-H3-CAR T cells

**Figure 3:**
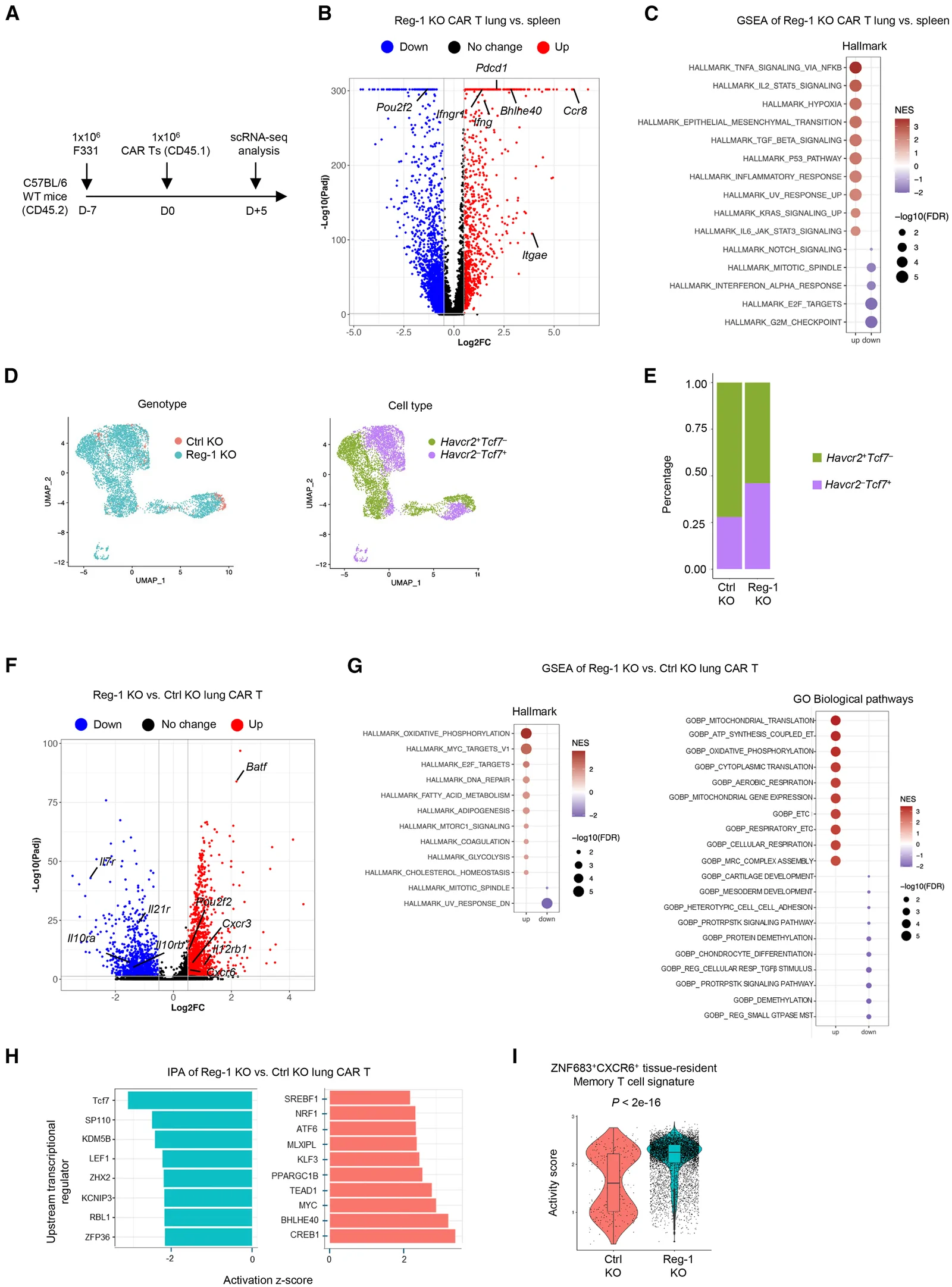
Reg-1 KO enhances pro-inflammatory and metabolic signaling pathways during B7-H3 CAR T cell expansion phase (A) Experimental scheme: C57BL/6 mice received a single dose of 5 × 10^6^ Reg-1 KO or Ctrl KO B7-H3-CAR T cells (Cas9^+^CD45.1^+^) on day 7 post-F331 tumor cell injection. Lungs and spleens were isolated on day 5 post-infusion, and cells from three mice per condition were pooled for scRNA-seq analysis. (B) Volcano plot showing differentially expressed genes (Log_2_FC > 0.5 or < −0.5 and adjusted *p* < 0.05) in Reg-1 KO B7-H3-CAR T cells from lungs vs. spleens. (C) Top 10 significantly (FDR<0.05) upregulated and top 5 downregulated pathways in Reg-1 KO B7-H3-CAR T cells from lungs vs. spleens (GSEA using Hallmark gene sets). (D) UMAP of Reg-1 KO or Ctrl KO B7-H3 CAR T cells (left) and *Havcr2* or *Tcf7* expression (right) in lungs. (E) Proportion of *Havcr2* and *Tcf7* in Reg-1 KO and Ctrl KO B7-H3-CAR T cells in lungs. (F) Upregulated (red) or downregulated (blue) genes (log_2_FC > 0.5 and adjusted *p* < 0.05) in Reg-1 KO compared to Ctrl KO B7-H3-CAR T cells. (G) Top 10 significantly (FDR<0.05) upregulated and top two downregulated metabolism-related pathways (Hallmark gene sets; left) or top 10 upregulated and downregulated GO biological pathways (right) in Reg-1 KO compared to Ctrl KO B7-H3-CAR T cells (GSEA). (H) Ingenuity pathway analysis of transcriptional regulators of Reg-1 KO compared to Ctrl KO B7-H3-CAR T cells in lungs. (I) Activity score of a CD8^+^ ZNF683^+^CXCR6^+^ tissue-resident memory T cell signature in Ctrl KO and Reg-1 KO B7-H3-CAR T cells in lungs. ET, electron transport; ETC, electron transport chain; MRC, mitochondrial respiratory chain; PROTRPSTK, positive regulation of transmembrane receptor protein serine threonine kinase; REG, regulation; RESP, respiration; MST, mediated signal transduction.

Next, we compared the transcriptomics profiles between Ctrl KO and Reg-1 KO B7-H3-CAR T cells by performing single-cell RNA sequencing (scRNA-seq) of CAR T cells isolated from the lungs and spleens of F331 tumor-bearing mice on day 5 post-CAR T cell infusion (Figure 3A) when sufficient numbers of both CAR T cell populations were present for analysis. Uniform manifold approximation and projection (UMAP) analysis revealed distinct clustering of Ctrl KO and Reg-1 KO B7-H3-CAR T cells in lungs and spleens and an increased number of Reg-1 KO relative to Ctrl KO B7-H3-CAR T cells in both tissues (Figures S18A–S18E). In addition, single-cell TCR sequencing (scTCR-seq) analysis revealed that CAR T cells were more clonally expanded compared to non-CAR T cells (Figures S18F and S18G). Reg-1 KO B7-H3-CAR T cells in the lungs expressed higher levels of effector genes, including *Itgae* (encoding CD103), *Ccr8*, *Bhlhe40*, *Pdcd1*, *Ifng*, and *Ifngr1*, and gene set enrichment analysis (GSEA) revealed significant enrichment of Hallmark gene signatures associated with TNF-α signaling, IL-2-STAT5 signaling, and inflammatory responses, as compared to Reg-1 KO B7-H3-CAR T cells from the spleens (Figures 3B and 3C).

We next compared the cellular differentiation states (based on *Havcr2* and *Tcf7* expression) and gene expression profiles between Ctrl KO and Reg-1 KO B7-H3-CAR T cells collected from the lung. Compared to Ctrl KO B7-H3-CAR T cells, Reg-1 KO B7-H3-CAR T cells had a higher percentage of *Havcr2*^*−*^*Tcf7*^+^ and a lower percentage of *Havcr2*^*+*^*Tcf7*^*−*^ cells (Figures 3D and 3E). Differential gene expression analysis revealed that Reg-1 KO CAR T cells expressed higher levels of *Batf*, a transcription factor critical for effector differentiation of CD8^+^ T cells.^10^^,^^36^^,^^37^ Cytokine receptor gene expression was either increased (*Il12rb1*) or decreased (*Il10ra*, *Il10rb*, *Il7r*, and *Il21r*) in Reg-1 KO relative to Ctrl KO B7-H3-CAR T cells (Figure 3F), highlighting that cytokine receptor expression is unlikely to predict T cell functionality as observed in other models.^13^^,^^38^ Reg-1 KO CAR T cells expressed higher levels of chemokine receptors (*Cxcr3* and *Cxcr6*) that orchestrate tumor-reactive T cell infiltration and function.^39^^,^^40^ Based on GSEA, Reg-1 KO CAR T cells upregulated cell proliferation signatures and metabolism-related signatures compared to Ctrl KO CAR T cells (Figure 3G). In addition, ingenuity pathway analysis (IPA) revealed that Reg-1 KO CAR T cells upregulated activities for Bhlhe40, Myc, and PGC-1 (PPARGC1B), which are important for T cell mitochondrial function^41^ (Figure 3H). Finally, Reg-1 KO B7H3-CAR T cells upregulated a pan-cancer ZNF683^+^CXCR6^+^ tissue-resident memory CD8^+^ T cell signature^42^ (Figure 3I), suggesting increased tissue residency features in the absence of Reg-1. We repeated the analysis using matched cell numbers for Ctrl and Reg-1 KO B7-H3-CAR T cells and confirmed all key findings, eliminating group size differences as a potential confounder (Figures S19A–S19F).

### Reg-1 KO B7-H3-CAR T cells reduce inhibitory myeloid cells and increase the frequency of IFN-γ-producing T cells

**Figure 4:**
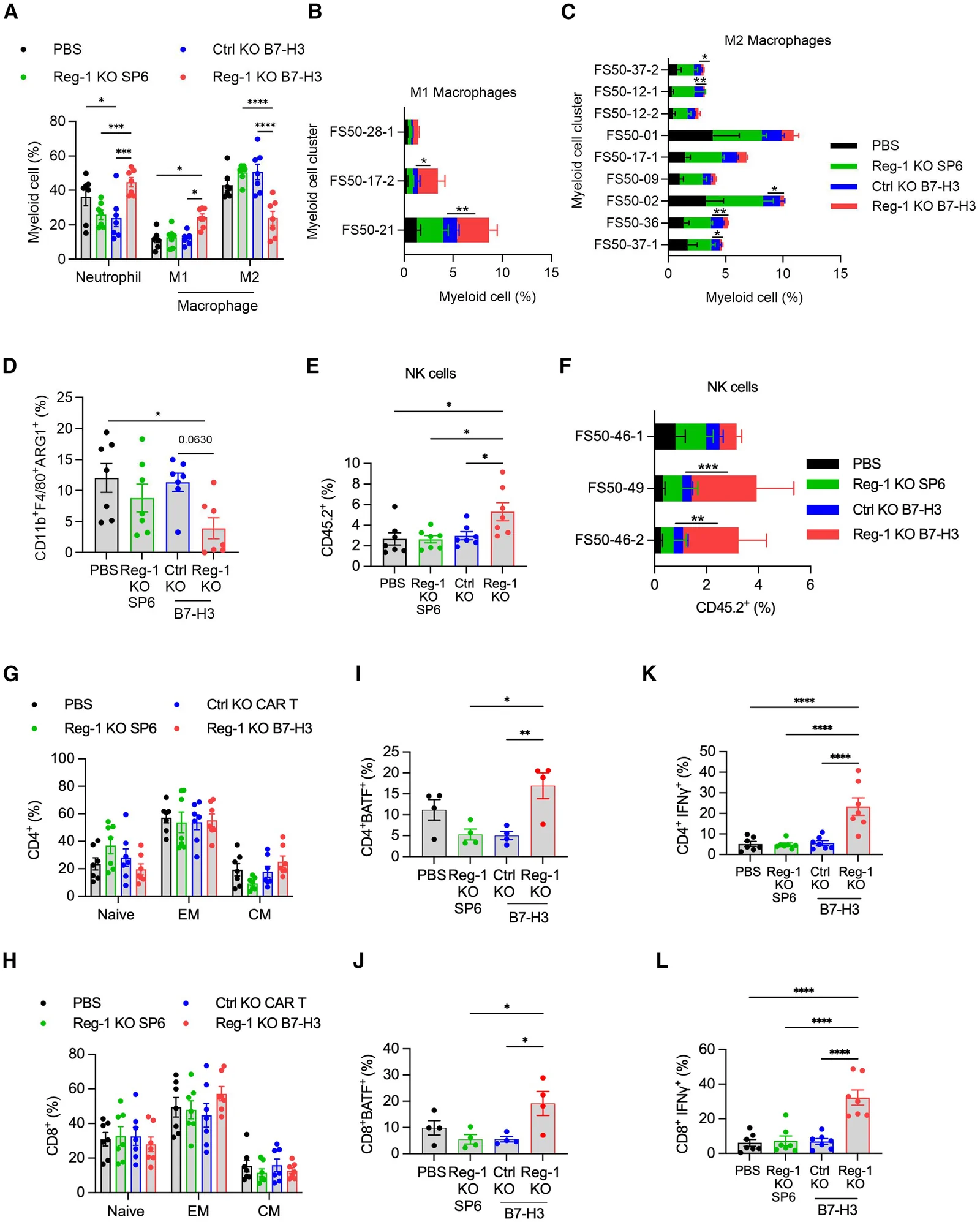
Reg-1 KO B7-H3-CAR T cells reduce endogenous inhibitory myeloid cells and increase pro-inflammatory immune cells Cell surface marker expression data were pooled from two high-parameter immunophenotyping experiments described for Figures S20, S21, and S22, totaling seven lung samples for each of the four treatment conditions. Separate aliquots of lungs cell suspensions were also probed with an intracellular flow cytometry panel (Table S6). (A) Percentages of myeloid cell subsets (see Tables S3 and S5 for definition). (B and C) (B) Three M1- and (C) nine M2-like macrophage clusters were identified based on Table S3; *p* < 0.05 is shown for Ctrl KO versus Reg-1 KO B7-H3-CAR T cell comparison. (D) Frequencies of ARG1-expressing CD11b^+^F4/80^+^ cells. (E and F) (E) Percentages and (F) identified clusters of NK cells based on Table S3. In (F), *p* < 0.05 is shown for Ctrl KO vs. Reg-1 KO B7-H3-CAR T cell comparison. (G–L) Percentages of (G and H) naive, EM, and CM populations; (I and J) BATF-expressing cells; and (K and L) IFN-γ-expressing cells among endogenous CD4^+^ T cells (G, I, and K) and CD8^+^ T cells (H, J, and L); *n* = 7 (G, H, K, and L) and *n* = 4 (I and J); mean ± SEM. Two-way ANOVA with Tukey’s test for multiple comparisons; ∗*p* < 0.05; ∗∗*p* < 0.01; ∗∗∗*p* < 0.001; ∗∗∗∗*p* < 0.0001. EM, effector memory; CM, central memory. For (B, C, and F), *n* = 3.

Since our flow cytometric analyses on day 7 demonstrated that Reg-1 KO B7-H3-CAR T cells had significant effects on endogenous immune cells, we next determined whether these effects persisted and performed a flow cytometric analysis of single-cell suspensions from lungs on day 21 post-CAR T cell infusion in the F331 model. In two experiments, we profiled 19 unique cell markers (23 total fluorochromes; Figures S20A–S20D; Tables S2 and S3) and 34 cell unique markers (38 total fluorochromes; Figures S21 and S22A–S22C; Tables S4 and S5). For all experimental groups, the two largest clusters consisted of myeloid and B cells, with T and NK cells being the next most abundant populations (Figures S20 and S22C). CAR T cells were only detected after Reg-1 KO B7-H3-CAR T cell infusion at low levels and co-expressed PD-1 and PD-L1 (Figures S23A and S23B). Within the myeloid compartment, there were significantly higher frequencies of neutrophils and M1-like macrophages, mirrored by a decrease in M2-like macrophages, upon Reg-1 KO B7-H3-CAR T cell therapy (Figures 4A–4C). The MFI of B7-H3 expression on the cell surface of the remaining M2-like macrophages was lower post-Reg-1 KO B7-H3-CAR T cell therapy compared to all other treatment groups, indicative of on target activity of Reg-1 KO B7-H3-CAR T cells (Figures S24A and S24B).

We next analyzed the expression of additional cell surface markers, intracellular cytokines, and transcription factors (Table S6). Reg-1 KO B7-H3-CAR T cells induced a decrease in the frequency of CD11b^+^F4/80^+^ macrophages that expressed arginase 1 (ARG1) (Figure 4D), a hallmark of M2-like macrophages that promote T cell dysfunction in the tumor microenvironment (TME).^43^ This finding was validated by IHC of lung tumors (Figure S25). There was also an increased frequency of NK cells (Figures 4E and 4F). While no significant changes in the frequencies of endogenous naive, EM, and CM T cells were observed between treatment groups (Figures 4G, 4H, S26A, and S26B), there were significantly higher percentages of BATF-positive CD4^+^ and CD8^+^ T cells (Figures 4I and 4J) and IFN-γ-producing CD4^+^ and CD8^+^ T cells (Figures 4K and 4L) post-Reg-1 KO B7-H3-CAR T cell infusion. No significant differences in other cytokines (IL-10, IL-6, TNF-α, and IL-17A) or transcription factors (RORγt, TCF1, and Foxp3) were observed (Figures S26C and S26D; data for TCF1 and Foxp3 are not shown).

### Reg-1 KO B7-H3-CAR T cells induce proinflammatory transcriptional changes in endogenous immune cells

**Figure 5:**
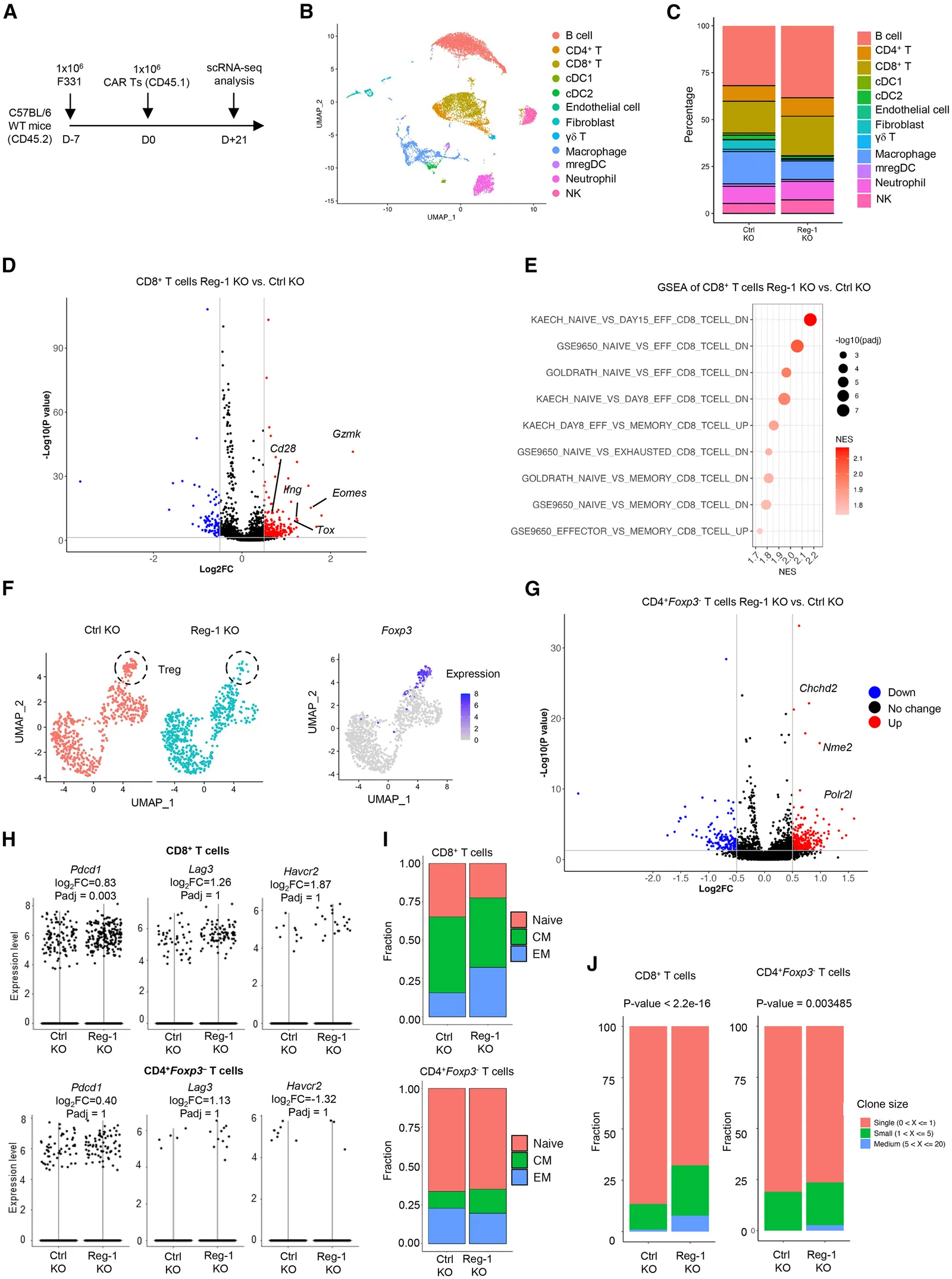
Reg-1 KO B7-H3-CAR T cells increase the effector function of endogenous T and NK cells (A) Experimental scheme: C57BL/6 mice (CD45.2^+^) received a single dose of 1 × 10^6^ Reg-1 KO or Ctrl KO B7-H3-CAR T cells (Cas9^+^CD45.1^+^) on day 7 post-F331 tumor cell injection. Lungs from two mice per treatment groups were isolated on day 21 post-CAR T cell infusion and processed for scRNA-seq analysis. (B) UMAP indicating clusters of immune cell subsets of CD45^+^ cells, fibroblasts, and endothelial cells from Reg-1 KO and Ctrl KO B7-H3-CAR T cell-treated mice. (C) Percentage of immune cells among CD45^+^ cells on day 21 post-CAR T cell infusion. (D) Volcano plot showing upregulated genes in endogenous *Cd8*^+^ T cells of Reg-1 KO vs. Ctrl KO B7-H3-CAR T cell-treated mice. (E) Top nine significantly (false discovery rate [FDR] <0.05) upregulated *C*D8^+^ T cell-related pathways in endogenous CD8^+^ T cells from Reg-1 KO vs. Ctrl KO B7-H3-CAR T cell-treated mice. (F) UMAP projection (left) and *Foxp3* expression (right) in endogenous CD4^+^ T cells in Reg-1 KO and Ctrl KO B7-H3-CAR T cell-treated mice. (G) Volcano plot illustrating upregulated genes in endogenous *Foxp3*-negative *CD4*^+^ T cells from Reg-1 KO vs. Ctrl KO B7-H3-CAR T cell-treated mice. (H) Expression of *Pdcd1*, *Lag3*, and *Havcr2* in CD8^+^ (top) and CD4^+^ (bottom) T cells Reg-1 KO vs. Ctrl KO B7-H3-CAR T cell-treated mice. (I) Fraction of indicated endogenous CD8^+^ (top) and CD4^+^*Foxp3*^*–*^ (bottom) T cell subsets from Ctrl KO and Reg-1 KO B7-H3-CAR T cell-treated mice. (J) Clonal expansion of endogenous CD8^+^ (*p* < 2.2 × 10^−16^) and CD4^+^Foxp3^–^ (*p* = 0.003485) T cells in Reg-1 KO and Ctrl KO B7-H3-CAR T cell-treated mice.

To unbiasedly profile endogenous immune cells, we performed scRNA-seq analysis of the F331 tumor model on day 21 post-Ctrl KO or Reg-1 KO B7-H3-CAR T cell infusion (Figure 5A). Based on published datasets,^44^ this analysis identified 10 endogenous immune cell populations, as well as endothelial cells and fibroblasts (Figures 5B and S27). While all 12 populations were present post-Reg-1 KO or Ctrl KO B7-H3-CAR T cell infusion, their composition differed. Specifically, there were decreased frequencies of macrophages and fibroblasts and increased percentages of CD8^+^ T, NK, and B cells post-Reg-1 KO B7-H3-CAR T cell infusion (Figure 5C).

Using published datasets as a reference,^45^ transcriptional profiling of endogenous *Cd8*^+^ T cells revealed enhanced activation and effector differentiation induced by Reg-1 KO B7-H3-CAR T cells (Figures S28A–S28D). This included increased expression of (1) effector molecules (*Gzmk* and *Ifng*), (2) costimulatory receptors (*Cd28*), and (3) transcription factors associated with T cell differentiation and/or exhaustion (*Tox* and *Eomes*) (Figure 5D). GSEA revealed enrichments of gene signatures associated with T cell effector differentiation (Figure 5E). UMAP analysis of *Cd4*^+^ T cells revealed that Reg-1 KO B7-H3-CAR T cell-treated mice had a lower frequency of endogenous *Foxp3*^+^ T cells (Figure 5F), while *Cd4*^+^*Foxp3*^–^ T cells highly upregulated key genes in metabolic (*Chchd2*), transcriptional (*Nme2*), and mRNA synthesis (*Polr2l*) pathways (Figure 5G). While endogenous *Cd8*^+^ and *Cd4*^+^ T cells post-Reg-1 KO B7-H3-CAR T cell infusion upregulated *Pdcd1* (PD-1) expression, they did not show altered expression of *Lag3* or *Havcr2* (Figure 5H). Furthermore, Reg-1 KO B7-H3-CAR T cells increased the frequency of endogenous effector CD8^+^ T cells and reduced percentages of naive and memory CD8^+^ T cell populations (Figure 5I). Finally, we observed increased clonal expansion of endogenous *Cd4*^+^*Foxp3*^–^ and *Cd8*^+^ T cells post-Reg-1 KO B7-H3-CAR T cell therapy (Figure 5J).

### Reg-1 KO B7-H3-CAR T cells suppress tumor-promoting signature genes of macrophages

Since our scRNA-seq analysis revealed that Reg-1 KO B7-H3-CAR T cells reduced macrophages and fibroblasts, we next interrogated whether they also induced qualitative changes. Subclustering analysis identified four distinct clusters of fibroblasts (0–3), all of which were decreased following Reg-1 KO CAR T cell infusion (Figures S29A–S29C). Since Reg-1 KO B7-H3-CAR T cell reduction of fibroblasts was so profound, detailed GSEA and IPA analysis only yielded no significant differences when matched cell numbers were analyzed.

**Figure 6:**
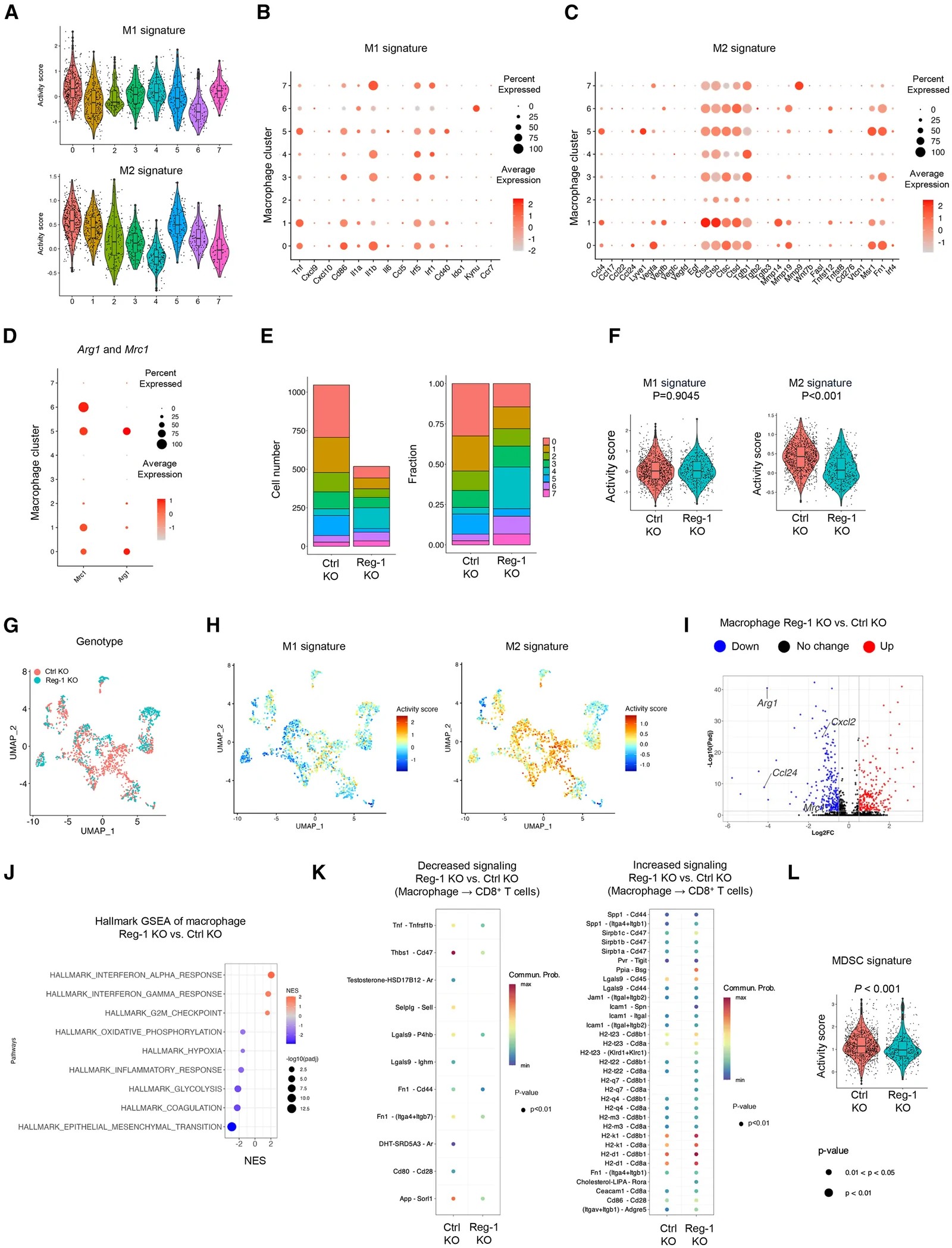
Reg-1 KO B7-H3-CAR T cells suppress tumor-promoting macrophages Macrophage analysis was conducted on day 21 post-CAR T cell infusion (see Figure 5A for experimental scheme). (A) M1-like and M2-like macrophage-associated gene signature activity score of macrophage subclusters. (B–D) Dot plots indicating expression levels of (B) M1-like or (C) M2-like macrophage-associated genes and (D) *Arg1* and *Mrc1*. (E) Cell number (left) and fraction (right) of macrophage clusters post-Reg-1 KO or Ctrl KO B7-H3-CAR T cell infusion. (F) Activity scores of M1-like and M2-like macrophage-associated gene signatures in macrophages. (G and H) UMAP projection showing the distribution of macrophages post-Reg-1 KO or Ctrl KO B7-H3-CAR T cell infusion according to (G) genotype and (H) M1-like and M2-like associated gene signature activity score. (I) Upregulated and downregulated genes (Log_2_FC > 0.5 and adjusted *p* < 0.05) in macrophages. (J) Top three significantly (FDR <0.05) upregulated and top six downregulated pathways in total macrophages post-Reg-1 KO vs. Ctrl KO B7-H3-CAR T cell infusion (GSEA, Hallmark gene sets). (K) Decreased and increased signaling pathways identified from the interaction of macrophages and CD8^+^ T cells by CellChat ligand-receptor interaction prediction. (L) MDSC signature in macrophages showing a significant difference post-Reg-1 KO or Ctrl KO B7-H3-CAR T cell infusion (*p* < 0.001).

We next performed subclustering analysis of macrophages and identified eight distinct clusters (0–7) and generated a heatmap highlighting subcluster-specific marker genes (Figure S30). We next quantified M1- and M2-associated gene signature activity, based on a published dataset,^46^ to define functional differences among the identified macrophage populations (Figure 6A). Subclusters 0 (*Vcan*^*+*^*Thbs1*^*+*^), 4 (*Ace*^*+*^*Pglyrp1*^*+*^), and 7 (*Cxcr2*^*+*^*Mmp9*^*+*^) showed strong enrichment of M1 gene signature activity, whereas subclusters 0 (*Vcan*^*+*^*Thbs1*^*+*^), 1 (*Syngr1*^*+*^*Ms4a7*^*+*^), and 5 (*Folr2*^*+*^*Lyve1*^*+*^) exhibited strong M2 gene signature activity. Additional M1- and M2-associated genes are visualized in dot plots (Figures 6B–6D), highlighting that subclusters 0, 4, and 7 express high levels of *Il1b,* whereas subclusters 0, 1, and 5 express *Mrc1*, *Arg1*, *Ccl4*, and *Msr1*, respectively. Treatment with Reg-1 KO B7-H3 CAR T cells induced an overall reduction in macrophage numbers and decreased the frequency of subclusters enriched for M2-associated gene signatures (clusters 1 and 5), while increasing subclusters enriched for M1-associated gene signatures (clusters 4 and 7) (Figure 6E). This resulted in a global reduction of the M2-associated gene signature in macrophages (Figures 6F–6H). Furthermore, expressions of *Arg1* and *Mrc1*, as well as monocyte-recruiting chemokines (*Ccl24* and *Cxcl2*), were reduced in macrophages after Reg-1 KO B7-H3-CAR T cell therapy (Figure 6I). GSEA also revealed upregulated Hallmark IFN-α/γ response and cell proliferation pathways (Figure 6J), and CellChat analysis^47^ revealed decreased immunosuppressive Thbs1-CD47 interaction and increased probability of major histocompatibility complex (MHC) class I pathway interactions between macrophages and CD8^+^ T cells (Figure 6K). Likewise, gene expression signature analysis revealed a decrease in myeloid-derived suppressor cell (MDSC) signature activity score^48^ in macrophages (Figure 6L).

### Human Reg-1 KO B7-H3-CAR T cells have potent antitumor activity

**Figure 7:**
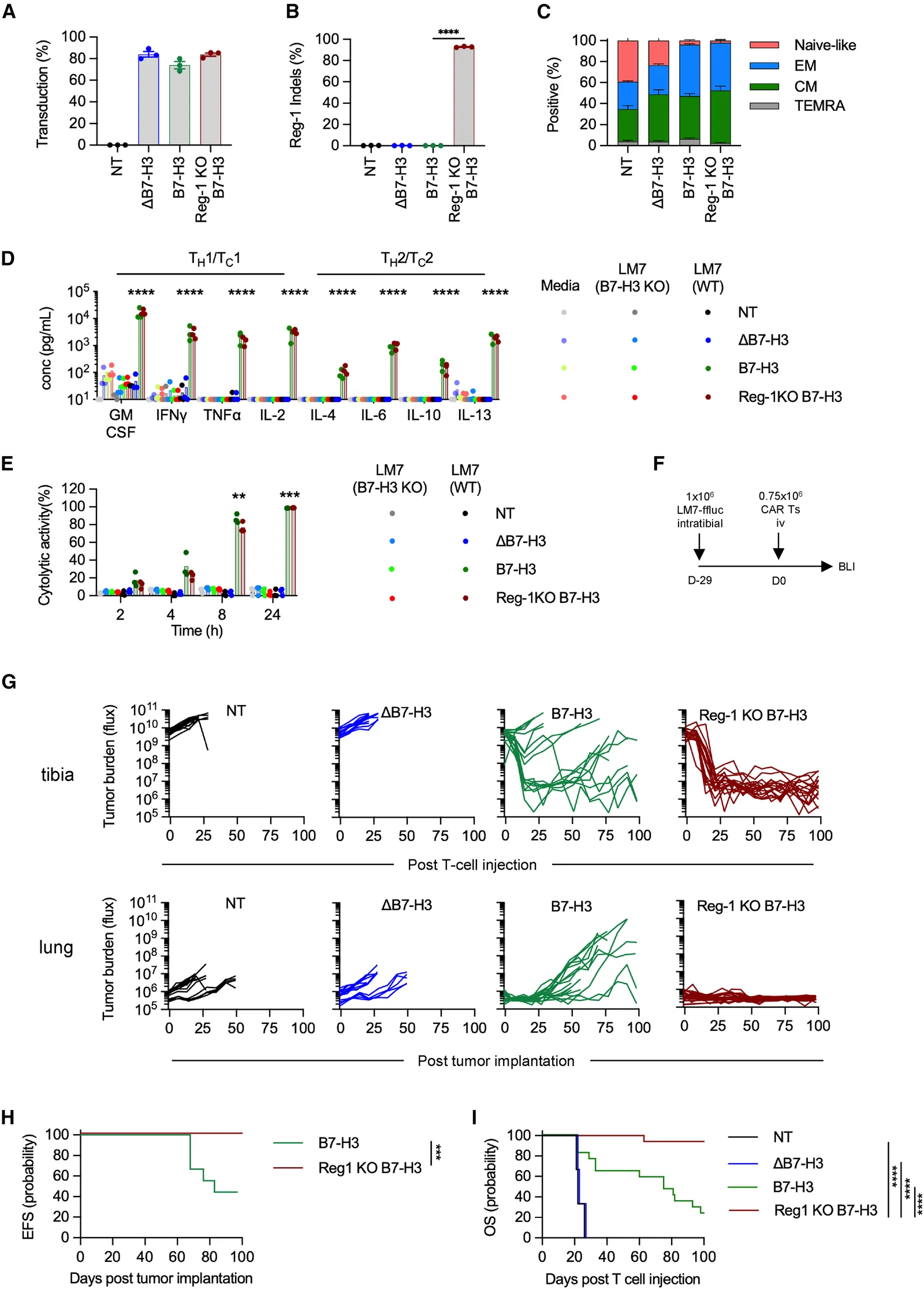
Human Reg-1 KO B7-H3-CAR T cells have improved antitumor activity in OS model (A) Transduction efficiency of CAR T cell populations; *n* = 3. (B) Indel analysis of Reg-1 gene; *n* = 3 (one-way ANOVA with Tukey’s test for multiple comparisons). (C) Immunophenotyping of CAR T cells: naive-like (CCR7+CD45RO−), CM (CCR7+CD45RO+), EM (CCR7−CD45RO+), and terminally differentiated effector memory cells re-expressing CD45RA (TEMRA, CCR7−CD45RO−); *n* = 3; mean ± SEM. (D) Coculture assay with B7-H3-positive (LM7) and B7-H3-negative (B7-H3 KO LM7) tumor cells. Media were collected, and cytokine production was evaluated by ELLA. *p* values are shown for media vs. LM7 comparison for B7-H3 and Reg-1 KO B7-H3-CAR T cells (two-way ANOVA with Tukey’s test for multiple comparisons). (E) Cytotoxicity assay with B7-H3-positive (LM7) and B7-H3-negative (B7-H3 KO LM7) targets at an E:T ratio of 1:1; *n* = 3; mean ± SEM. Two-way ANOVA with Tukey’s test for multiple comparisons. (F–I) NSG mice were implanted intratibially with 1 × 10^6^ LM7-ffluc cells and received a single i.v. dose of the indicated CAR T cell populations or NT T cells 29 days later. (F) Experimental scheme. (G) Quantified Flux values of left tibia and lung fields; *n* = 15–18 mice per group (3 donors and 3 independent experiments). (H) Development of lung metastasis (lung fields Flux: >10^9^) in mice with no evidence of tibial tumors post-CAR T cell infusion; B7-H3 CAR T cells (*n* = 9) and Reg-1 KO B7-H3-CAR T cells (*n* = 18). Kaplan-Meier curve; EFS, event free survival. (I) Kaplan-Meier survival curve; OS, overall survival; log rank Mantel-Cox test; two-way ANOVA with Tukey’s test for multiple comparisons. ∗∗*p* < 0.01, ∗∗∗*p* < 0.001, ∗∗∗∗*p* < 0.0001.

Based on the presented preclinical studies, we adapted our current good manufacturing process (cGMP) to generate human Reg-1 KO B7-H3-CAR T cells for a future first-in-human clinical study. Reg-1 KO B7-H3-CAR T cell products were generated from leukapheresis products using a lentiviral vector encoding a human B7-H3-CAR with a CD28.ζ signaling domain and Cas9:Reg-1 sgRNA ribonucleoproteins (RNPs). The effector function of Reg-1 KO B7-H3-CAR T cell products were compared to NT T cells, non-edited B7-H3-CAR T cells, and B7-H3-CAR T cells expressing a non-functional B7-H3-CAR (ΔB7-H3-CAR). Transduction efficiency ranged from 68.5% to 89.2% (Figure 7A), and Reg-1 gene editing efficiency was confirmed by indel analysis (Figure 7B). Reg-1 KO did not affect the T cell subset distribution (Figures 7C and S31A) or TIM3 and PD-1 expression (Figure S31B). In coculture assays with tumor cells, Reg-1 KO B7-H3-CAR T cells retained their antigen specificity as judged by TH1 and TH2 cytokine production and cytolytic activity (Figures 7D and 7E).

To compare the antitumor activity of the generated B7-H3-CAR T cell products, we utilized our orthotopic LM7 OS model.^49^ Firefly luciferase (ffluc) expressing LM7 cells (LM7-ffluc) were implanted into the tibia of mice, and one month later mice received a single, low dose (0.75 × 10^6^) of NT T cells, ΔB7-H3-CAR, B7-H3-CAR, or Reg-1 KO B7-H3-CAR T cells (Figure 7F). Only Reg-1 KO B7-H3-CAR T cells induced regression of orthoptic tumors consistently and prevented the development of lung metastases, resulting in a striking survival benefit of mice in comparison to all other treatment groups (Figures 7G–7I, S32A, and S32B). In a follow-up experiment, we generated AAVS1 (control, Ctrl) and Reg-1 KO B7-H3-CAR T cells (Figures S33A–S33D) and demonstrated that the Reg-1 KO guide did not have off-target effects (Figure S33E). We confirmed that Reg-1 KO did not alter antigen specificity of B7-H3-CAR T cells as judged by cytotoxicity assays and cytokine production (Figures S33F and S33G) and improved antitumor activity *in vivo* (Figures S34A–S34D). Long-term surviving mice rejected a 2^nd^ dose of tumor cells on day 118 after CAR T cell injection, indicating their long-term functional persistence (Figures S34B and S34E).

## Discussion

Here, we demonstrate that Reg-1 KO has cell-autonomous and non-cell-autonomous effects on B7-H3-CAR T cells, improving not only their antitumor activity in immune competent and xenograft models but also endowing B7-H3-CAR T cells with the ability to create a proinflammatory environment.

Deleting Reg-1 in B7-H3-CAR T cells enhanced their expansion within 7 days post-infusion in lungs and spleens, followed by their contraction within ∼3 weeks post-infusion. This was associated with transient lymphopenia and splenomegaly, and animal-model-dependent monocytopenia. While CAR T cell expansion and splenomegaly were dependent on B7-H3-CAR expression, transient lympho- and monocytopenia also occurred in mice that received Reg-1 KO SP6-CAR T cells. Mice treated with Reg-1 KO B7-H3-CAR T cells exhibited significantly higher serum IFN-γ levels on day 7 post-infusion across all three models and model-dependent changes in other cytokines and chemokines. While IFN-γ is a hallmark cytokine of immune-effector-cell-associated inflammatory syndromes,^50^^,^^51^^,^^52^^,^^53^^,^^54^ Reg-1 KO B7-H3-CAR T cell-treated mice did not develop clinical signs of distress or lost weight.

Transcriptional profiling of Reg-1 KO B7-H3-CAR T cells on day 5 post-infusion was consistent with our previous finding for CD19-CAR and TCR-transgenic T cells.^10^^,^^11^ In addition, we found increased expression of *Ifng* and *Pou2f2*, consistent with recent findings in Reg-1 KO NK cells.^23^ Finally, Reg-1 KO induced a recently described CXCR6-positive CD8^+^ T cell expression signature that counterbalances PD-1-mediated immune suppression.^42^^,^^55^ Post-expansion, CAR T cells, regardless of Reg-1 expression, contracted to low levels by day 21, and a significant number expressed PD-1 and PD-L1, suggestive of signaling in *cis* as described for antigen-presenting cells.^56^ The observed contraction of Reg-1 KO B7-H3-CAR T cells was greater than we previously reported for Reg-1 KO CD19-CAR T cells in immune-competent leukemia animal models,^11^ suggesting a tumor type/TME-specific component.

Our endogenous immune cell analysis of PBS-treated tumors was consistent with the immune cell profile of osteosarcoma lung metastasis, demonstrating a high percentage of myeloid cells, including M2-like macrophages.^57^ Reg-1 KO B7-H3-CAR T cells had significant effects on endogenous immune cells, including an influx of CD8^+^ T cells and NK cells in tumor-bearing lungs on day 7, and a decrease in M2-like macrophages, coinciding with an increase in M1-like macrophages on day 21 post-infusion. This reversal of the immunosuppressive environment might explain why PD-L1 blockade did not further improve their antitumor activity in the F331 model. Limited benefit of combining checkpoint blockade with CAR T cells has also been reported in preclinical and early-phase clinical studies.^58^^,^^59^

Across all three models, Reg-1 KO B7-H3-CAR T cells induced changes in T cell subset composition, altered PD-1 expression on CD4^+^ and/or CD8^+^ T cells, and modulated PD-L1 expression on myeloid cell subsets. However, specific immune cell composition changes were model-dependent and may reflect differences in the underlying genetic alterations of each tumor model. The evaluated osteosarcoma models harbor defined genetic alterations in *Trp53* and *Myc*,^60^ whereas the LLC model possesses multiple genetic alterations, including mutations in *Kras*, *Nras*, and *Trp53*.^61^ These alterations are not only oncogenic but also shape the TME.^62^^,^^63^

Reg-1 KO in CAR T cells significantly improved their antitumor efficacy in xenograft and immune-competent animal models. The benefit of Reg-1 KO was more pronounced in xenograft models in which Reg-1 KO CAR T cells prevented the development of lung metastasis and endowed them with the ability to reject a 2^nd^ tumor challenge ∼4 months after the initial CAR T cell infusion. In immune competent models, tumors eventually progressed. One explanation of limited antitumor activity might be that we did not use lymphodepleting chemotherapy in our study. While it is routinely used prior to CAR T cell infusion in immune competent animal models in the clinic,^64^^,^^65^^,^^66^^,^^67^ it was not used here, because it has direct antitumor effects and transiently depletes endogenous immune cells. Other investigators have demonstrated that deleting Roquin-1 or BCOR further enhances the effector function of Reg-1 KO CAR T cells.^24^^,^^25^ The benefit of combining these or other genetic modifications should be tested for Reg-1 KO B7-H3-CAR T cells in the future.

The antigen-binding domain property of CARs, including affinity and kinetic parameters, influence CAR T cell effector function.^68^^,^^69^^,^^70^ Thus, the used B7-H3 antigen recognition domain may affect B7-H3-CAR T cell dynamics and efficacy.^68^^,^^71^ The anti-mouse (m276) and anti-human (MGA271) single-chain variable fragments (scFvs) used in our CARs were selected based on their validated antigen specificity and functional activity in prior studies.^27^^,^^72^ Likewise, MGA271-based CAR T cells are currently being evaluated in early-phase clinical trials.^27^^,^^73^^,^^74^^,^^75^ However, the effects of Reg-1 KO should be interpreted in the context of the specific CARs we used, and future studies are needed to assess the generalizability of our findings across other B7-H3-CARs.^68^^,^^76^^,^^77^

In conclusion, we demonstrate that deleting the negative regulator Reg-1 improved the effector function of B7-H3-CAR T cells and endowed them with the ability to create a proinflammatory environment. Thus, our findings highlight that deleting negative regulators in CAR T cells presents a promising approach to target the inhibitory immune landscape of solid tumors. Based on our findings, we are developing an early-phase clinical study to evaluate the safety and efficacy of Reg-1 KO B7-H3-CAR T cells in patients with sarcoma.

### Limitations of the study

Our study has several limitations. First, we studied the non-cell-autonomous effects of Reg-1 KO in one specific B7-H3-CAR T cell product. Whether our findings extend to other B7-H3-CAR T cell products or CAR T cells targeting other antigens remains unknown. Second, Reg-1 KO induced significant B7-H3-CAR T cell expansion but did not improve their persistence. Future studies are needed to gain mechanistic insight into why Reg-1 KO B7-H3-CAR T cells did not persist. Third, we did not fully elucidate the mechanism by which Reg-1 KO B7-H3-CAR T cells reprogram the immunosuppressive TME, nor whether endogenous immune cells directly contribute to their improved antitumor activity. Finally, additional studies are needed to gain insights into the mechanism of tumor recurrence/progression since we only excluded the development of antigen loss variants in the present study.

## Resource availability

### Lead contact

Requests for further information and resources should be directed to and will be fulfilled by the lead contact, Stephen Gottschalk (stephen.gottschalk@stjude.org).

### Materials availability

This study did not generate new unique reagents.

### Data and code availability

The raw scRNA-seq data have been deposited in the NCBI GEO database and are accessible through the following GEO SuperSeries accession numbers: CAR T cells at 5 days after CAR T infusion (GEO: GSE295137) and endogenous CD45^+^ immune cells at 21 days after CAR T infusion (GEO: GSE295138). No custom computer code was generated or used in this study. Any additional information required to reanalyze the reported data is available from the lead contact upon request.

## Acknowledgments

We would like to acknowledge Nicole Chapman and Jana Raynor for reviewing the manuscript; Amanda George and St. Jude Center for In Vivo Imaging and Therapeutics for assistance with the animal studies; Mojtaba Zarei for assistance with the preparation of flow cytometry samples from mouse tissues; Melissa Hendren for Cas9 mice colony management; and the staff of the Center for Advanced Genome Editing (CAGE) for performing indel analyses. We thank the staff of the (1) Department of Immunology Flow Cytometry core for cell sorting of samples used for scRNA-sequencing, (2) Hartwell Center for Biotechnology for sequencing, and (3) Comparative Pathology Core for IHC and ISH analyses, with the latter two being in part supported by 10.13039/100000002National Institutes of Health (NIH)/10.13039/100000054National Cancer Institute (NCI) grant P30CA021765. This work was supported by NIH/NCI grant 1T32CA272387 (A.O.A.); R35CA253188, U01CA281868, and R01AI140761 (H.C.); R01CA292466 and 1R01CA288967 (J.T.Y.); and 10.13039/100000060National Institute of Allergy and Infectious Diseases (NIAID) grant U01AI176470 (S.Q.T.). Additional support was provided by St. Jude Collaborative Research Consortium on Novel Gene Therapies for Sickle Cell Disease (S.Q.T.) and the 10.13039/100012524American Lebanese Syrian Associated Charities (ALSAC) (L.J.T., G.K., C.D., S.Q.T., H.C., and S.G.). The content is solely the responsibility of the authors and does not necessarily represent the official views of the NIH.

## Author contributions

Conceptualization, A.O.A., H.C., and S.G.; methodology, A.O.A., H. Shi, S.S.P., T.C., H. Sheppard, P.Z., and S.M.P.-M.; investigation, A.O.A., H. Shi, P.N., S.S.P., T.C., X.S., P.Z., H. Sheppard, A.K., M.S.P., C.O'R., J.J.P., A.C., A.M., and G.L.; resources, J.T.Y., H.C., and S.G.; formal analysis, A.O.A., H. Shi, S.S.P., H. Sheppard, J.-Y.M., L.J.T., and S.G.; supervision, H.C. and S.G.; funding acquisition, G.K., C.D., H.C., and S.G.; writing – original draft preparation, A.O.A. and S.G.; writing – review and editing, A.O.A., H. Shi, S.S.P., H. Sheppard, P.N., X.S., P.Z., J.-Y.M., T.C., A.K., L.J.T., V.P., M.S.P., D.M.L., C.O'R., J.J.P., A.C., S.Z., A.M., G.L., S.Q.T., S.M.P.-M., L.J.T., J.T.Y., G.K., C.D., H.C., and S.G.

## Declaration of interests

S.Q.T. is co-inventor on patents covering GUIDE-seq and a member of the scientific advisory boards of Prime Medicine and Ensoma. G.K., C.D., H.C., and S.G. are co-inventors on patent applications or patents in the fields of cell or gene therapy for cancer. S.G. is a member of the Data and Safety Monitoring Board (DSMB) of Immatics. H.C. consults for Kumquat Biosciences and TCura Bioscience.

## Declaration of generative AI and AI-assisted technologies in the writing process

During the preparation of the graphical abstract, the authors used Perplexity to generate the initial version of the abstract. After using this tool/service, the authors reviewed and edited the content using Adobe Illustrator and take full responsibility for the content of the published graphical abstract.

## STAR★Methods

### Key resources table

**Table:** 

REAGENT or RESOURCE	SOURCE	IDENTIFIER
**Antibodies**		

BUV395 anti-mouse CD11b	BD Biosciences	Cat# 563553 RRID:AB_2738276
BUV615 anti-mouse CD11c	BD Biosciences	Cat# 751265 RRID:AB_2875281
BUV661 anti-mouse CD19	BD Biosciences	Cat# 612971 RRID:AB_2870243
BUV737 anti-mouse F4/80	BD Biosciences	Cat# 749283 RRID:AB_2873658
BUV805 anti-mouse CD4	BD Biosciences	Cat# 564922 RRID:AB_2739008
eFluor 450 anti-mouse CD62L	Thermo Fisher Scientific	Cat# 48-0621-82 RRID:AB_1963590
BV480 anti-mouse CD3	BD Biosciences	Cat# 746776 RRID:AB_2744035
BV570 anti-mouse Ly6C	BioLegend	Cat# 128030 RRID:AB_2562617
BV605 anti-mouse CD44	BD Biosciences	Cat# 563058 RRID:AB_2737979
BV711 anti-mouse CD8a	BD Biosciences	Cat# 563046 RRID:AB_2737972
BV786 anti-mouse CD45.1	BD Biosciences	Cat# 740889 RRID:AB_2740538
PerCP anti-mouse Ly6G	BioLegend	Cat# 127654 RRID:AB_2616999
BB700 anti-mouse CD279 (PD-1)	BD Biosciences	Cat# 566514 RRID:AB_2869777
PE anti-mouse CD274 (PD-L1)	BioLegend	Cat# 124308 RRID:AB_2073556
PE-Cy5.5 anti-mouse CD49b	Thermo Fisher Scientific	Cat# 35-5971-82 RRID:AB_2848327
PE-Cy7 anti-mouse CD206	BioLegend	Cat# 141720 RRID:AB_2562248
APC anti-mouse CD276 (B7-H3)	BioLegend	Cat# 135607 RRID:AB_2566062
Alexa Fluor 700 anti-mouse CD45.2	Thermo Fisher Scientific	Cat# 56-0454-82 RRID:AB_657752
APC-Cy7 anti-mouse NK1.1	BD Biosciences	Cat# 560618 RRID:AB_1727569
APC-Fire 810 anti-mouse CD45	BioLegend	Cat# 103174 RRID:AB_2860600
BUV395 anti-mouse CD45.1	BD Biosciences	Cat# 565212 RRID:AB_2722493
BUV496 anti-mouse CD24	BD Biosciences	Cat# 612953 RRID:AB_2870229
BUV563 anti-mouse CD44	BD Biosciences	Cat# 569705 RRID:AB_3626317
BUV615 anti-mouse CD11c	BD Biosciences	Cat# 751265 RRID:AB_2875281
BUV661 anti-mouse CD11b	BD Biosciences	Cat# 565080 RRID:AB_2722548
BUV737 anti-mouse CD8a	BD Biosciences	Cat# 612759 RRID:AB_2870090
BUV805 anti-mouse CD103	BD Biosciences	Cat# 741948 RRID:AB_2871259
BV421 anti-mouse CD163	BioLegend	Cat# 155309 RRID:AB_2814063
BV510 anti-mouse CD80	BioLegend	Cat# 104741 RRID:AB_2810337
BV605 anti-mouse CD86	BioLegend	Cat# 105037 RRID:AB_11204429
BV650 anti-mouse NK1.1	BD Biosciences	Cat# 564143 RRID:AB_2738617
BV711 anti-mouse CD206	BioLegend	Cat# 141727 RRID:AB_2565822
BV750 anti-mouse CD90.1	BD Biosciences	Cat# 746961 RRID:AB_2871744
BV785 anti-mouse XCR1	BioLegend	Cat# 148225 RRID:AB_2783119
SparkBlue550 anti-mouse CD19	BioLegend	Cat# 115566 RRID: AB_2832389
SparkBlue574 anti-mouse CD4	BioLegend	Cat# 100490 RRID:AB_2904268
PerCP-eFluor 710 anti-mouse CD25	Thermo Fisher Scientific	Cat# 46-0251-82 RRID:AB_2734935
Spark YG 593 anti-mouse F4/80	BioLegend	Cat# 157312 RRID: AB_2894428
PE-Dazzle594 anti-mouse CD276 (B7-H3)	BioLegend	Cat# 135612 RRID:AB_2750520
PE-Cy5 anti-mouse CD127	BioLegend	Cat# 135016 RRID:AB_1937261
PE-Cy7 anti-mouse CD366 (TIM3)	BioLegend	Cat# 119716 RRID:AB_2571933
PE-Fire810 anti-mouse MHC-Class II	BioLegend	Cat# 107667 RRID:AB_2894690
APC anti-mouse CD209a	BD Biosciences	Cat# 564928 RRID:AB_2739011
Alexa Fluor 647 anti-mouse CD64a/b	BD Biosciences	Cat# 558539 RRID:AB_647120
SparkNIR685 anti-mouse CD69	BioLegend	Cat# 104558 RRID:AB_2876411
APC-R700 anti-mouse CD152 (CTLA-4)	BD Biosciences	Cat# 565778 RRID:AB_2739350
APC-Fire750 anti-mouse CD223 (LAG3)	BioLegend	Cat# 125240 RRID:AB_2876449
Alexa Fluor 647 anti-mouse CD276 (B7-H3)	BD Biosciences	Cat# 562862 RRID:AB_2737848
BUV395 anti-mouse IL-17	BD Biosciences	Cat# 565246 RRID:AB_2722575
BUV737 anti-mouse IFNy	BD Biosciences	Cat# 612769 RRID:AB_2870098
BV421 anti-mouse RORgt	BD Biosciences	Cat# 562894 RRID:AB_2687545
BV510 anti-mouse CD25	BioLegend	Cat# 102042 RRID:AB_2562270
BV605 anti-mouse TCRb	BD Biosciences	Cat# 562840 RRID:AB_2687544
BV650 anti-mouse TNFa	BD Biosciences	Cat# 563943 RRID:AB_2738498
PerCP-eFluor 710 anti-mouse ARG1	Thermo Fischer Scientific	Cat# 46-3697-82 RRID:AB_2734843
PE anti-mouse IL-6	BioLegend	Cat# 504504 RRID:AB_315338
Alexa 594 anti-mouse TCF1	Cell Signaling Technology	Cat# 35972S RRID:AB_3750296
PE-Cy7 anti-mouse IL-10	BioLegend	Cat# 505026 RRID:AB_11150582
APC anti-mouse Foxp3	Thermo Fischer Scientific	Cat# 17-5773-82 RRID:AB_469457
Alexa Fluor 700 anti-mouse B220	Thermo Fischer Scientific	Cat# 56-0452-82 RRID:AB_891458
Alexa Fluor 647 anti-mouse BATF	Cell Signaling Technology	Cat# 47914S RRID:AB_3750297
BV785 anti-human CD4	Biolegend	Cat# 344642 RRID:AB_2728311
BV510 anti-human CD8	BD Biosciences	Cat# 563919 RRID:AB_2722546
BV650 anti-human CD45RO	Biolegend	Cat# 304232 RRID:AB_2563462
FITC anti-human CCR7	Miltenyi Biotec	Cat# 130-120-468 RRID:AB_2752108
BV605 anti-human CD39	Biolegend	Cat# 328236 RRID:AB_2750430
PerCP5.5 anti-human PD-1	Biolegend	Cat# 329920 RRID:AB_10960742
PE-Cy7 anti-human TIM3	Biolegend	Cat# 345014 RRID:AB_2561720
Rabbit Recombinant Monoclonal CD276 (B7-H3)	Abcam	Cat# ab313380 RRID:AB_3696894
Rabbit Recombinant Monoclonal ARGINASE 1	Cell Signaling	Cat# 93668 RRID:AB_2800207
Rat anti Mouse CD68	BioRad	Cat# MCA1957 RRID:AB_322219
Rabbit Monoclonal CD163	Abcam	Cat# ab182422 RRID:AB_2753196
BV785 Mouse IgG1, k Isotype Control	Biolegend	Cat# 400170 RRID:AB_11219601
BV510 Mouse IgG1, k	Biolegend	Cat# 400172 RRID:AB_2714004
BV650 Mouse IgG2a, k	Biolegend	Cat# 400266 RRID: AB_11203909
FITC Human IgG1, REA Control	Miltenyi Biotech	Cat# 130-113-437 RRID:AB_2733689
BV605 Mouse IgG1, k	Biolegend	Cat# 400162 RRID:AB_11125373
PerCP5.5 Mouse IgG1, k	Biolegend	Cat# 400150 RRID:AB_893664
PE-Cy7 IgG1, k	Biolegend	Cat# 400126 RRID:AB_326448
Purified NA/LE Hamster anti-mouse CD3	BD Biosciences	Cat# 553057 RRID:AB_394590
Purified NA/LE Hamster anti-mouse CD28	BD Biosciences	Cat# 553294 RRID:AB_394763
Alexa-Flour 647 anti-mouse F(ab)2 fragment specific	Jackson ImmunoResearch	Cat# 115-606-006 RRID:AB_2338922

**Bacterial and virus strains**		

Mix & Go! *E. coli* Cells DH5α	Zymo Research	Cat# T3007

**Biological samples**		

Human peripheral blood mononuclear cells (PBMCs)	Charles River Lab	N/A

**Chemicals, peptides, and recombinant proteins**		

Live/Dead Fixable Blue	Thermo Fischer Scientific	Cat# L23105 RRID:AB_3717566
PB450 4′,6-diamidino-2-phenylindole (Dapi)	BD Biosciences	Cat# 564907 RRID:AB_2869624
PE-Labeled Human CD276 (B7-H3) chimeric protein	Acro Biosystems	Cat# B7B-HP2H9
DMEM media	(GE Healthcare Life Sciences	Cat# SH30081.01
Fetal bovine serum (FBS)	Gibco	Cat# 26140-079
Glutamax	Gibco	Cat# 35050061
Penicillin-streptomycin	Gibco	Cat# 15140122
PBS	Gibco	Cat# 14190-144
Collagenase IV	STEMCELL Technologies	Cat# 07427
DNase I	Sigma Aldrich	Cat# 10104159001
Ammonium chloride (ACK) Lysis buffer	Gibco	Cat# A1049201
Recombinant Human IL2	PeproTech	Cat# 200-02-1MG
Recombinant Human IL7	PeproTech	Cat# 200-07-01M
Recombinant Human IL15	PeproTech	Cat# 200-15-1MG
TransAct reagent	Miltenyi Biotech	Cat# 130-111-160
X-VIVO 15 media	Lonza	Cat# BP04-744Q
Human AB serum	Valley Biomedicals	Cat# HUMANABSRMC-HI-1
Recombinant human IL-7	Miltenyi Biotech	Cat# 130-095-367
Recombinant human IL-15	Miltenyi Biotech	Cat# 130-095-760
MaxCyte Electroporation Buffer	GE Healthcare Life Science	Cat# EPB-1
Ionomycin calcium salt	Sigma-Aldrich	Cat# I0634-1MG
Phorbol 12-myristate 13-acetate	Sigma-Aldrich	Cat# P8139
MEM-NEAA (100x)	Gibco	Cat# 11140050
2-Mercaptoethanol	Gibco	Cat# 21985-023
Gene juice	Millipore Sigma	Cat# 70967-3
Zymo Pure	Zymo Research	Cat# D4202

**Critical commercial assays**		

MycoAlert Mycoplasma Detection kit	Lonza	Cat#LT07-318
Naive CD8 T cell isolation kit	Miltenyi Biotec	Cat# 130-096-543
Pan T cell isolation kit	Miltenyi Biotec	Cat# 130-095-130
32-plex Milliplex mouse cytokine/chemokine magnetic bead panel	Millipore Sigma	Cat# MCYTMAG-70K-PX32
41-plex Milliplex mouse cytokine/chemokine magnetic bead panel	Millipore Sigma	Cat# MCYT1-190K-SPX
Human Cytokine Kit	Bio-Techne	Cat# 220601046
CellTiter96 AQueous One Solution Cell Proliferation Assay kit	Promega	Cat# G3582
BD Cytofix/Cytoperm kit	BD Biosciences	Cat# 554714
Chromium Next GEM Single Cell 5ʹ Kit v2	10x Genomics	Cat# PN-1000263
Chromium Single Cell Mouse TCR Amplification Kit	10x Genomics	Cat# PN-1000254
RNAscope VS Universal AP Reagent Kit (RED)	Advanced Cell Diagnostics	Cat# No. 323250
mRNA RED Detection Kit	Roche Diagnostics	Cat# 760-234
mRNA Sample Prep Kit	Roche Diagnostics	Cat# 760-248
mRNA RED Probe Amplification Kit	Roche Diagnostics	Cat# 760-236
Golgi Plug Protein Transport Inhibitor	BD Biosciences	Cat# 51-2301KZ
Golgi Stop Protein Transport Inhibitor	BD Biosciences	Cat# 51-2092KZ
Quantum™ Alexa Fluor® 647 MESF	Bang Laboratories	Cat# 647A

**Deposited data**		

scRNA-seq data for CAR T cells on day 5	NCBI GEO database	GEO: GSE295137
scRNA-seq data for CD45^+^ Immune cells on day 21	NCBI GEO database	GEO: GSE295138

**Experimental models: Cell lines**		

F331 (Col2.3-Cre, p53fl/+)	Zhao et al.^78^	N/A
M1199 (Col2.3-Cre; TP53fl/fl; LSL-cMycT58A)	Nirala et al.^60^	N/A
LLC	ATCC	Cat# CRL-1642
LM7	Jia et al.^79^	N/A
B7-H3 KO F331	This study	N/A
B7-H3 KO LM7	Nguyen et al.^27^	N/A
LM7 GFPffluc	Ahmed et al.^80^	N/A
THP1	ATCC	Cat# TIB-202
KG1a	ATCC	Cat# CCL-246.1
Platinum-E cells	Cell Biolabs	Cat# RV-101
GP + E−86 cells	ATCC	Cat# CRL-9642

**Experimental models: Organisms/strains**		

B6J.129(Cg)-*Gt(ROSA)26Sor*^*tm1.1(CAG-cas9*^*∗*^*,-EGFP)Fezh*^/J	The Jackson Laboratory	Cat# 026179
B6.PL-*Thy1*^*a*^/CyJ	The Jackson Laboratory	Cat# 000406
C57BL/6J	The Jackson Laboratory	Cat# 000664
NOD.Cg-PrkdcscidIL2Rgtm1wjl/SzJlnv (NSG)	Breeding colony, St Jude Children’s Research Hospital	N/A

**Oligonucleotides**		

Murine non-targeting control sgRNA: 5′-ATGACACTTACGGTACTCGT-3′	Wei et al.^10^	N/A
Murine Regnase-1 sgRNA: 5′-AAGGCAGTGGTTTCTTACGA-3′	Wei et al.^10^	N/A
AAVS1 sgRNA: 5′-GGGAACCCAGCGAGTGAAGA-3′	Mali et al.^81^	N/A
Human REGNASE-1 sgRNA: 5′-TTCACACCATCACGACGCGT-3′	Zheng et al.^11^	N/A
murine *Ppib* RNA	Advanced Cell Diagnostics	Cat# 313919
Bacteria gene *dapB* RNA	Advanced Cell Diagnostics	Cat# 312039

**Recombinant DNA**		

Anti-mouse B7-H3-CAR	Haydar et al.^72^	N/A
Anti-mouse SP6-CAR	Haydar et al.^72^	N/A
Anti-human B7-H3 CAR	Nguyen et al.^27^	N/A
Anti-human ΔB7-H3 CAR	Nguyen et al.^27^	N/A

**Software and algorithms**		

OMIQ online software suite	Dotmatics	https://www.omiq.ai/
Flow jo software (v.10.8.1)	FlowJo, LLC	https://www.flowjo.com/
GraphPad Prism (v.10.4.0)	GraphPad software	https://www.graphpad.com/
xPONENT® Software	Luminex	https://us.diasorin.com/en/luminex
Belysa Immunoassay Curve Fitting Software	Merck	https://www.merckmillipore.com/
SnapGene	Dotmatics	https://www.snapgene.com/
IVIS-200 imaging system	Perkin Elmer	https://www.perkinelmer.com/
Living Image Software	Perkin Elmer	https://www.perkinelmer.com/
BioRender	BioRender	https://www.biorender.com/
R package Seurat (v4.1)	Hao et al.^82^	https://satijalab.org/seurat/
scRepertoire package (v1.3.5)	Borcherding et al.^83^	https://www.borch.dev/uploads/screpertoire/
CellChat (v1.4.0)	Jin et al.^84^	https://github.com/jinworks/CellChat
GSEA command line software (v4.0.3) and MSigDB (v7.3)	Subramanian et al.^85^	https://www.gsea-msigdb.org/gsea/index.jsp
Cell Ranger v6.0 Single-Cell software suite (10x Genomics)	Zheng et al.^86^	https://github.com/10XGenomics/cellranger
Qiagen IPA	Kramer et al.^87^	https://digitalinsights.qiagen.com/products-overview/discovery-insights-portfolio/analysis-and-visualization/qiagen-ipa/
HALO v3.6.4134	Indica Labs	https://indicalab.com/halo/
HALO AI 3.6.4134	Indica Labs	https://indicalab.com/halo-ai/

### Experimental models and study participants

#### Animal models

All animal experiments were performed according to an approved protocol by St. Jude Children’s Research Hospital Institutional Animal Care and Use Committee. Eight-to ten-week-old male and female mice maintained under ambient temperature and humidity with a 12/12 h (h) on/off light cycle were used for the F331 and M1199 models. C57BL/6 and Rosa26-Cas9 knock-in^88^ mice were purchased from Jackson Laboratory. *F331 model:* 1 × 10^6^ cells were injected intravenously (i.v.) on day 0 into C57BL/6 mice. 1 × 10^6^ Reg-1 KO SP6-, Ctrl KO B7-H3-, or Reg-1 KO B7-H3-CAR T cells in sterile PBS were injected i.v. on day 7; cell dose for experiment with CAR T cells derived from CD4^+^/CD8^+^-selected T cells: 1.5 × 10^6^ T cells. *M1199 model:* 2 × 10^5^ cells were injected i.v. on day 0 into C57BL/6 mice. 2 × 10^6^ Reg-1 KO SP6-, Ctrl KO B7-H3-, or Reg-1 KO B7-H3-CAR T cells in sterile PBS were injected i.v. on day 7. *LLC model:* 1 × 10^5^ or 5 × 10^5^ cells were injected i.v. on day 0 into C57BL/6 mice. 2 × 10^6^ Reg-1 KO SP6-, Ctrl KO B7-H3-, or Reg-1 KO B7-H3-CAR T cells in sterile PBS were injected i.v. on day 7. Weekly body weight measurements and routine health checks were performed. Subsets of mice were bled retro-orbitally to collect blood for complete blood count analysis (Genesis ™ analyzer) and cytokine/chemokine analysis (Luminex Corporation). The humane study endpoint was met when mice lost about >20% of initial body weight or showed signs of disease or discomfort. To determine antitumor activity, mice were euthanized on day 21 (F331) or day 28 (M1199) post T cell infusion.

*F331 subcutaneous model:* mice received 2 × 10^6^ F331 cells in the right flank. On day 7, mice were administered a single i.v. dose of either 5 × 10^6^ Ctrl KO- or Reg-1 KO B7-H3-CAR T cells via tail vein injection. Tumor kinetics were monitored by weekly caliper measurements. The humane study endpoint was met when mice i) lost 20% of body weight, ii) showed signs of distress iii) tumor burden reached 20% of total body mass (≥2,500 mm^3^) or iv) when recommended by veterinary staff.

For flow cytometric and scRNA-seq analyses, mice were euthanized and single cell suspension of lungs and spleens were prepared. Briefly, lungs and spleens were isolated from osteosarcoma bearing mice and single cell suspensions of lungs were prepared by mechanical dissociation and enzymatic digestion with collagenase IV (0.5 mg/mL, STEMCELL Technologies) and DNase I (25 IU/mL, Sigma Aldrich). Cell suspension was incubated at 37°C for 30 min and then passed through a 70 μm filter to remove debris. Cell supernatants were spun to obtain cell pellets. Ammonium chloride (ACK) lysing buffer (Gibco) was added to the cell pellet to remove red blood cells. Spleens were only mechanically dissociated and passed through a 70 μm filter before red blood cell lysis. Cells were washed with PBS and resuspended in flow cytometry staining buffer.

*Xenograft models:* The intraperitoneal and orthotopic xenograft models with LM7 GFPffluc cells have been previously described.^27^^,^^49^ All experiments utilized NOD-scid IL2Rgamma^null^ (NSG) mice obtained from St. Jude’s NSG colony. For imaging, mice were injected intraperitoneally (i.p.) with 150 mg/kg of D-Luciferin (PerkinElmer) 5–10 min before imaging, anesthetized with isoflurane, and imaged under anesthesia with a Xenogen IVIS-200 imaging system. Image quantification was performed by using Living Image software (PerkinElmer). The study endpoint was met when flux values reached >1 × 10^10^ or mice met humane endpoints (described above).

#### Cell lines

The murine F331 (Col2.3-Cre, p53^fl/+^) and M1199 (Col2.3-Cre; TP53fl/fl; LSL-MycT58A) OS cell lines were derived from genetically engineered mouse models (GEMM) that previously have been published.^60^^,^^78^ The Lewis lung carcinoma (LLC) was originally purchased from ATCC (CRL-1642). The lung metastatic human OS cell line LM7 was kindly provided by Dr. Eugenie Kleinerman (MD Anderson Cancer Center, Houston TX) in 2011, and the generation of the B7-H3 KO LM7 cell line was previously reported.^27^^,^^79^ Cell lines were cultured in DMEM media (GE Healthcare Life Sciences, Chicago, IL) supplemented with 10% fetal bovine serum (FBS), 1% glutamax and 1% penicillin-streptomycin. ATCC short-tandem repeat profiling service was used to authenticate cell lines. Cell lines were routinely screened for mycoplasma by using MycoAlert Mycoplasma Detection kit (Lonza, Walkersville, MD). Cell lines were maintained in culture for a maximum of 2 months before being replaced with a fresh vial of cells. B7-H3 KO F331 cells (F331-KO) were generated using CRISPR/Cas9 technology at St. Jude Center for Advanced Genome Engineering (CAGE) as previously described using an established protocol^27^^,^^89^ with precomplexed ribonuclear proteins (RNPs) using a sgRNA targeting B7-H3 (CAGE193.CD276.g22, 5′-UGUCCACCAGGGCCACCACG-3′, Synthego). Cells were clonally expanded and screened for the desired modification (out-of-frame indels and a premature stop codon) via targeted deep sequencing using gene specific primers with partial Illumina adapter overhangs (CAGE193.DS.F – 5′-CTACACGACGCTCTTCCGATCTacctgaaaggaagcgtgtgcagagc-3′ and CAGE193.DS.R-5′- CAGACGTGTGCTCTTCCGATCTagctgccctcgtcggttactcggac-3′, overhangs shown in uppercase) as previously described.^89^ NGS analysis of clones was performed using CRIS.py.^90^ Final clones were authenticated using the PowerPlex Fusion System (Promega) performed at St. Jude Hartwell Center for Biotechnology. Knockout was confirmed by flow cytometric analysis.^90^

### Method details

#### Generation of retroviral vectors

The generation of the retroviral vectors encoding B7-H3- or SP6-CAR.CD28.ζ was previously described.^72^^,^^91^ The B7-H3-CAR utilizes a scFv as an antigen binding domain that is derived from the murine B7-H3-specific monoclonal antibody (mAb) m276 (affinity: 24 nM).^92^ To generate retroviral particles, GPE+86 producer cell line was cultured in IMDM media supplemented with 10% FBS and 1% glutamax to generate retroviral particles for murine T cell transduction. Cell supernatant was collected after 48 h, spun at 1,000g for 5 min and passed through a 0.45μm filter. Fresh supernatants or leftover supernatant snap-frozen at −80°C was used for chimeric antigen receptor (CAR) T cell transduction. The generation of the retroviral vectors encoding sgRNAs (non-targeting control (Ctrl) sgRNA, ATGACACTTACGGTACTCGT or Reg-1 sgRNA, AAGGCAGTGGTTTCTTACGA) and ametrine were previously reported.^10^ To generate retroviral particles, the Platinum E retroviral packaging cell line (Cell Biolabs, Inc.) was co-transfected with sgRNA-encoding retroviral vectors and helper plasmid pCL-Eco (Addgene no. 12371).

#### Generation of murine CAR T cells

Naive CD8^+^ T cells were enriched from the spleen and lymph nodes of Cas9 (CD45.1) transgenic C57BL/6 mice using a naive CD8 T cell isolation kit (Miltenyi Biotec, Bergisch Gladbach, Germany). Similarly, CD4^+^/CD8^+^-selected T cells were enriched from the spleen of Cas9 (CD45.1) transgenic C57BL/6 mice using a Pan T cell isolation kit (Miltenyi Biotec, Bergisch Gladbach, Germany). Enriched T cells were activated in the presence of murine CD3 (1μg/mL, BD Biosciences) and CD28 (2μg/mL, BD Biosciences) antibodies and cultured with human IL2 (50IU/mL, PeproTech) in complete RPMI media for 48h. Reg-1 or non-targeting single guide RNA (Ctrl) KO B7-H3-CAR or Reg-1 KO SP6-CAR T cells were generated by co-transduction of activated naive CD8^+^ T cells with retroviral vectors encoding CARs or the respective guide RNA + ametrine. Cells were cultured in the presence of human IL2 (50IU/mL, PeproTech) for 48h while non-transduced (NT) naive CD8^+^ T cells served as a control. Cells were expanded in the presence of IL7 (10ng/mL, PeproTech) and IL15 (10ng/mL, Peprotech) for another 2–3 days. Editing efficacy was confirmed by indel analysis and double transduction by flow cytometry. CAR T cells were used for experiments on or before day 6 post-transduction.

#### Generation of human CAR T cells

Deidentified leukapheresis products from healthy donors were purchased from Charles River Laboratories and CD4^+^ and CD8^+^ T cells were selected on a CliniMACS Plus instrument Miltenyi Biotech) prior to cryopreservation. St Jude Children’s Research Hospital Institutional Review Board reviewed the research activity and determined it to be non-human subject research since the leukapheresis products were de-identified. T cell activation and transduction followed a previously published procedure,^93^ which was modified to include a gene-editing step. Briefly, thawed T cells were activated with T cell TransAct reagent (Miltenyi Biotech) and cultured overnight in X-VIVO 15 media supplemented with 5% Human AB serum (Valley Biomedical), recombinant human IL-7 (10ng/mL, Miltenyi Biotech) and IL-15 (10ng/mL, Miltenyi Biotech) (T cell media). On day 1, activated T cells were transduced with a previously described lentiviral vector encoding a B7-H3-CAR.CD28.ζ at a multiplicity of infection of 25 using protamine sulfate (4μg/mL) for 24 h.^27^ The B7-H3-CAR utilizes a scFv as an antigen binding domain that is derived from the human B7-H3-specific mAb MGA271 (affinity: 1.4 nM).^94^ On day 2, following a gentle wash with MaxCyte Electroporation Buffer (GE Healthcare Life Science), cells were mixed with precomplexed 4 μM AAVS1 (sgRNA 5′-GGGAACCCAGCGAGTGAAGA-3′) or REGNASE-1 (sgRNA 5′– TTCACACCATCACGACGCGT-3′) RNPs at a Cas9:sgRNA ratio of 1:2 and then electroporated with a MaxCyte GTx Electroporation System. Subsequently, T cells were expanded in G-Rex (Wilson Wolf) cell culture devices in T cell media. On day 5, cytokines were replenished, and on day 7, CAR T cells were harvested and directly used for analysis or cryopreserved for future use. Off-target gene editing analysis was performed using GUIDE-seq-2.^95^ For *in vivo* experiments, CAR T cells were thawed and cultured for 2 days in T cell media, washed and resuspended in PBS before injecting into mice.

#### Coculture assays

Cell culture supernatants from murine CAR T cells and non-transduced T cells were collected 4 days post-transduction or obtained from a co-culture of CAR T cells and tumor cells at an effector to target (E:T) ratio of 1:1 after 48 h. Serum was obtained from tumor-bearing mice that received CAR T cell therapy or PBS on day 7. All samples were snap-frozen at −20°C before evaluating cytokine/chemokine production with a 32-plex- MCYTMAG-70K-PX32 (F331) or 41-plex-MCYT1-190K-SPX (M1199 and LLC) Milliplex mouse cytokine/chemokine magnetic bead panel (Millipore Sigma). Analysis was performed using a Luminex FlexMap 3D instrument and software (Luminex Corporation). Human CAR T cells supernatants were collected after coculture with tumor cells for 24 h and cytokine production was determined using Ella (Bio-Techne, serial #220601046).

#### Cytotoxicity assays

The cytolytic activity of murine CAR T cells was assessed with a CellTiter96 AQueous One Solution Cell Proliferation Assay kit (Promega). In a 96-well tissue culture treated plate, 10,000 (F331, F331 B7-H3 KO) or 8,000 (M1199, LLC) cells were co-cultured with serial dilutions of CAR T cells at 37°C for 48 h. Media, CAR T cells and tumor cells alone served as controls. Each condition was performed in technical triplicates. The media and CAR T cells were removed by slowly pipetting up and down without disturbing the adherent tumor cells. CellTiter96 AQueous One Solution Cell Proliferation reagent in complete DMEM was added into each well in the plate and incubated at 37°C for 2 h. The percentage of viable tumor cells after exposure to CAR T cells was calculated by the formula: (Sample-media alone)/(Tumor alone -media alone) x 100. The cytolytic activity of human CAR T cells was assessed with a 24 h xCelligence assay at an E:T ratio of 1:1 using LM7 or LM7-B7-H3 KO target cells. As indicated, a ffluc-based cytotoxicity with B7-H3 positive (THP1) and B7-H3 negative (KG1a) target cells was also utilized.^96^

#### Flow cytometry

Cell surface staining was conducted using 5% FBS in PBS (Lonza) as the antibody diluent and cell wash medium. Cells were labeled at 2 × 10^6^ cells per mL with optimally titrated antibody solutions and incubated at 4°C for 30 min. Unless noted otherwise, dead cells were excluded through reaction with Live/Dead Fixable Blue dead cell stain (Thermofisher) according to the manufacturer’s protocol, except where otherwise stated.

##### B7-H3 and CAR detection

Matched isotypes or known negatives (such as non-transduced (NT) T cells) served as gating controls. B7-H3 detection was performed using anti-mouse CD276-APC (1:200 dilution, clone MIH32, BD Biosciences). B7-H3 surface molecule expression was quantified using the Quantum Alexa Fluor 647 Microsphere Kit (Bangs Laboratories, Inc.). A total of 10,000 events were acquired for each of four microsphere bead populations labeled with known increasing numbers of Alexa Fluor 647 molecules, combined in the same tube, while blank microspheres were acquired in a separate tube. All samples were analyzed using identical instrument settings as cells. B7-H3 fluorescence intensity calibration and quantification were performed using the Bangs Laboratories quantitative analysis template, QuickCal v3.0.

CAR expression was determined using anti-mouse F(ab’)2 fragment specific antibody (1:200 dilution; polyclonal, Jackson ImmunoResearch). Dead cells were excluded by staining cells with 4′,6-diamidino-2-phenylindole (DAPI) before data acquisition. A FACSCanto II instrument (BD Biosciences) equipped with 405, 488, and 633nm lasers, and running BD FACS DIVA v.9.0 software was used to acquire the flow cytometry data.

##### Cell surface staining of single cell suspensions from lungs and spleens

Cells were stained with fluorochrome-conjugated antibodies using combinations of the markers shown in Tables S2 and S4. Flow cytometric analysis was conducted using a 5-laser Cytek Aurora cytometer (Cytek Biosciences, Fremont, CA, USA) equipped with 355, 405, 488, 561, and 633 nm lasers and 56 fluorescent detectors, utilizing SpectroFlo software (Cytek) for data acquisition and spectral unmixing. Dimensional reduction and additional analyses were conducted on unmixed FCS files using the OMIQ online software suite (Dotmatics, Boston, MA, USA).

##### Intracellular cytokine staining of single cell suspensions from lungs

Cells were stimulated with ionomycin calcium salt (1 μg/mL, Sigma-Aldrich) and phorbol 12-myristate 13-acetate (PMA, 50 ng/mL, Sigma-Aldrich) for 1 h. Brefeldin A (2μL of 1000x) was then added for an additional 3 h to block cytokine secretion. Stimulation and blocking of protein transport were performed at 37°C in 5% CO_2_. Cells were collected from culture and washed in staining buffer at 4°C, then labeled for surface epitope expression as described above. Afterward, cells were fixed and permeabilized using the BD Cytofix/Cytoperm kit (BD Biosciences) as per product instructions. Immunofluorescent staining against intracellular antigens was performed. Surface and intracellular staining were conducted with antibodies detailed in Table S6. Flow cytometric data was acquired using a Cytek Aurora cytometer described above and spectrally unmixed FCS files were analyzed using the FlowJo software (v10.8.1).

##### B7-H3-CAR and cell surface staining of human CAR T cells

Single cell suspension of CAR T cells was stained with B7-H3 chimeric protein and antibodies detailed in Table S7 to evaluate the CAR expression, memory and exhaustion phenotype of generated human CAR T cells. Matched isotypes or known negatives (non-transduced T cells) serve as controls while DAPI was used to exclude dead cells. Flow cytometry data was acquired on Cytoflex Flow Cytometer and analyzed using FlowJo software (v10.8.1).

#### scRNA-seq sample preparation

*scRNA-Seq sample preparation and bioinformatics analysis:* Ctrl or Reg-1 KO CAR T cells were infused into F331-tumor bearing mice (see animal models section). Lung and spleen were isolated and processed for single cell suspension. In the experiment to profile CAR T cells on day 5 post infusion, lungs or spleens from three mice per treatment group were pooled together. Ctrl or Reg-1 KO CAR T cells were sorted based on CD45.1, CD8 and Ametrine expression. To investigate the lung OS immune landscape 21 days post CAR T cell infusion, two mice per treatment group were euthanized and lung was processed individually. Sorted CD45-positive and CD45-negative cells from lung Ctrl or Reg-1 KO at a ratio of 3:1 was processed for scRNA-seq experiment. Dead cells were excluded by staining with DAPI before acquisition. Cells were sorted on a BD FACS Aria III cell sorter (BD Biosciences). The samples were centrifuged at 2,000 rpm for 5 min, after which cells with over 98% viability were resuspended in 1× PBS (Thermo Fisher Scientific) containing 0.04% BSA (Amresco), at a final concentration of 1 × 10^6^ cells per mL. Single-cell suspensions were then loaded into a Chromium Controller (10x Genomics) to produce approximately 10,000 single-cell gel bead emulsions per sample, with each sample processed in a separate channel. Library preparation was carried out using the Chromium Next GEM Single Cell 5′ v2 (dual index) and Gel Bead kit (10x Genomics). Amplified cDNA (11 cycles) was either used for TCR enrichment and library construction with the Chromium Single Cell V(D)J TCR kit or for gene expression library preparation. The quality of the cDNA and resulting libraries was assessed using a high-sensitivity DNA chip on a 2100 Bioanalyzer (Agilent Technologies). Sequencing of the 5′ libraries was performed on an Illumina NovaSeq system, with paired end reads consisting of 26 cycles for read 1 and 90 cycles for read 2. On average, gene expression libraries yielded 50 million reads per sample, while TCR libraries generated 5 million reads per sample.

##### Data preprocessing

The Cell Ranger v6.0 Single-Cell software suite (10x Genomics) was used to process the scRNA-seq FASTQ files. The ‘cellranger count’ command was performed to align the raw FASTQ files to a custom mouse reference genome (mm10 with the addition of the mouse B7-H3-CAR sequence) and summarize the data into matrices that describe gene read counts per cell. For the datasets with matched TCR sequencing data, the ‘cellranger vdj’ command was used to generate a count matrix, which, after filtering, was used for downstream analyses.

For gene expression sequencing, the filtered count matrices were read into the R package Seurat (v4.1). Samples were merged into single Seurat objects for consistent filtering, and features detected in fewer than three cells were removed from the dataset. Cells with abnormally low features or unique molecular identifier (UMI) counts or high mitochondrial read percentages (potentially dead or damaged cells) were removed. Cells with abnormally high UMI counts (potentially multiple cells in a single droplet) were also removed. Finally, any remaining multiplets expressing mutually exclusive marker genes were removed.

For Ctrl or Reg-1 KO CAR T cells from lungs and spleens on day 5 post infusion, 7,282 lung derived CAR T cells were retained with an average of 3,533 genes per cell (UMI median: 13,228; range: 1,581–59,735) and 5,059 spleen derived CAR T cells were retained with an average of 3,444 genes per cell (UMI median: 11,336; range: 1,477–59,877). To minimize the impact of this imbalance in cell accumulation between Ctrl or Reg-1 KO CAR T cells, a random subsampling procedure was applied on the Reg-1 KO CAR T cell dataset to match the number of Ctrl KO cells (286 cells for each genotype) and the scRNA-seq analyses were repeated.

For tumor microenvironment cells on 21 days post Ctrl or Reg-1 KO CAR T cell infusion, 13,255 cells were retained with an average of 1,933 genes per cell (UMI median: 4,495; range: 500–49,880). After quality control, libraries were normalized with the Normalize Data function (scale factor = 1×10^6^) in the Seurat R package.

#### Pathological examination

Mice injected with F331, M1199 or B7-H3 KO F331 were euthanized at different timepoints post T cell infusion and submitted to the St. Jude Children’s Research Hospital Comparative Pathology Core Laboratory for histological assessment to ascertain the extent of metastatic disease and evaluate pulmonary microenvironment. The mice were necropsied following euthanasia, and lungs were immersed in 10% neutral-buffered formalin for at least 48 h. After fixation, tissues were embedded in paraffin, sectioned at 4 μm, mounted on positively charged glass slides (Superfrost Plus, Thermo Fisher Scientific), and dried at 60°C for 20 min before dewaxing and staining with H&E. Pathology evaluation was conducted in a blinded manner to the experimental condition. HE, IHC, and ISH images were scanned with a Pannoramic 250 Flash III Scanner (3DHistec) and imaged and analyzed using HALO v3.6.4134 and HALO AI 3.6.4134 (Indica Labs, Inc. Albuquerque, NM). Antibodies used for IHC are listed in Table S8.

#### In situ hybridization (ISH)

H&E-stained section of the lungs and spleens were immunolabeled with a customized RNAscope™ 2.5 VS probe set to detect murine B7-H3-CARs (CAR-scFv-m276-C1, Cat No.1220899-C1, Advanced Cell Diagnostics Inc., Hayward CA). Staining was performed on a Ventana Discovery Ultra automation system (Ventana Medical Systems, Inc., Tucson, AZ) using the RNAscope VS Universal AP Reagent Kit (RED) (Advanced Cell Diagnostics, Inc., Hayward, CA) and the following reagents from Roche Diagnostics mRNA RED Detection Kit (760–234), mRNA Sample Prep Kit (760–248), and mRNA RED Probe Amplification Kit (760–236) according to manufacturer’s protocols. The hybridization signals were detected by chromogenic development with fast red, followed by counterstaining with hematoxylin. Each sample was quality controlled for RNA integrity with a RNAScope probe specific to murine *Ppib* RNA (313919, Advanced Cell Diagnostics, Inc., Hayward, CA) and for background with a probe specific to bacteria gene *dapB* RNA (312039, Advanced Cell Diagnostics, Inc., Hayward, CA). Specific RNA staining signal was identified as red punctate dots. Pathology evaluation was conducted in a manner that was blinded to the experimental condition.

### Quantification and statistical analysis

The sample numbers (n), biological replicates, *p* value and statistical methods are shown in the figure legends. GraphPad Prism software v.10.4.0 was used to plot all graphs and perform statistical analyses. Data are presented as mean ± standard error of mean (±SEM).

#### scRNA-seq cluster annotation and data visualization

Normalized and filtered data were processed using the standard Seurat pipeline (v4.1). UMAP dimensionality reduction was used for visualization, and Seurat’s FindClusters function (v4.1) was used to separate cells into unsupervised clusters. Cell types in clusters were defined using published cell type signatures^44^^,^^97^^,^^98^: CD4^+^ T cells, CD8^+^ T cells, B cells, NK cells, macrophages, cDC1, cDC2, mregDC, and neutrophils signatures^44^; γδT cell signature^98^; endothelial cells and fibroblasts signatures.^97^ For analysis of CD8^+^ T cells, *Cd3d*^+^*Cd8a*^+^*Cd4*^-^ cells were isolated and re-clustered using the Seurat workflow. Activity scores of signatures associated with T cell activation,^45^ cell-cycle^99^ and memory-precursor^100^ states were calculated by AddModuleScore function in Seurat R package. For analysis of macrophages, cells were reclustered into eight subclusters with a heatmap highlighting subcluster-specific marker genes, which was plotted by DoHeatMap function in Seurat R package. Subclusters 0 (*Vcan*^+^*Thbs1*^+^), 1 (*Syngr1*^+^*Ms4a7*^+^), 4 (*Ace*^+^*Pglyrp1*^+^), 5 (*Folr2*^+^*Lyve1*^+^), 6 (*Plet1*^+^*Ly75*^+^) and 7 (*Cxcr2*^+^*Mmp9*^+^) are defined by two top subcluster-specific markers.

#### Identification of ligand–receptor pairs in scRNA-seq data

To determine the differences in cell-cell interaction strengths in tumor microenvironment between Ctrl or Reg-1 KO CAR T cell treated lung, CellChat (v1.4.0) was used. CellChat objects were produced using the CreateCellChat function. The overall cell–cell interaction strengths between different cell types was calculated by the default workflow based on ligand–receptor databases in the CellChat package. After running the pipeline on the two genotypes separately, the two CellChat objects were merged using the mergeCellChat function. Heat maps of the differential cell–cell interaction strength between genotypes were generated using the function netVisual_heatmap (measure = ‘weight’), with row *Z* score to indicate the difference in the interaction strength on the heatmap.

#### Differential gene expression analysis

Differential gene expression analysis was performed on the log-normalized gene expression matrices with the Seurat FindMarkers function (v4.1) by using a two-tailed Wilcoxon rank-sum test and calculating adjusted *P* (*P*adj) values via Bonferroni correction.

#### GSEA analysis

For GSEA, genes were ranked in order of descending log (fold change) values (derived from the differential expression analysis). Pre-ranked GSEA was performed using the broad GSEA command line software (v4.0.3). The Hallmark, C2 and C5 geneset collections from MSigDB (v7.3) were used.

#### IPA upstream regulator inference

Top 250 genes for up- and down-regulated genes (500 genes in total) in each differential gene expression comparison are used as the input for Ingenuity Pathway Analysis (IPA, Qiagen) after the Log2 fold change cut-off, followed by “Core Analysis”.

#### Single-cell TCR sequencing analysis

Filtered contig annotation matrices from the Cell Ranger output were loaded into R. Annotation and quantification of TCR clonotypes in CD4^+^Foxp3^–^, CD8^+^ and CAR T cells were processed with the scRepertoire package (v1.3.5). Clonal frequencies were categorized as follows: single (1), small (2–5) and medium (6–20).

## Supplemental information

Document S1. Figures S1–S34 and Tables S1–S4 and S6–S8

Table S5. List of FlowSOM clusters from 38 fluorophore flow cytometry panel, related to Figure 4 and Figures S21–S24

Document S2. Article plus supplemental information
