# Supplementary material for: Targeting Regnase-1 in B7-H3-CAR T cells reprograms the tumor microenvironment and enhances antitumor efficacy for osteosarcoma
Source: Cell Rep Med. 2026 Aug 25;7(9):103008. doi: 10.1016/j.xcrm.2026.103008 (PMC13589500; doi:10.1016/j.xcrm.2026.103008)
Supplement: Supplementary file 1 [file mmc1.pdf]

Cell Reports Medicine, Volume *7*

Supplemental information

Targeting Regnase-1 in B7-H3-CAR T cells reprograms the tumor microenvironment and enhances antitumor efficacy for osteosarcoma

Adeleye O. Adeshakin, Hao Shi, S. Scott Perry, Heather Sheppard, Phuong Nguyen, Xiang Sun, Peipei Zhou, Jean-Yves Métais, Trevor Cunningham, Anil KC, Liqing Tian, Vivek Peche, Mollie S. Prater, Deanna M. Langfitt, Carla O'Reilly, Jeoungeun J. Park, Ashley Chabot, Sheng Zhou, Azusa Matsubara, GaHyun Lee, Shengdar Q. Tsai, Shondra M. Pruett-Miller, Lindsay J. Talbot, Jason T. Yustein, Giedre Krenciute, Christopher DeRenzo, Hongbo Chi, and Stephen Gottschalk

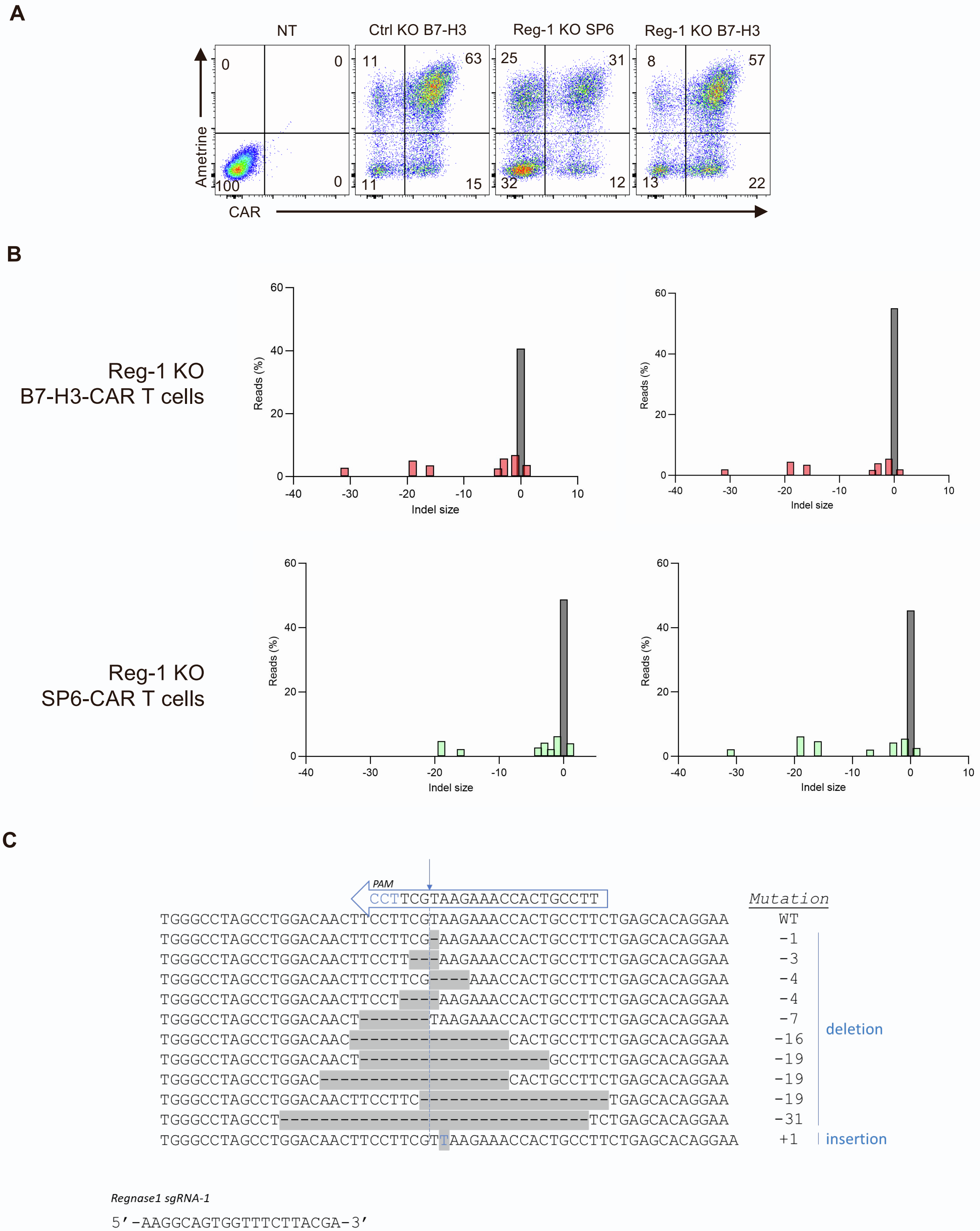

Figure S1: Generation of Reg-1 KO B7-H3-CAR T cells, related to Figure 1. (**A**) Representative flow cytometry plots to evaluate co-transduction efficiency of indicated sgRNA (Ametrine^{+}) and CAR expression in NT, Ctrl KO B7-H3 CAR, Reg-1 KO SP6-CAR, or Reg-1 KO B7-H3-CAR T cells on day 3 post-transduction. (**B**) Top 8 insertion and deletion (Indel) sites in Reg-1 KO SP6 or B7-H3 CAR T cells determined via deep sequencing analysis at the exonic target site of the Reg-1 sgRNA. Top 8 indels compromised 71.1% and 59.3% in Reg-1 KO B7-H3-CAR T cells and 62.3% and 51.2% in controls. Data from 2 independent experiments is shown. (**C**) Indel sequencing results in Reg-1 KO B7-H3-CAR T cells.

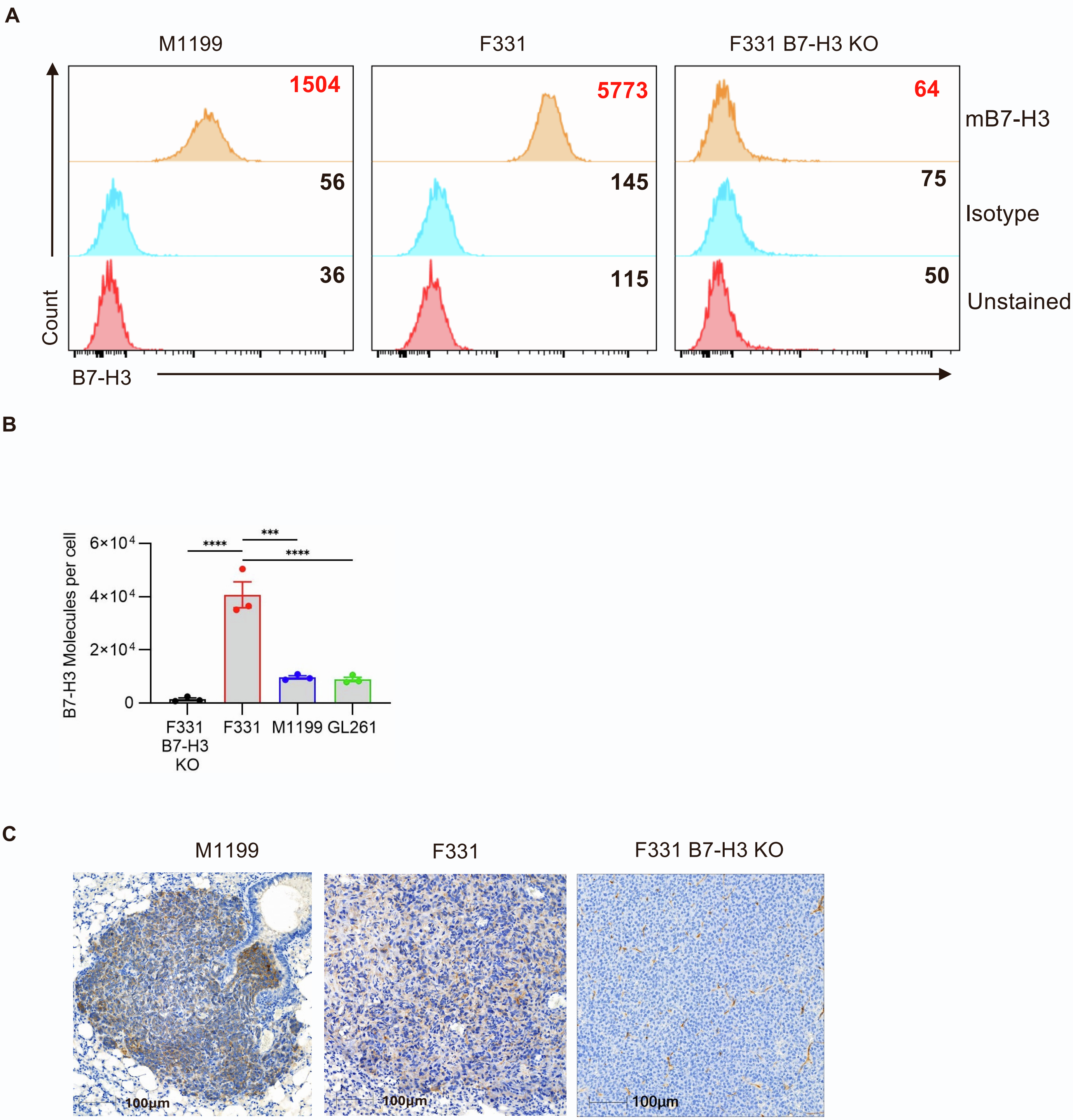

Figure S2: B7-H3 expression on osteosarcoma cell lines, related to Figure 1. (**A**) Representative flow cytometry plot showing B7-H3 expression on M1199 and F331; F331 B7-H3 KO osteosarcoma cell lines was included as a negative control; Red = Unstained, Blue = Isotype control, Orange = B7-H3 staining. Indicated values on upper right corners represents mean fluorescence intensity (MFI). (**B**) B7-H3 molecules per cells representing antigen density evaluated by flow cytometry (Quantum™ MESF kit), n = 3. Data are represented as mean ± SEM. One-way ANOVA with Tukey's test for multiple comparisons; ***:p<0.001, ****:p<0.0001. (**C**) B7-H3 expression was examined by IHC for lung tumors; representative images on day 28 post M1199 or F331 i.v. injection. F331 B7-H3 KO cells were included as a negative control. 3,3'-Diaminobenzidine (DAB) chromogen with hematoxylin counterstain. 20x magnification, 100 µm scale.

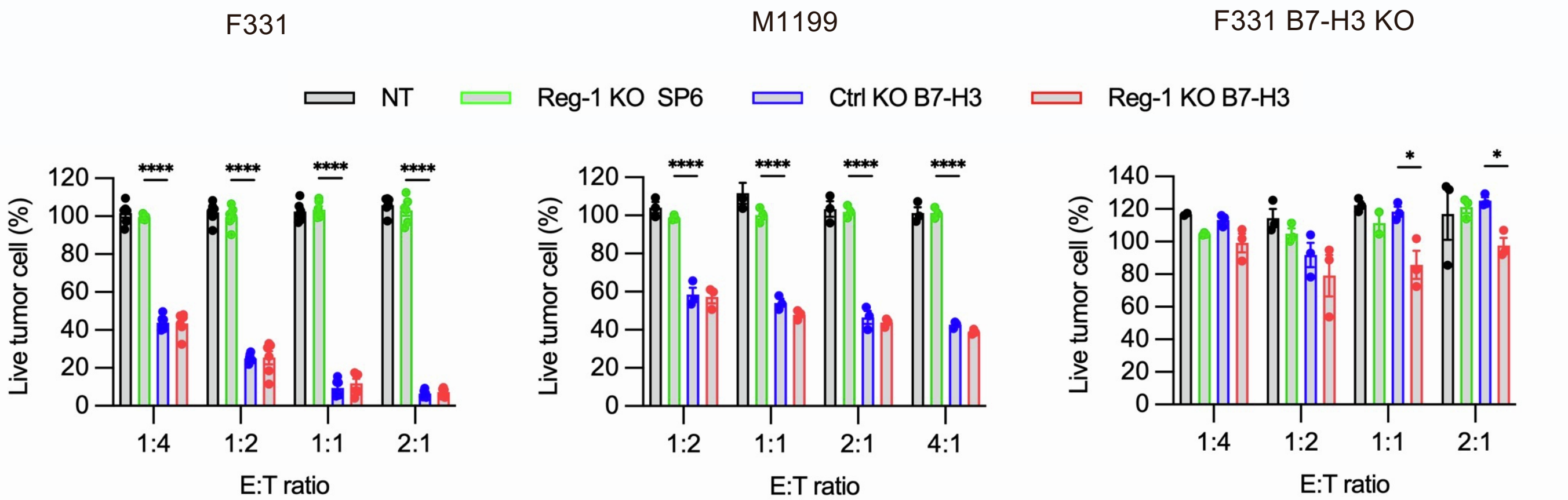

Figure S3: Reg-1 KO B7-H3-CAR T cells demonstrate antigen specific killing *in vitro,* related to Figure 1. 3-(4,5-dimethylthiazol-2-yl)-5-(3-carboxymethoxyphenyl)-2-(4-sulfophenyl)-2H-tetrazolium (MTS) assay with B7-H3-positive (F331, M1199) and B7-H3-negative (F331 B7-H3 KO) cell lines at indicated effector to target (E:T) ratios; n=3. Data are represented as mean ± SEM. Two-way ANOVA with Tukey's test for multiple comparisons; *:p<0.05, ****:p<0.0001.

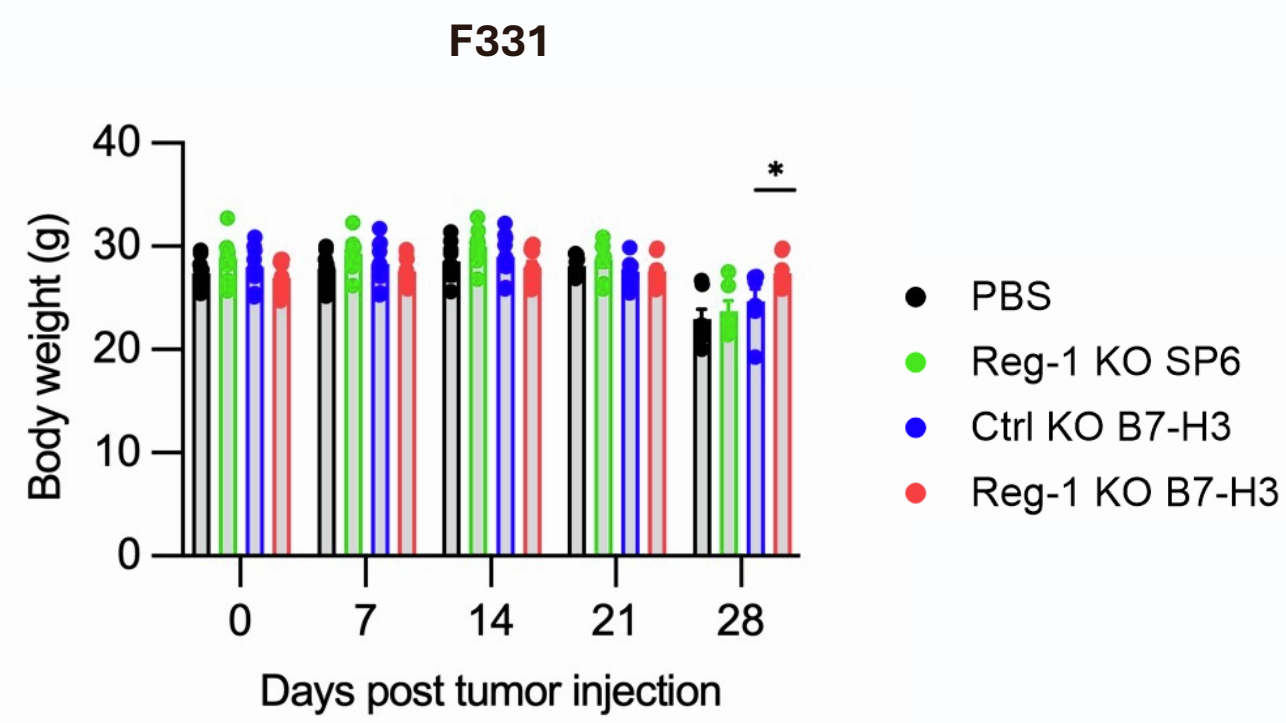

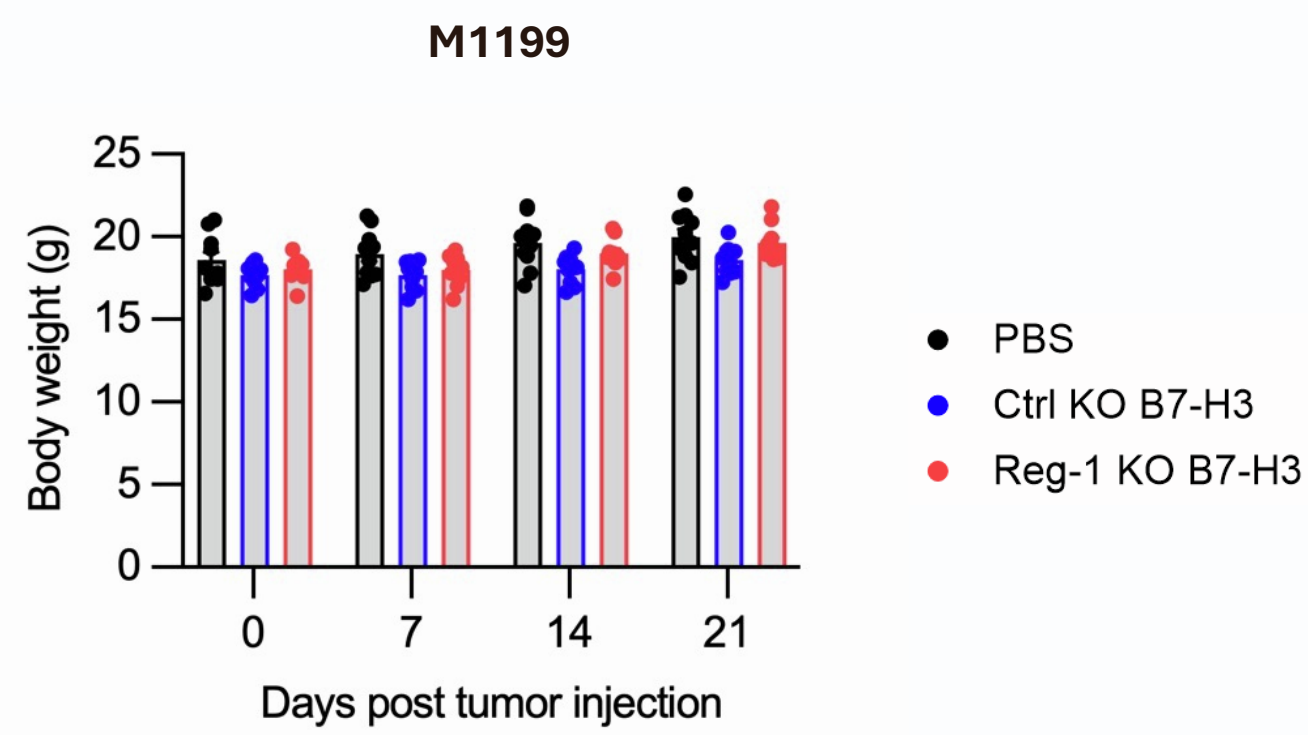

Figure S4: Changes in body weight post-CAR T cell infusion, related to Figure 1. Weekly body weight measurement of F331 and M1199 bearing mice post infusion with Reg-1 KO SP6, Ctrl KO or Reg-1 KO B7-H3-CAR T cells. PBS served as the control. Data are represented as mean ± SEM. Two-way ANOVA with Tukey's test for multiple comparisons; *:$p<0.05$.

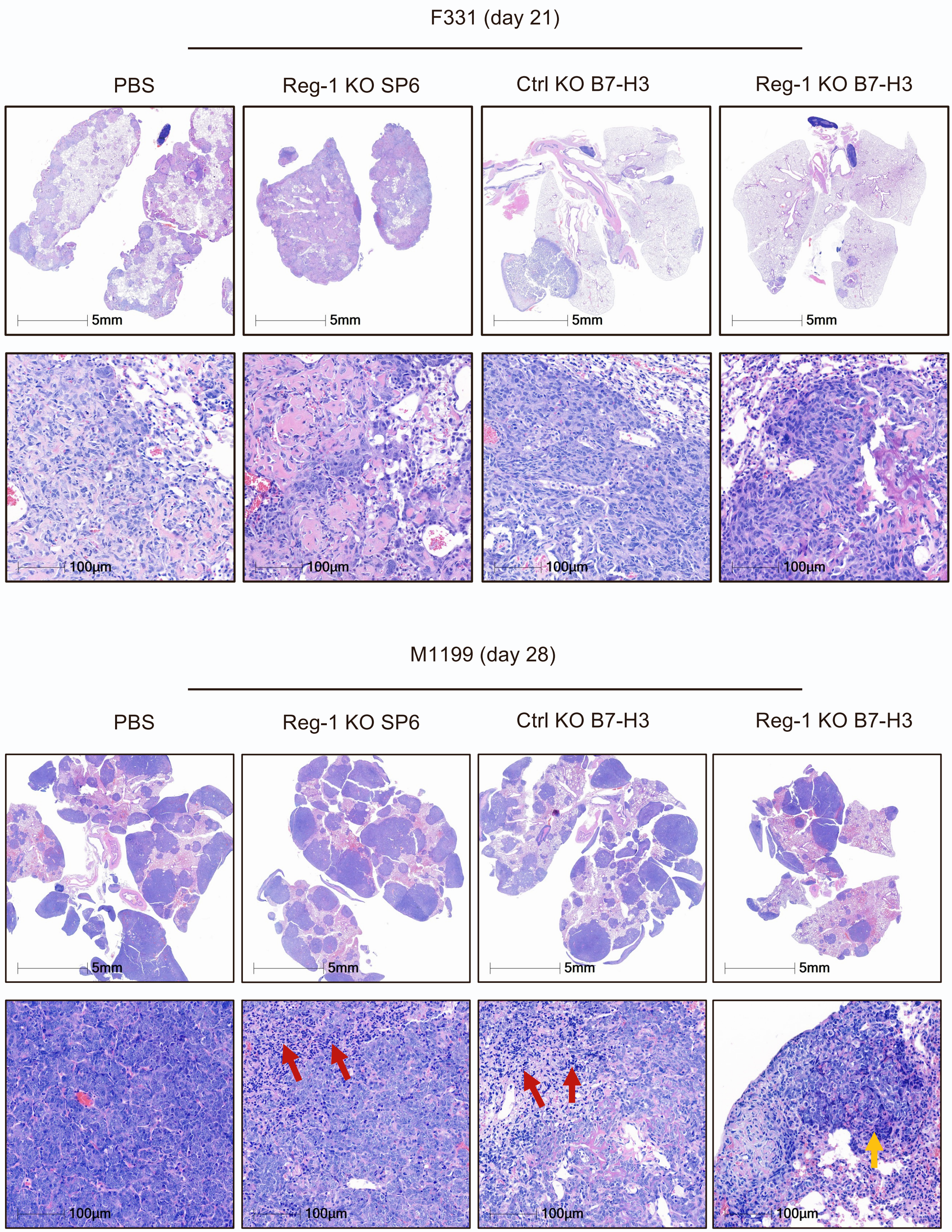

Figure S5: Pathology of osteosarcoma pulmonary tumor post-CAR T cell infusion, related to Figure 1. Hematoxylin and eosin (H&E) staining of lungs from mice on day 21 (F331; upper) and day 28 (M1199; lower) after infusion with Reg-1 KO SP6, Ctrl KO or Reg-1 KO B7-H3-CAR T cells or PBS. Representative images. Top rows: 0.6x magnification, 5 mm scale; bottom rows: 20x magnification, 100 µm scale.

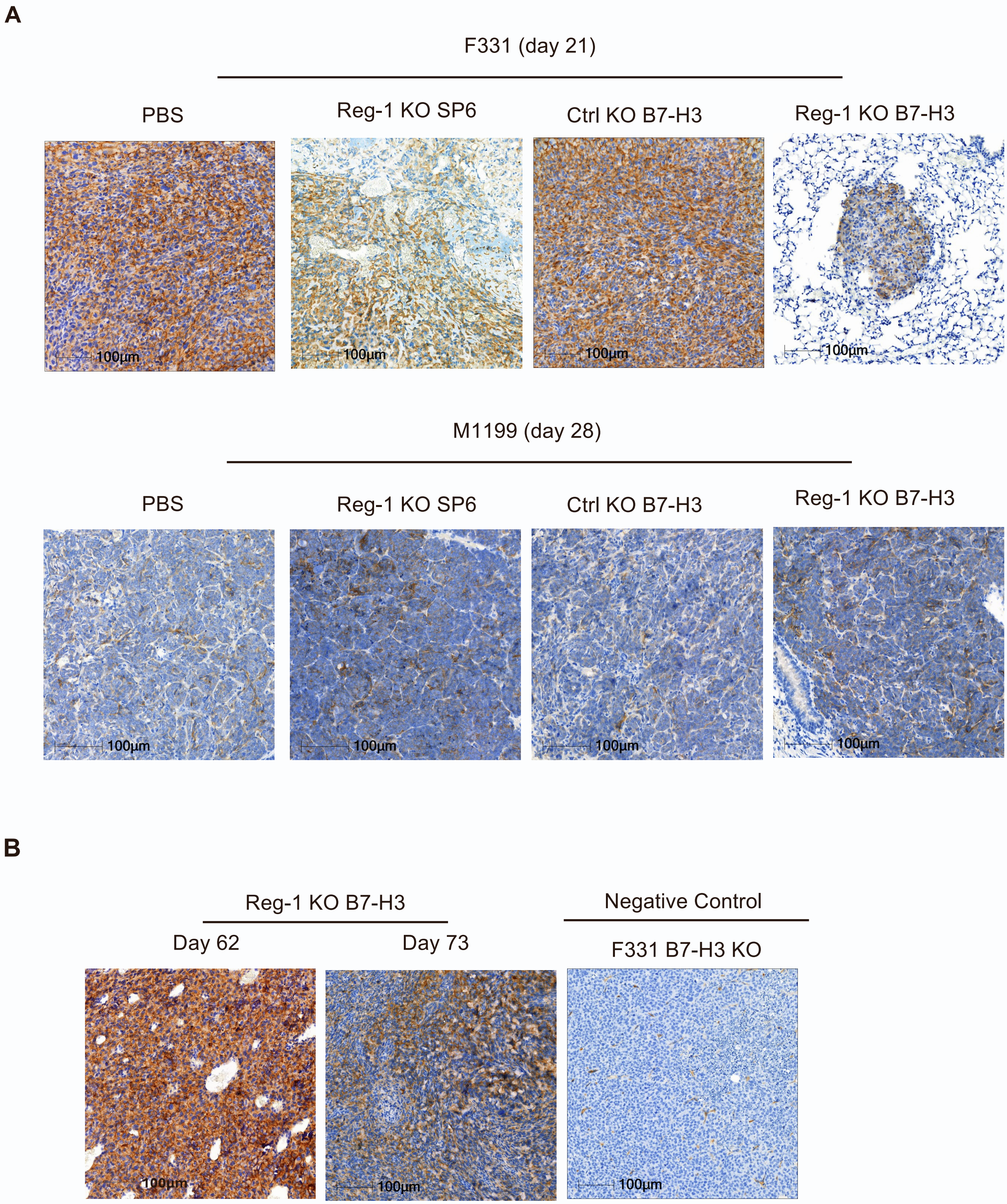

Figure S6: Osteosarcoma continues to express B7-H3 post-CAR T cell infusion, related to Figure 1. (**A**) IHC for B7-H3 expression in lung tumors from F331 and M1199 bearing mice on day 21 (F331; upper) or 28 (M1199; lower) after infusion with Reg-1 KO SP6, Ctrl KO or Reg-1 KO B7-H3-CAR T cells. (**B**) IHC for B7-H3 expression in recurring lungs F331 tumors on days 62 and 73 after Reg-KO B7-H3-CAR T cell infusion. F331 B7-H3 KO lung tumors served as a negative control. 3,3'-Diaminobenzidine (DAB) chromogen with hematoxylin counterstain. 20x magnification, 100 µm scale.

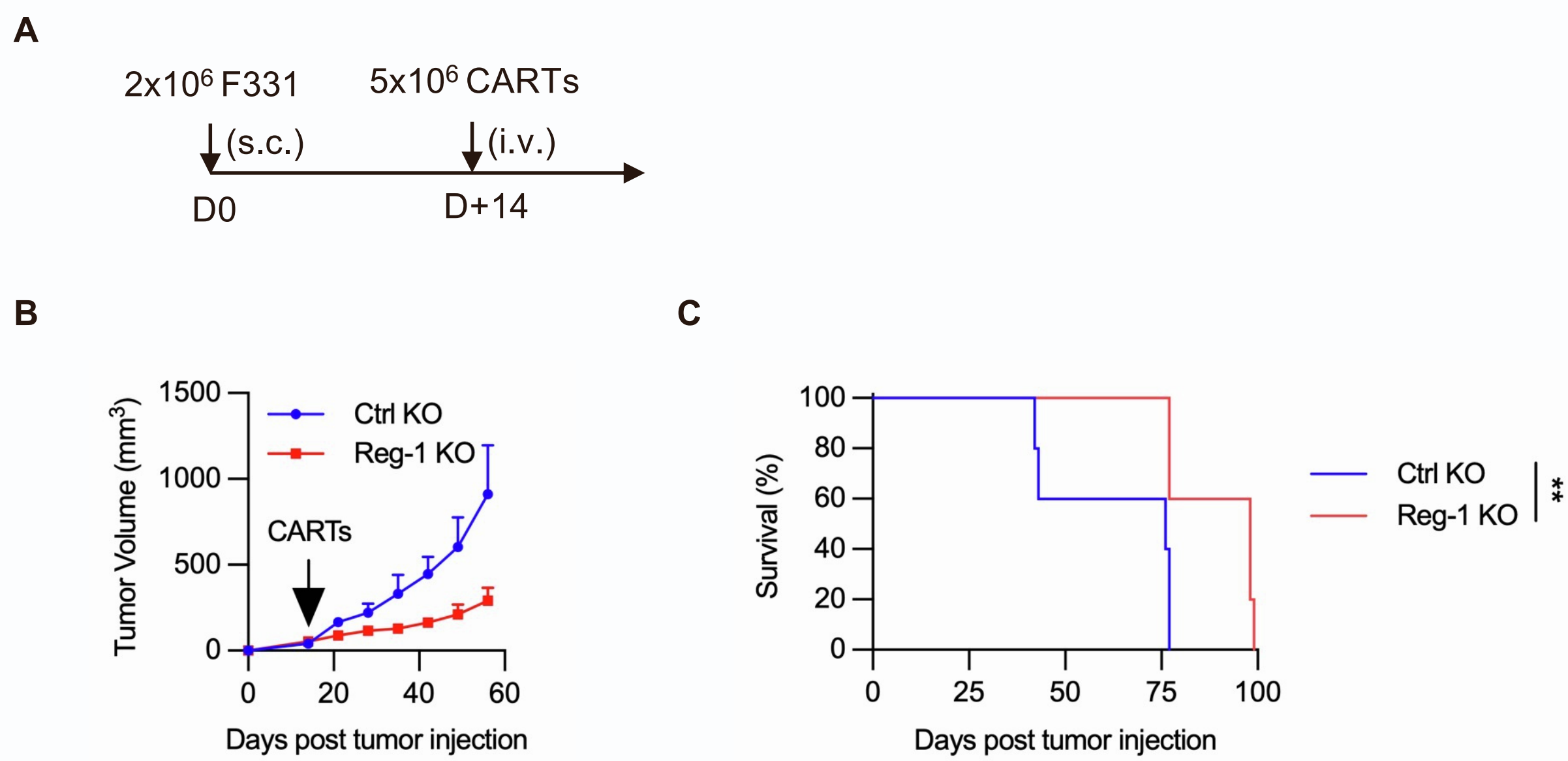

Figure S7: Reg-1 KO improves antitumor activity of B7-H3-CAR T cells in subcutaneous mouse model, related to Figure 1. (**A**) Experimental schematic: C57BL/6 mice were implanted with $2x10^6$ F331 subcutaneously on the right flank and on day 14 received a single i.v. dose of $5x10^6$ Reg-1 KO or Ctrl KO B7-H3-CAR T cell infusion. (**B**) Tumor growth curve (n=5 mice per group). Data are represented as mean ± SEM. (**C**) Kaplan-Meier survival curve, log-rank Mantel-Cox test, n=5 mice per group, **:p<0.01.

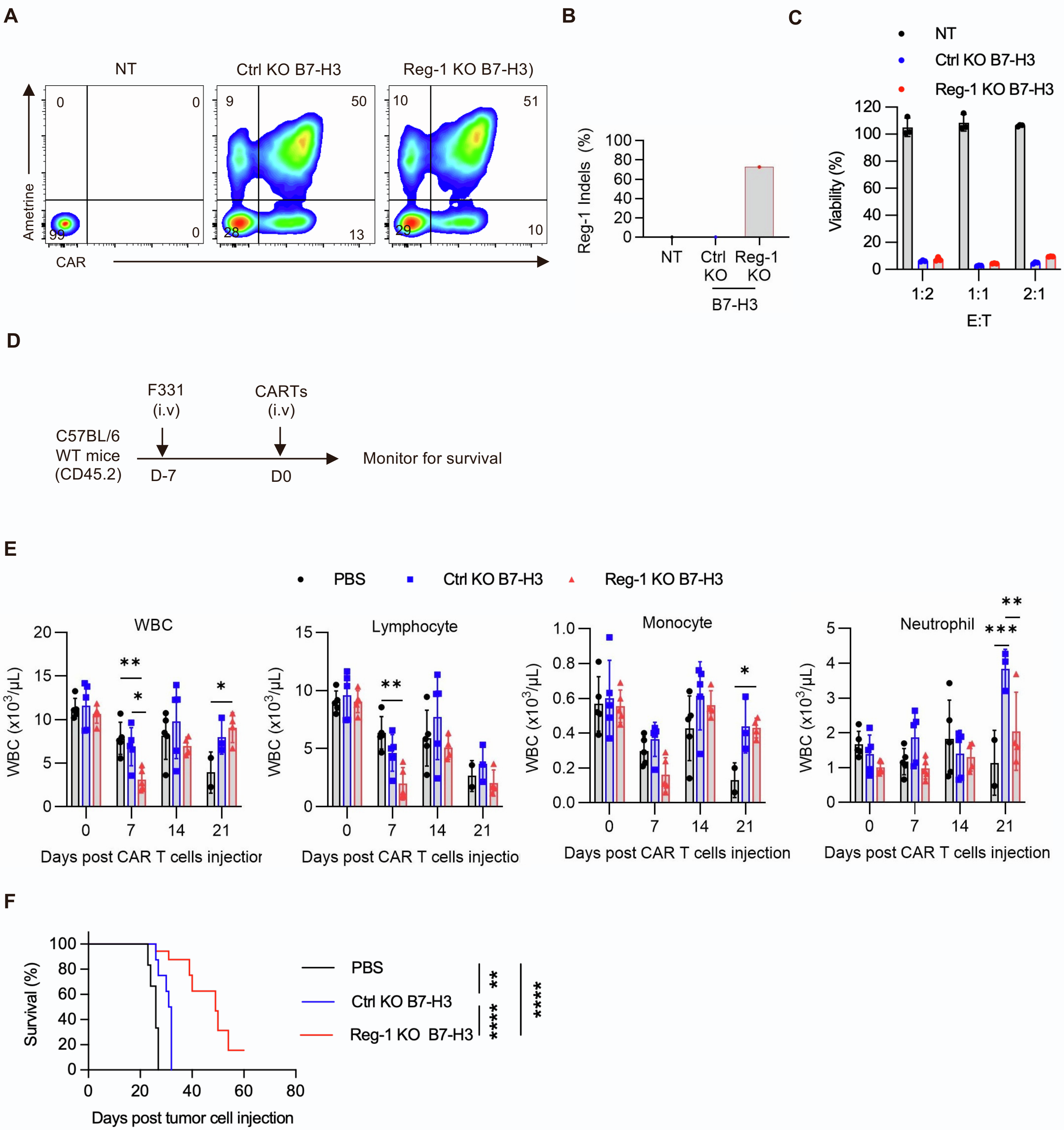

Figure. S8: Reg-1 KO improves the antitumor activity of B7-H3-CAR T cells generated from CD4$^+$/CD8$^+$-selected T cells, related to Figure 1. (**A**) Representative flow cytometry plots to evaluate co-transduction efficiency of indicated sgRNA (Ametrine$^+$) and CAR expression in NT, Ctrl KO B7-H3 CAR, or Reg-1 KO B7-H3-CAR T cells on day 3 post-transduction. (**B**) Insertion and deletion (Indel) analysis of the Reg-1 gene in indicated bulk CAR T cells. (**C**) 3-(4,5-dimethylthiazol-2-yl)-5-(3-carboxymethoxyphenyl)-2-(4-sulfophenyl)-2H-tetrazolium (MTS) assay with B7-H3-positive F331 cell at indicated effector to target (E:T) ratio n=3 (two-way ANOVA with Tukey's test for multiple comparisons). (**D-F**) C57BL/6 mice (CD45.2$^+$) were injected with 1x10^6 F331 cells intravenously (i.v.) and on day 7 received a single i.v. dose of 1.5x10^6 indicated CAR T cell (Cas9$^+$CD45.1$^+$) populations or PBS. (**D**) Experimental schematic, (**E**) Complete blood count analysis on days 0, 7, 14 and 21 post-CAR T cell infusion (n=2-5 mice per group), Data are represented as mean ± SEM. (**F**) Log-rank Mantel-Cox test, n=7-8 mice per group; two-way ANOVA with Tukey's test for multiple comparisons. *:p<0.05, **:p<0.01, ***:p<0.001, ****:p<0.0001.

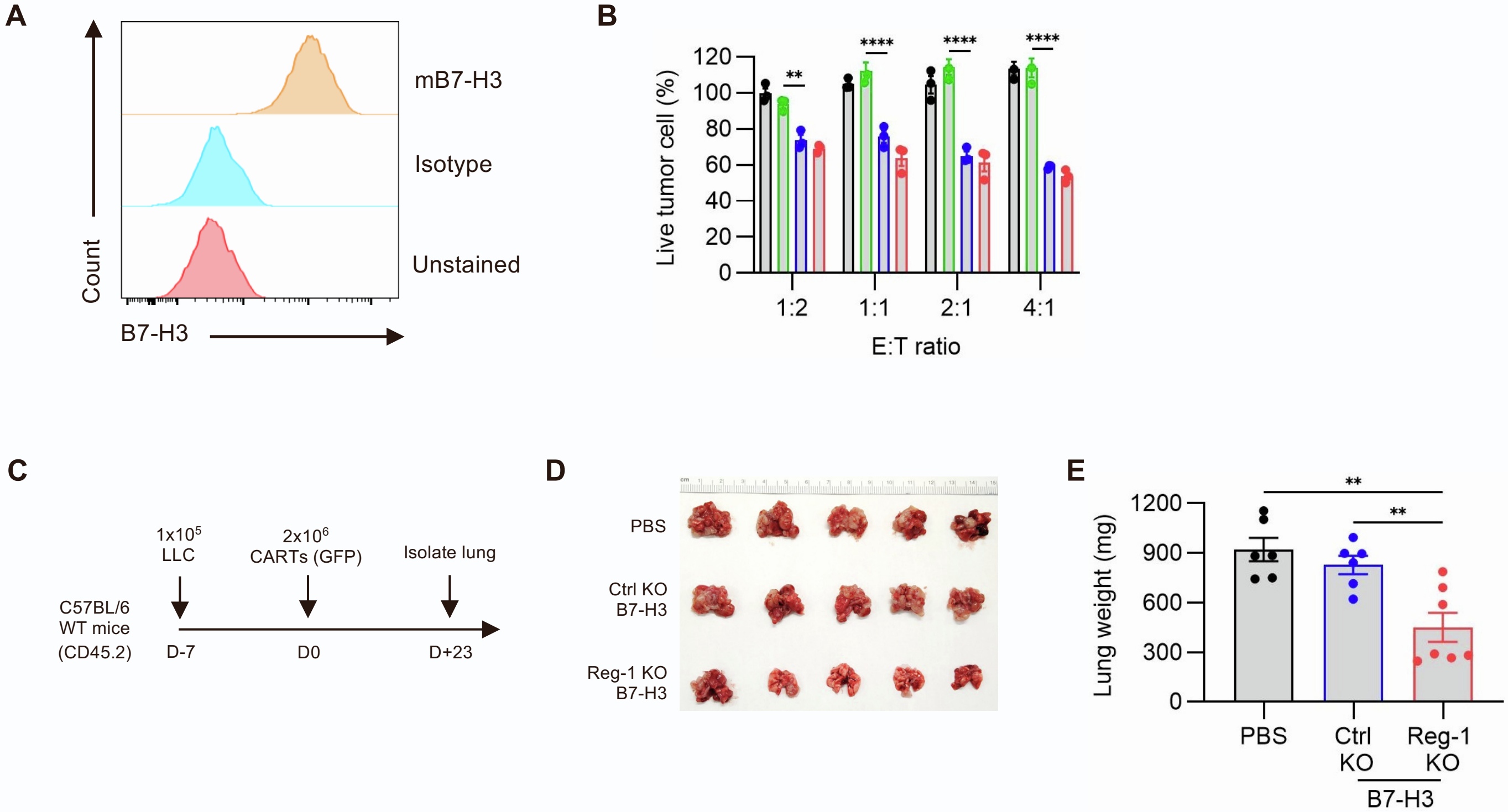

Figure. S9: Reg-1 KO improves B7-H3-CAR T cell antitumor activity in LLC model, related to Figure 1

(**A**) Representative flow cytometry plot showing B7-H3 expression on LLC cell line; Red = Unstained, Blue = Isotype control, Orange = B7-H3 staining. (**B**) 3-(4,5-dimethylthiazol-2-yl)-5-(3-carboxymethoxyphenyl)-2-(4-sulfophenyl)-2H-tetrazolium (MTS) assay with LLC cell line at indicated effector to target (E:T) ratios; n=3. (**C-E**) C57BL/6 mice (CD45.2+) were injected with 1x10^5 LLC cells intravenously (i.v.) and on day 7 received a single i.v. dose of 2 x10^6 indicated CAR T cell (Cas9$^+$GFP$^+$) populations or PBS. (C) Experimental schematic, (D) Representative macroscopic images of lungs (n=5) and (E) lung weight (n= 6-7) on day 23 post-CAR T cell infusion. Data are represented as mean ± SEM. Two-way ANOVA with Tukey's test for multiple comparisons; **:p<0.001, ****:p<0.0001.

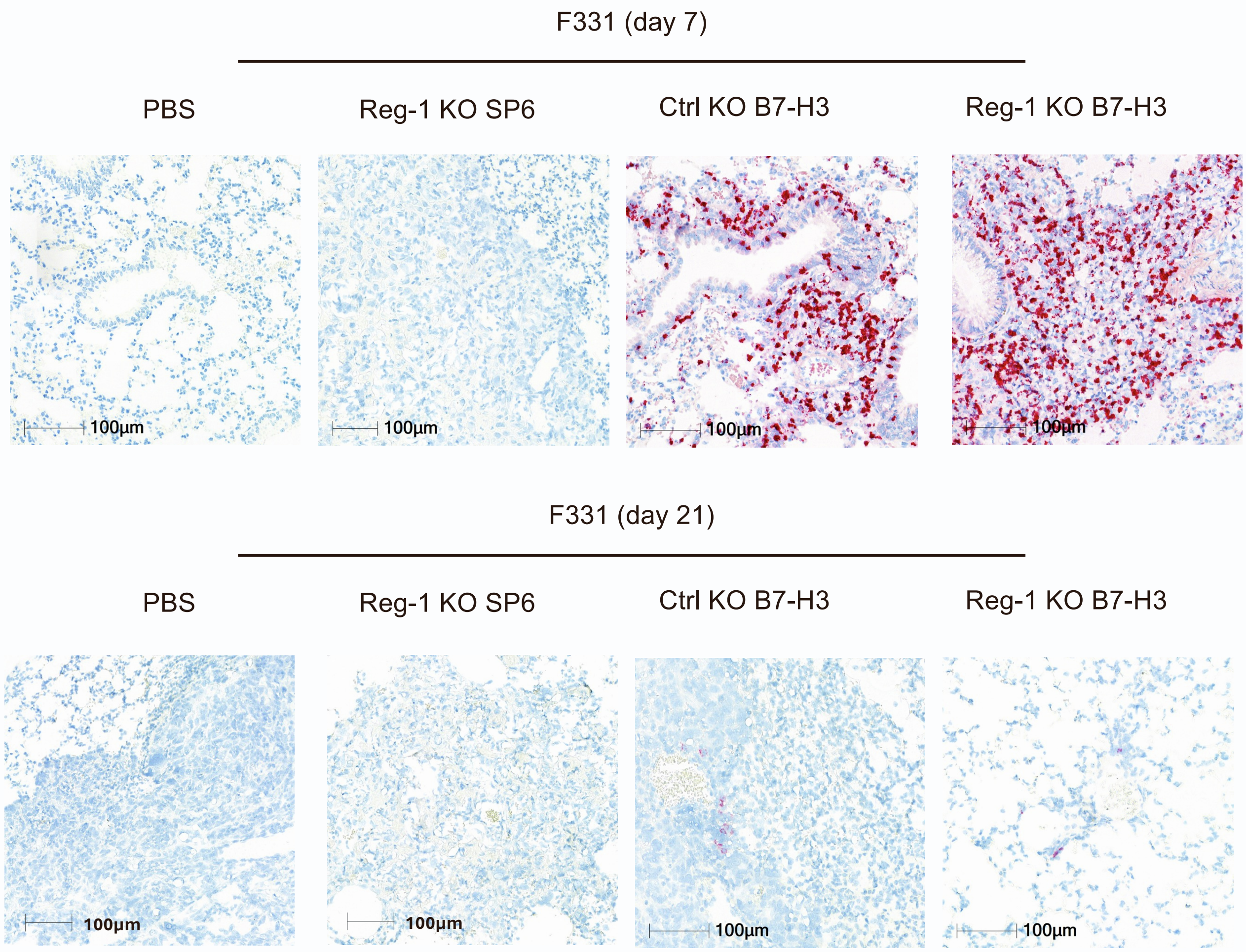

Figure S10: B7-H3-CAR T cells traffic to pulmonary metastases post-infusion, related to Figure 2. Detection of B7-H3-CAR T cells with an *in situ* hybridization (ISH) probe specific for the B7-H3-CAR in the F331 model on day 7 (top) and day 21 (bottom) after infusion with Reg-1 KO SP6, Ctrl KO or Reg-1 KO B7-H3-CAR T cells. CAR T cell immunolabeled with red chromogen and hematoxylin counterstain. The hole in the Ctrl-KO slide on day 7 is the lumen of a terminal bronchiole. 20x magnification, 100 µm scale.

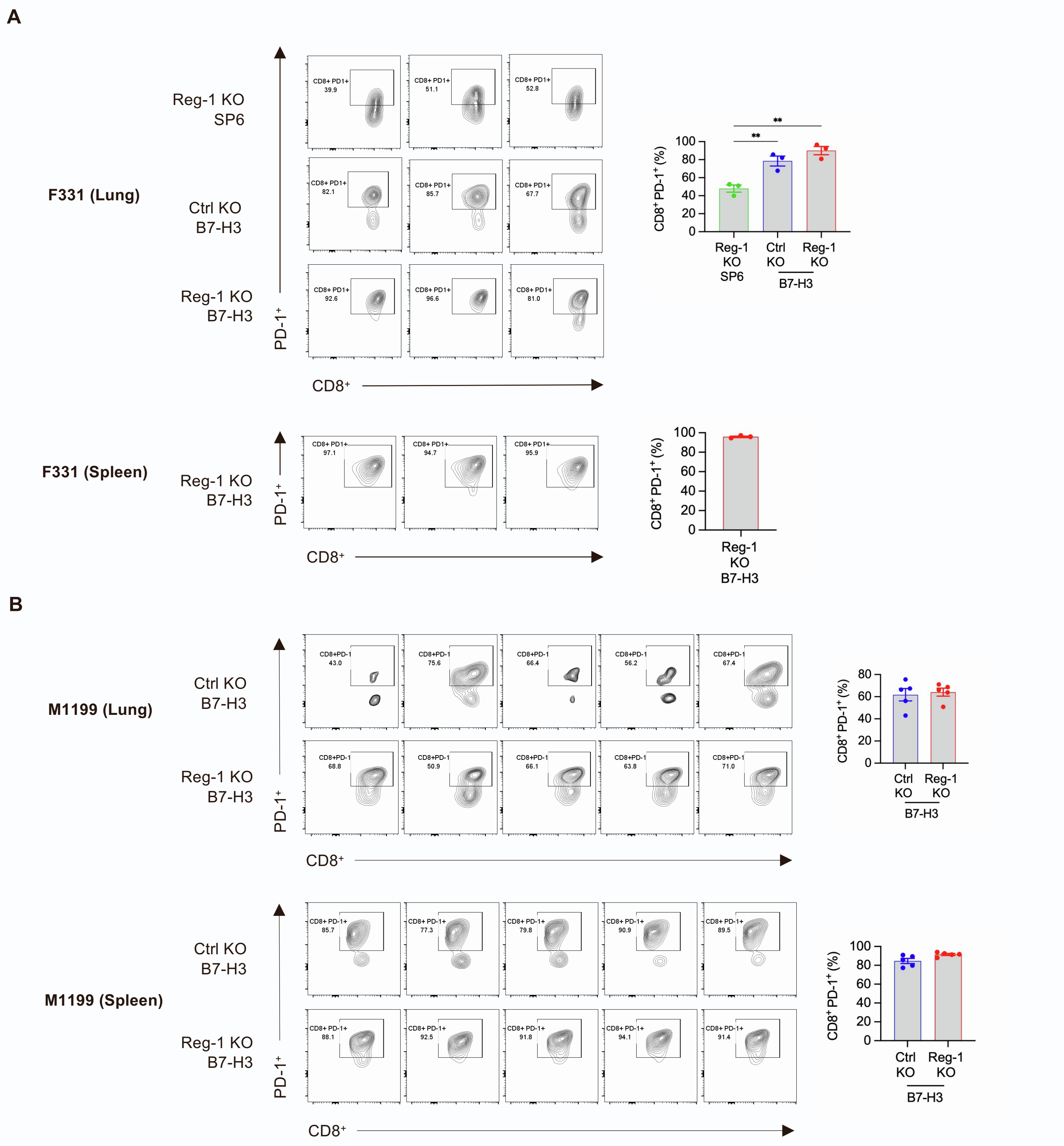

Figure S11: Reg-1 KO did not alter PD-1 expression on B7-H3-CAR T cells, related to Figure 2. (**A**) C57BL/6 mice were injected with 1x10^6 F331 (i.v.) and on day 7 received a single i.v. dose of 1x10^6 Reg-1 KO SP6, Ctrl KO or Reg-1 KO B7-H3-CAR T cells. PBS-treated mice served as control. Flow cytometry plots and quantification of PD-1 expression on CD8$^+$ CAR T cells in the lungs and spleens on day 7 post CAR T cell infusion; n=3 mice per group (there are not enough CAR T cells in the spleens of Reg-1 KO SP6 and Ctrl KO B7-H3-CAR T cell-treated mice to make a comparison). (**B**) C57BL/6 mice were implanted with 2x10^5 M1199 (i.v.) and on day 7 received a single i.v. dose of 2x10^6 Ctrl KO or Reg-1 KO B7-H3-CAR T cells. PBS-treated mice served as control. Flow cytometry plots and quantification of PD-1 expression on CD8$^+$ B7-H3-CAR T cells in the lungs and spleens on day 7 post CAR T cell infusion, n=5 mice per group. Data are represented as mean ± SEM. One-way ANOVA, **:p<0.01, Turkey's multiple comparison test.

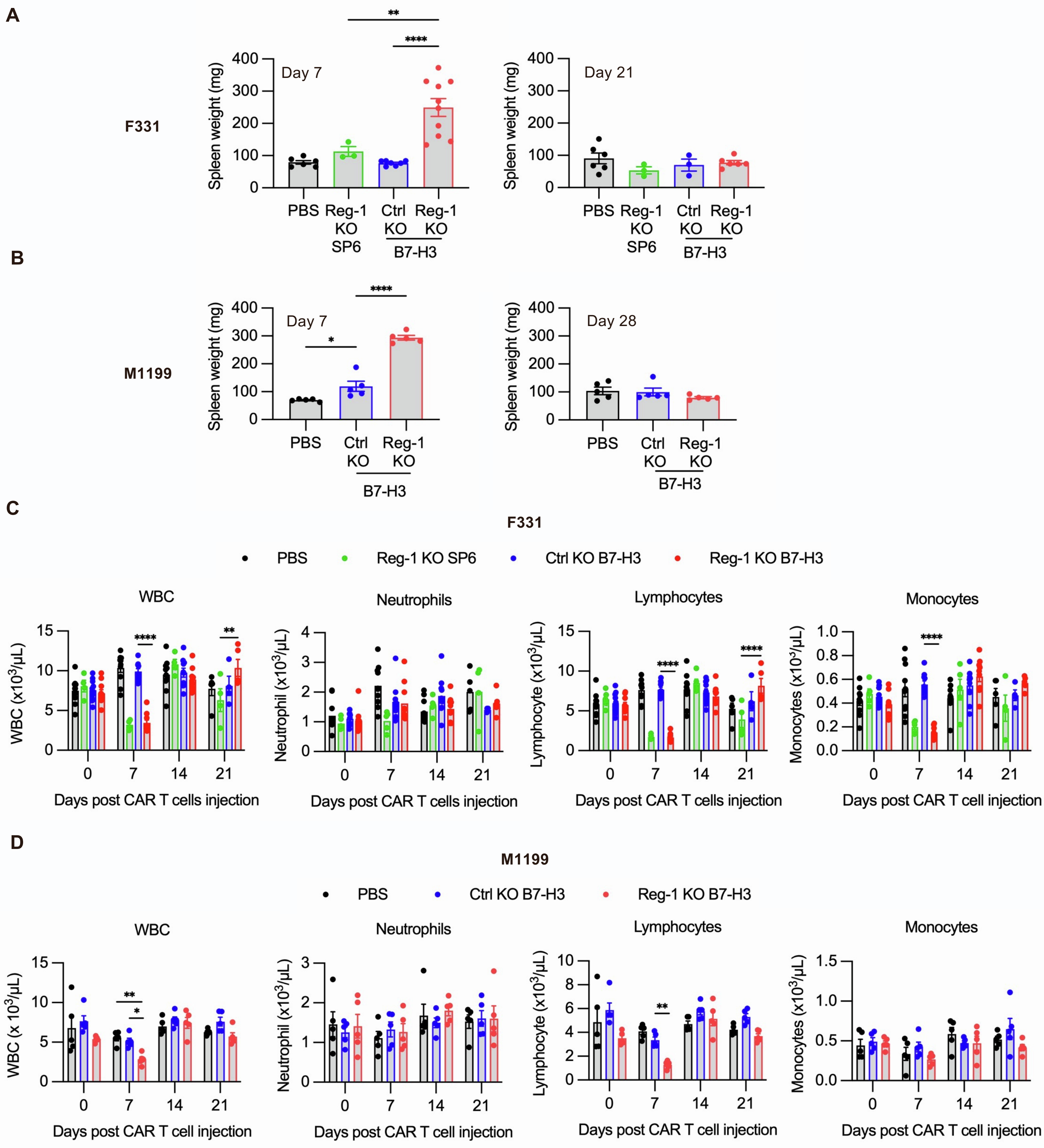

Figure S12: Reg-1 KO B7-H3-CAR T cells induced transient splenomegaly, lympho- and monocytopenia on day 7 post injection, related to Figure 2. (**A**) Mice were injected with F331 and received indicated CAR T cells as described for Fig. S11A. Spleens weight is shown for days 7 and 21 post CAR T cell infusion (n=3-10 mice per group). (**B**) Mice were injected with M1199 cells and received indicated CAR T cells as described for **Fig. S11B**. Weight of spleens on days 7 and 28 post CAR T cell infusion (n=5 mice per group). (**C,D**) Complete blood count analysis on days 0, 7, 14 and 21 post-CAR T cell infusion in the (**C**) F331 or (**D**) M1199 model (n=5-10 mice per group). Data are represented as mean ± SEM. One way or Two-way ANOVA with Tukey's test for multiple comparisons; *: p<0.05; **:p<0.01; ****:p<0.0001.

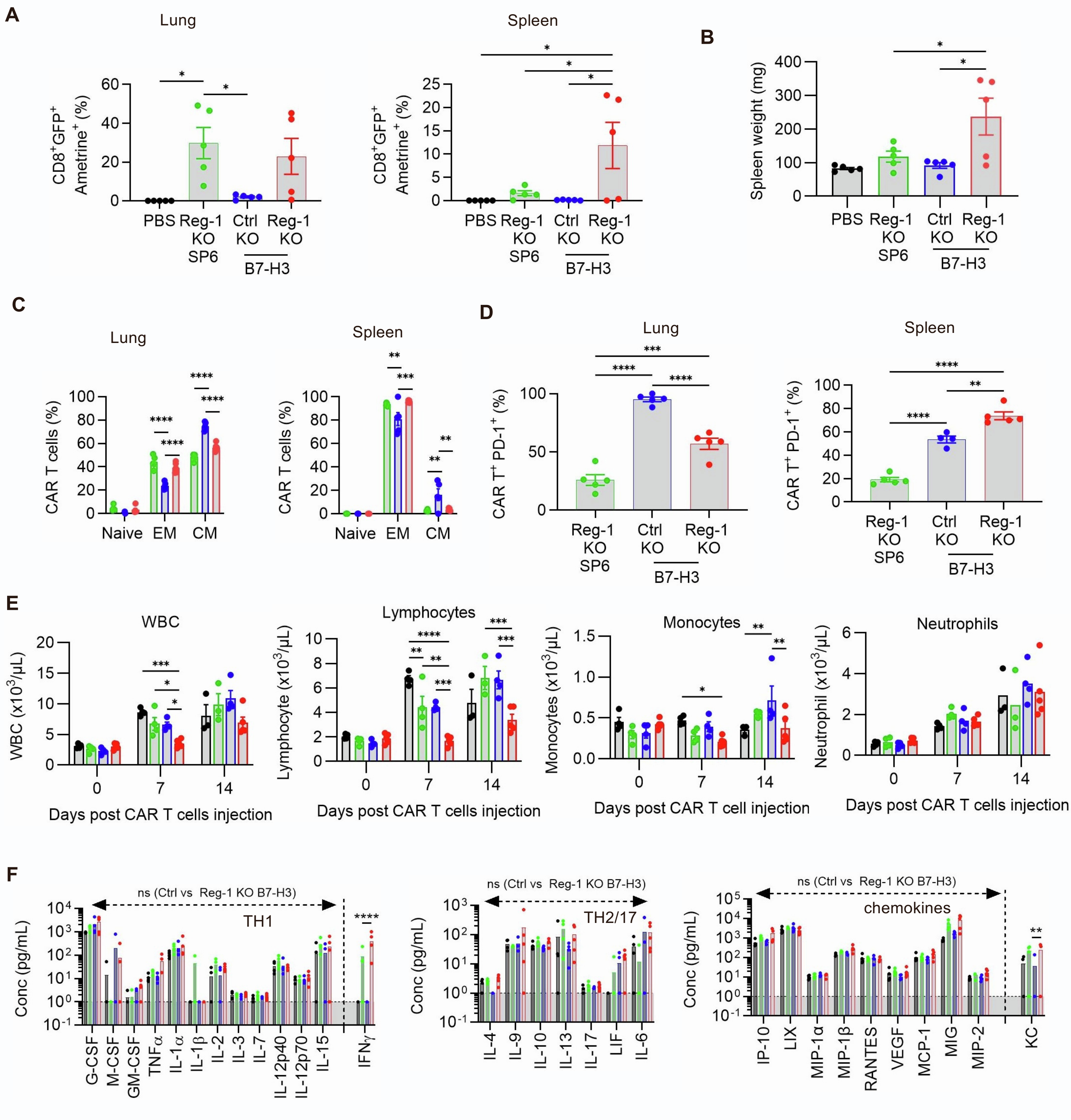

Figure S13: Reg-1 KO enhances B7-H3-CAR T-cell expansion, induce splenomegaly, transient lymphopenia and elevated serum IFNγ level in LLC model, related to Figure 2. C57BL/6 mice were injected with $5x10^5$ LLC (i.v.) and on day 7 received a single i.v. dose of $2x10^6$ CAR T-cell population or PBS. (**A**) Quantification of lungs- and spleens-infiltrating $CD8^+GFP^+Ametrine^+$ T cells on day 7. (**B**) Spleen weight on day 7. (**C**) Phenotyping of lungs- and spleens-infiltrating CAR T-cell differentiation states, classified as naïve ($CD62L^+CD44^-$), effector memory (EM; $CD62L^-CD44^+$) and central memory (CM; $CD62L^+CD44^+$) subsets on day 7. (**D**) PD-1 expression on lungs- and spleens-infiltrating CAR T-cell on day 7. (**E**) Complete blood count analysis on days 0, 7, and 14 post-CAR T cell infusion (**F**) Multiplex analysis of cytokines in the serum on day 7 post treatment with indicated CAR T cells or PBS n=5. Data are represented as mean ± SEM. One or Two-way ANOVA with Tukey's test for multiple comparisons). *:p<0.05; **:p<0.01; ***:p<0.001; ****:p<0.0001.

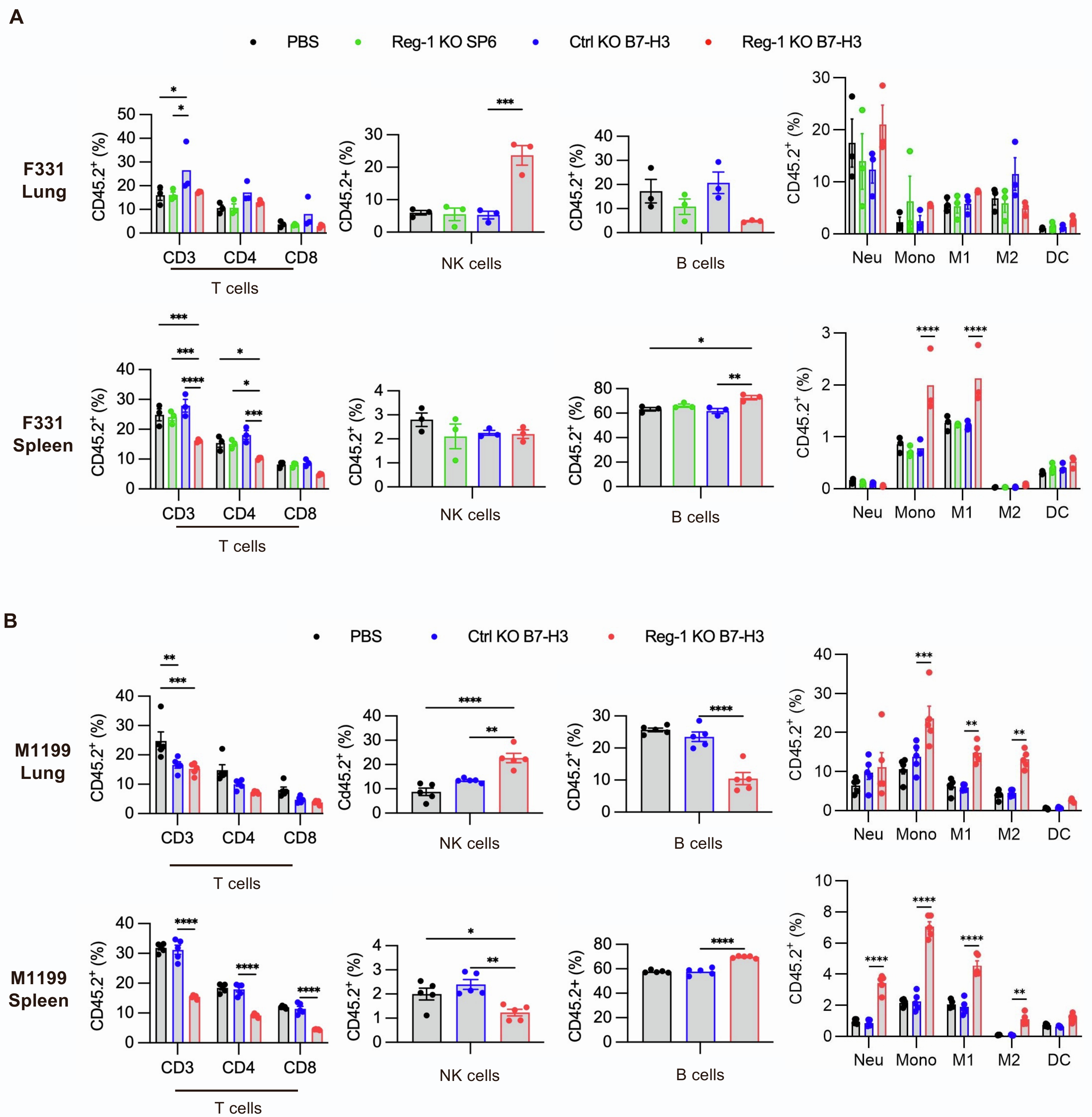

Figure S14: Reg-1 KO B7-H3-CAR T cells induce infiltration of NK cells, related to Figure 2. (**A, B**) Mice were injected with (**A**) F331 or (**B**) M1199 cells, followed by infusion with indicated CAR T cells as described in Fig. S11. (**A**) Percentages of T, NK, B and myeloid cell subsets among CD45.2^{+} viable cells in the lungs and spleens on day 7 after CAR T cell infusion (n=3 mice per group). (**B**) Percentages of T, NK, B and myeloid cell subsets among CD45.2^{+} viable cells in the lungs and spleens on day 7 after CAR T cell infusion (n=5 mice per group). NK: natural killer cell, DC: Dendritic cell. Data are represented as mean ± SEM. Two-way ANOVA with Tukey's test for multiple comparisons or unpaired student's *t*-test: *:p<0.05, **:p<0.01, ***:p<0.001, ****:p<0.0001. Neu: Neutrophil, Mono: Monocyte, M1: M1-like macrophages, M2: M2-like macrophages, DC: Dendric cell

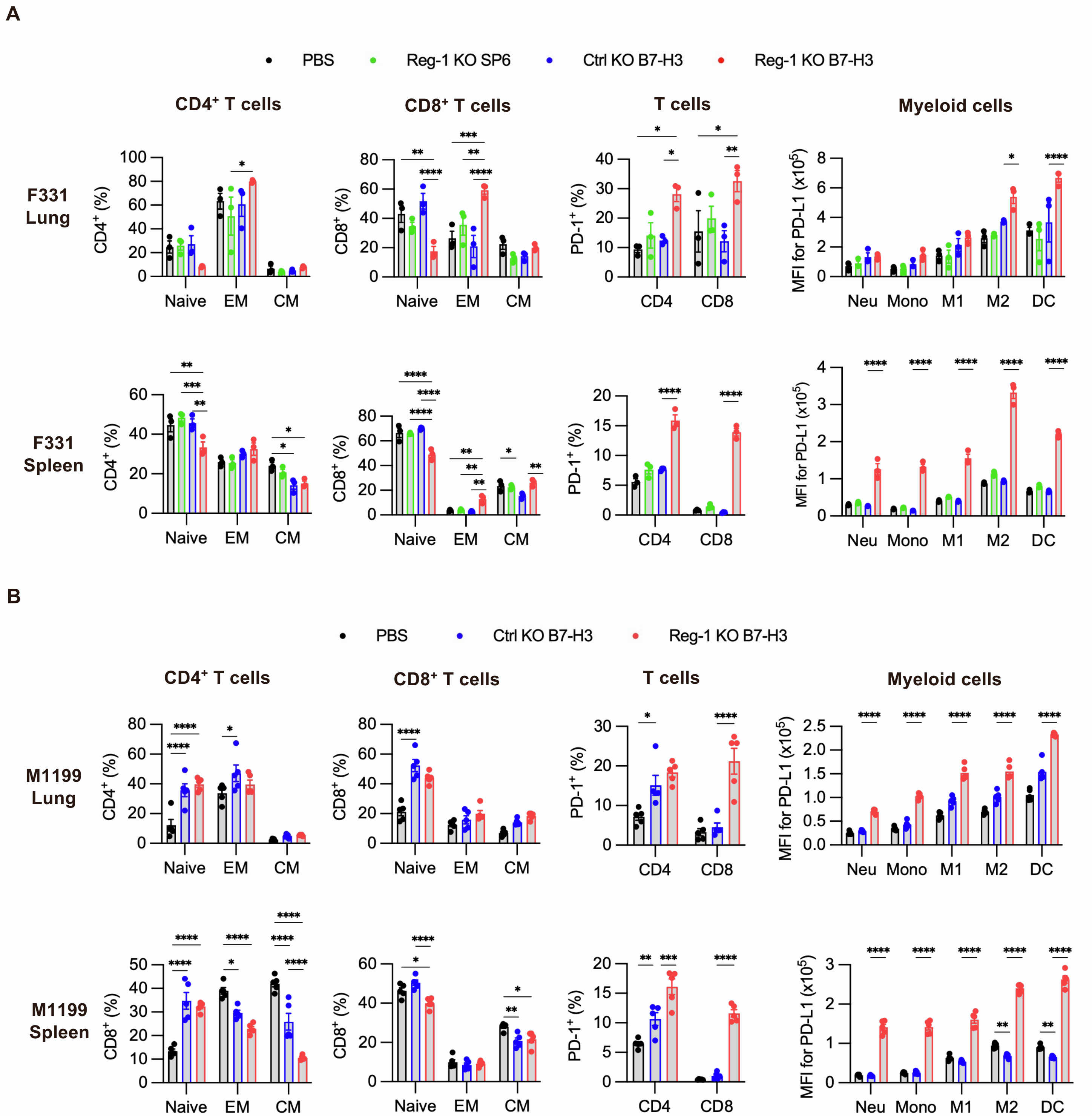

Figure S15: Reg-1 KO B7-H3-CAR T cells reshape the endogenous immune cell landscape in F331 and M1199 models, related to Figure 2. (**A, B**) Mice were injected with (**A**) F331 (n=3 mice per group) or (**B**) M1199 (n=5 mice per group) cells, followed by infusion with indicated CAR T cells as described in Fig. S11. From left to right: Percentages of endogenous naïve ($CD62L^+CD44^-$), effector memory (EM; $CD62L^-CD44^+$) and central memory (CM; $CD62L^+CD44^+$) populations among $CD4^+$ and $CD8^+$ T cells. Percentages of PD-1^+ cells among endogenous $CD4^+$ and $CD8^+$ T cells. Percentages of PD-L1 expression in indicated myeloid cell populations. Data are represented as mean ± SEM. Two-way ANOVA with Tukey's test for multiple comparisons; *:p<0.05, **:p<0.01, ***:p<0.001, ****:p<0.0001. Neu: Neutrophil, Mono: Monocyte, M1: M1-like macrophages, M2: M2-like macrophages, DC: Dendric cell

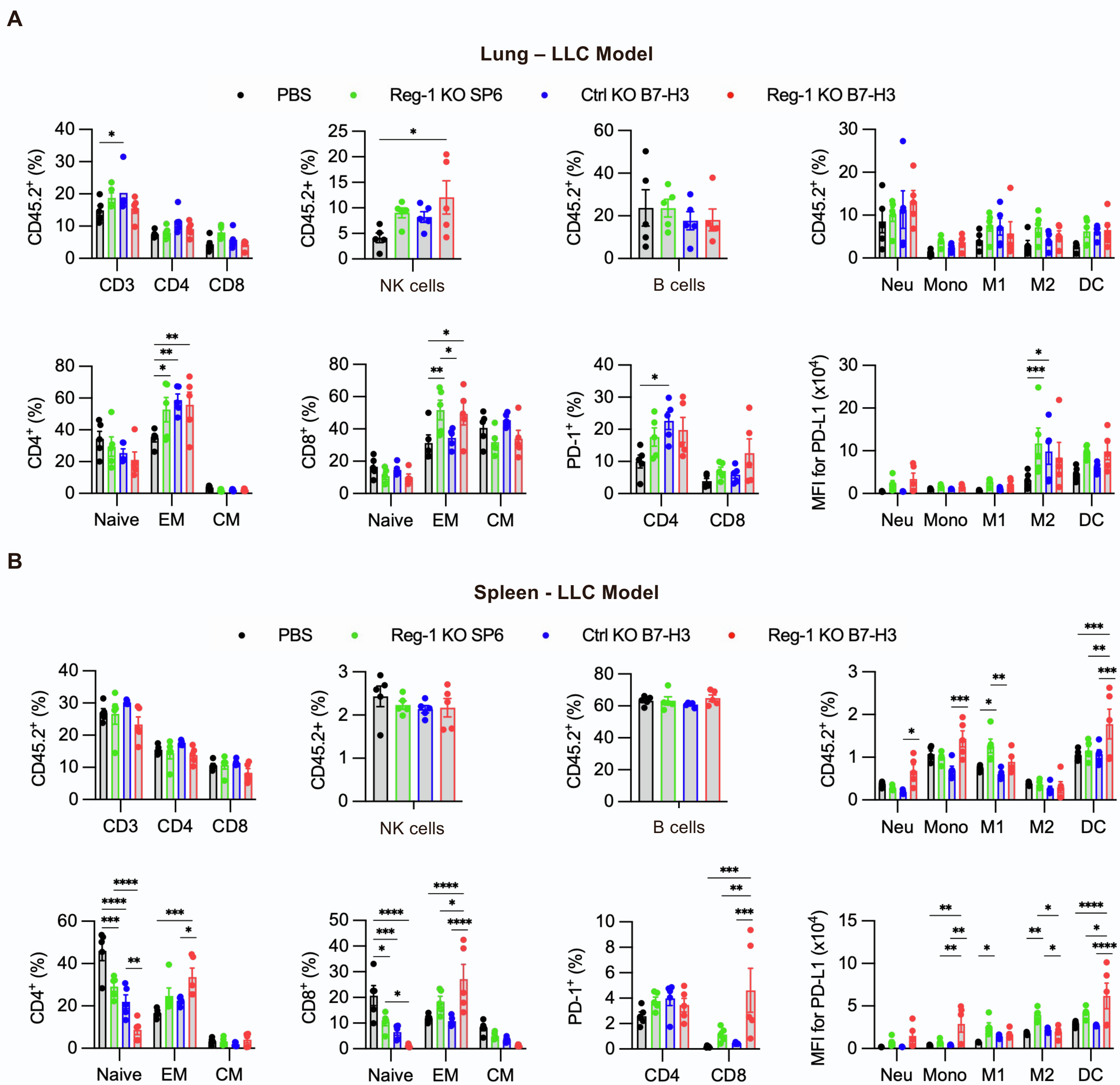

Figure S16: Reg-1 KO B7-H3-CAR T cells reshape the endogenous immune cell landscape in LLC model, related to Figure 2. For experimental details, see Fig. S13. Endogenous immune cells were analyzed in the (**A**) lung and (**B**) spleen on day 7 post CAR T-cell injection (n=5 mice per group). Top row – from left to right: Percentages of T, NK, B and myeloid cell subsets among $CD45.2^+$ viable cells. Botton row - from left to right: Percentages of endogenous naïve ($CD62L^+CD44^-$), effector memory (EM; $CD62L^-CD44^+$) and central memory (CM; $CD62L^+CD44^+$) populations among $CD4^+$ and $CD8^+$ T cells. Percentages of $PD\text{-}1^+$ cells among endogenous $CD4^+$ and $CD8^+$ T cells. Percentages of PD-L1 expression in indicated myeloid cell populations. Data are represented as mean ± SEM. Two-way ANOVA with Tukey's test for multiple comparisons; *:p<0.05, **:p<0.01, ***:p<0.001, ****:p<0.0001. Neu: Neutrophil, Mono: Monocyte, M1: M1-like macrophages, M2: M2-like macrophages, DC: Dendritic cell

A

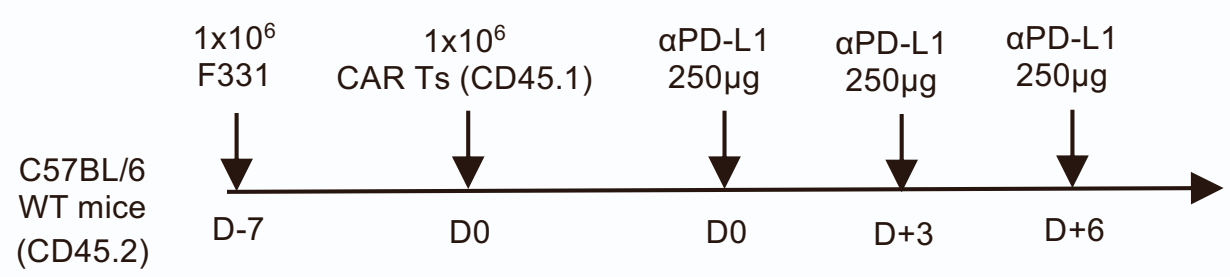

B

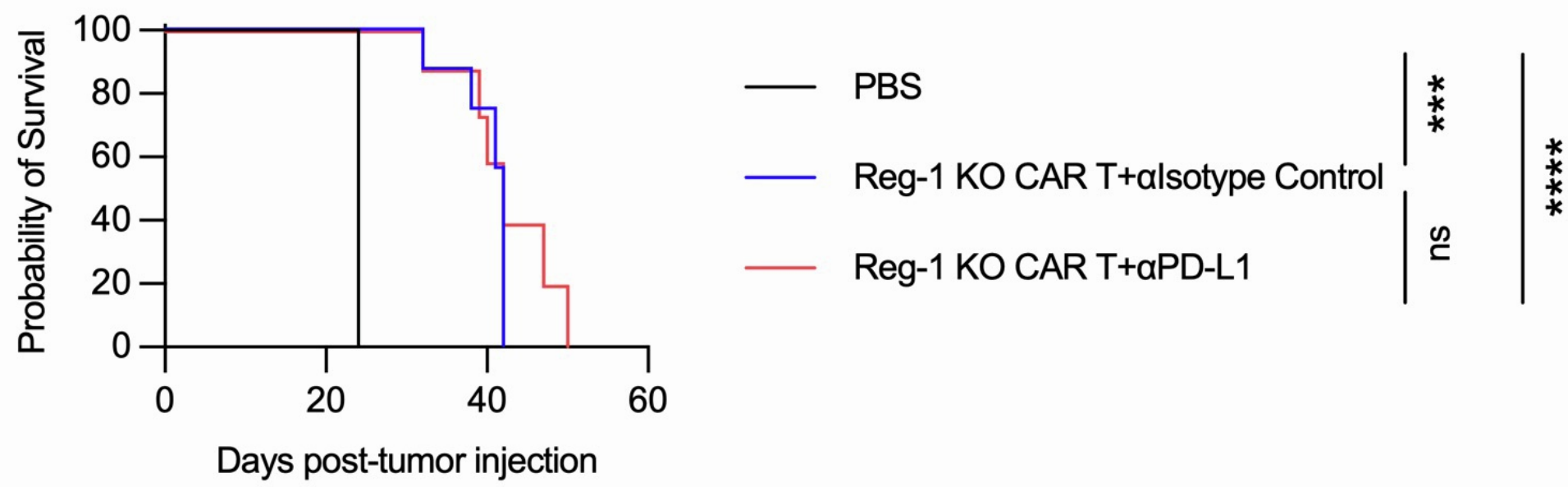

Figure S17: PD-L1 blockade does not improve Reg-1 KO B7-H3-CAR T cells antitumor activity related to Figure 2. (**A**) Experimental schematic. (**B**) Kaplan-Meier survival curve for F331 tumor-bearing mice that received Reg-KO B7-H3-CAR T cells with or without PD-L1 antibody as indicated (n=5 mice per group). Log-rank Mantel-Cox test; ****: $p<0.0001$.

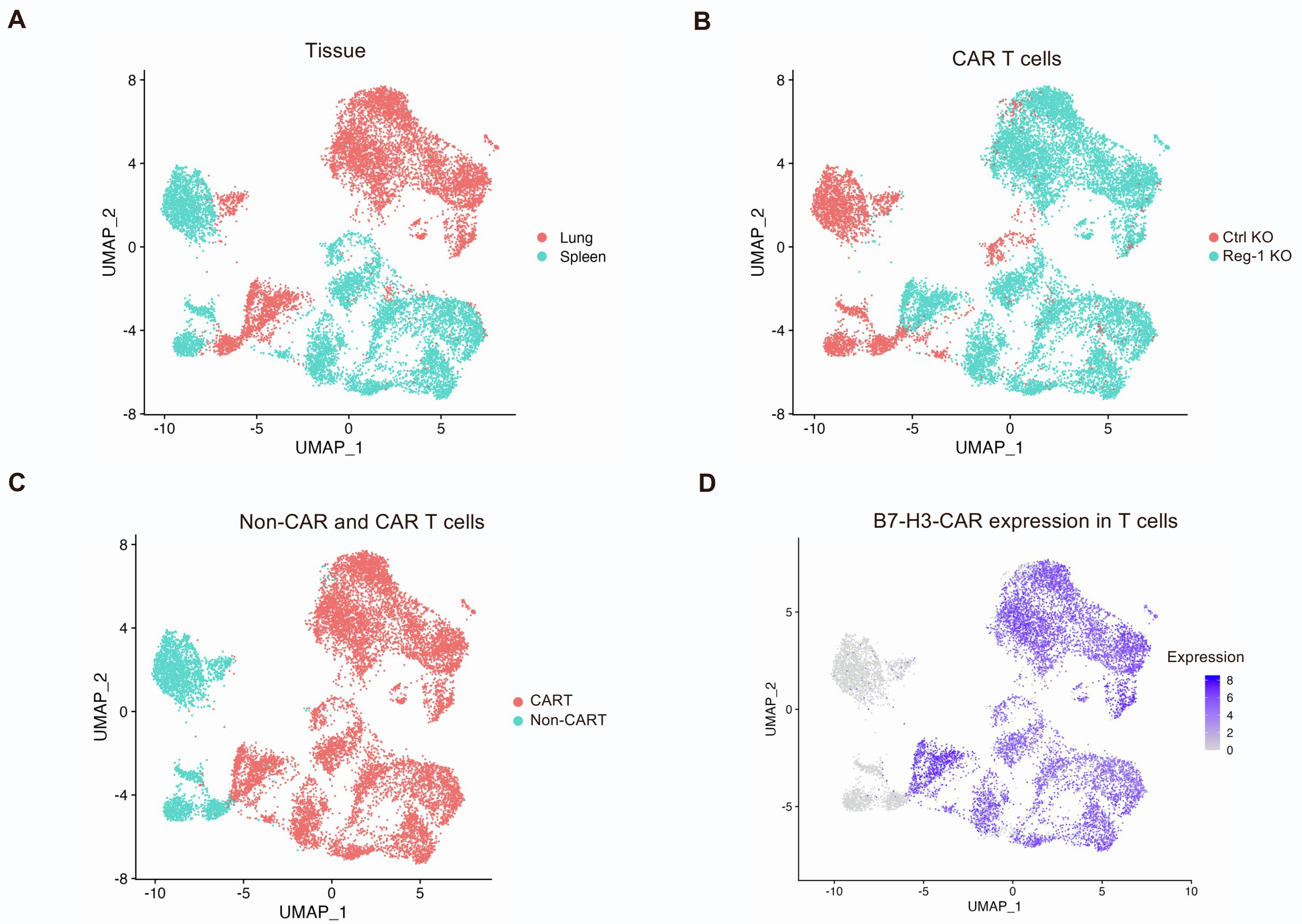

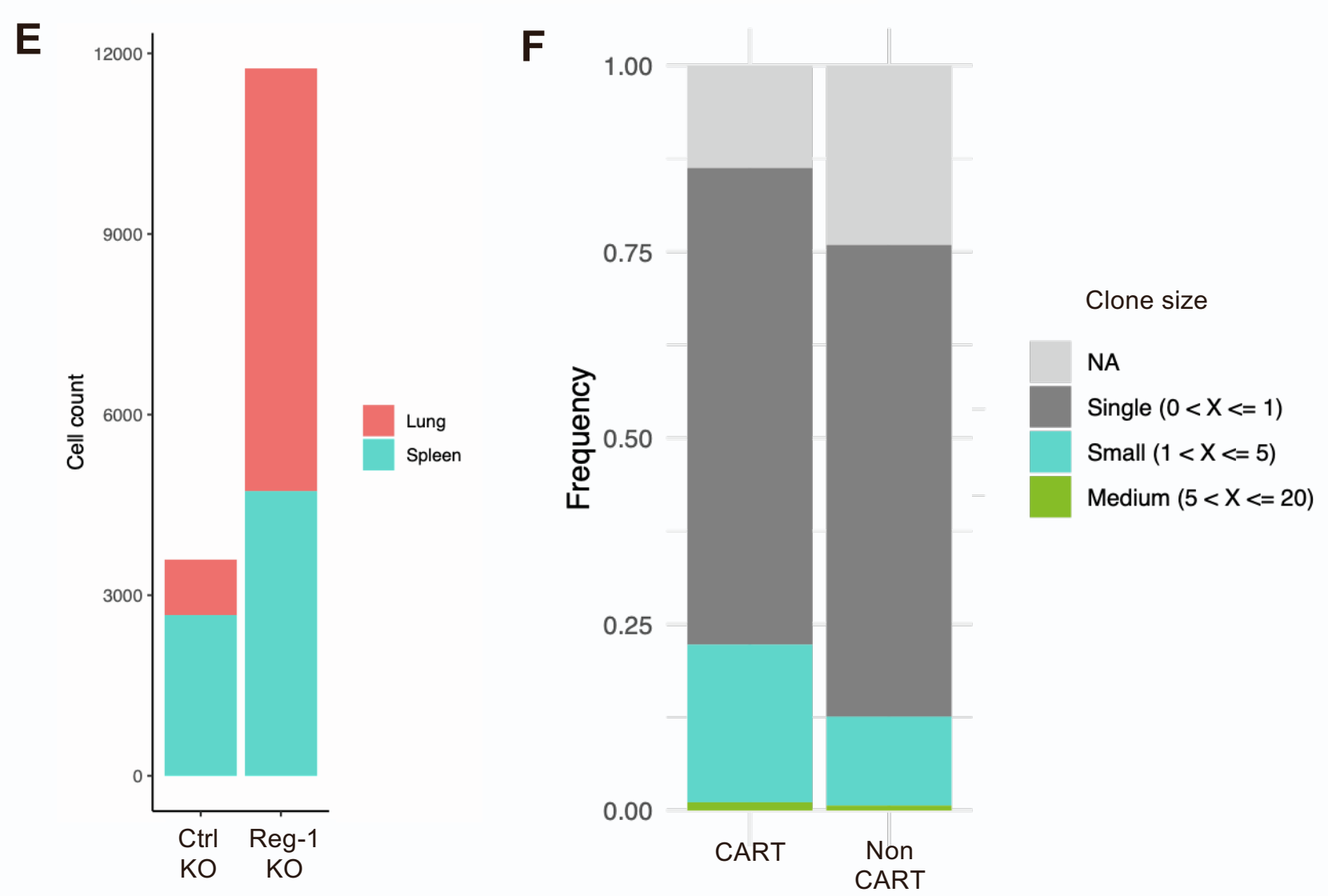

G

Clone Type	T-cell population	Cell number (n)
NA	Non-CART	723
NA	CART	1697
Single (0 < X ≤ 1)	Non-CART	1901
Single (0 < X ≤ 1)	CART	7900
Small (1 < X ≤ 5)	Non-CART	358
Small (1 < X ≤ 5)	CART	2610
Medium (5 < X ≤ 20)	Non-CART	19
Medium (5 < X ≤ 20)	CART	134

Chi-squared test, p-value < 2.2e-16

Figure S18: Reg-1 KO increased the frequency of clonally diverse CAR T cells in the lung, related to Figure 3. C57BL/6 mice (CD45.2$^+$) received F331 cells (i.v.) followed by a single dose of 5x10^6 Reg-1 KO or Ctrl KO B7-H3- CAR T cells (CD45.1$^+$) on day 7 post tumor cell injection. Lungs and spleens were isolated on day 5 post CAR T cell infusion, and cells were pooled from 3 mice for scRNA-seq profiling. (**A-D**) UMAP projection of (**A**) B7-H3-CAR T cells in the lungs and spleens, (**B**) Reg-1 KO and Ctrl KO B7-H3-CAR T cells within the lungs and spleens, (**C,D**) UMAP distrbution of B7-H3-CAR T and non-CAR T cells and (**D**) B7-H3-CAR expression in the lungs and spleens. (**E**) Cell number of Reg-1 KO compared to Ctrl KO B7-H3-CAR T cells in lungs and spleens. (**F**) scTCR-seq analysis showing T-cell clonal diversity in non-CAR and B7-H3-CAR T cells. (**G**) Analysis of data shown in panel F, including cell number per clone type. Chi-squared test comparing cell numbers of Non-CART and CART for each ‘clone type bucket’.

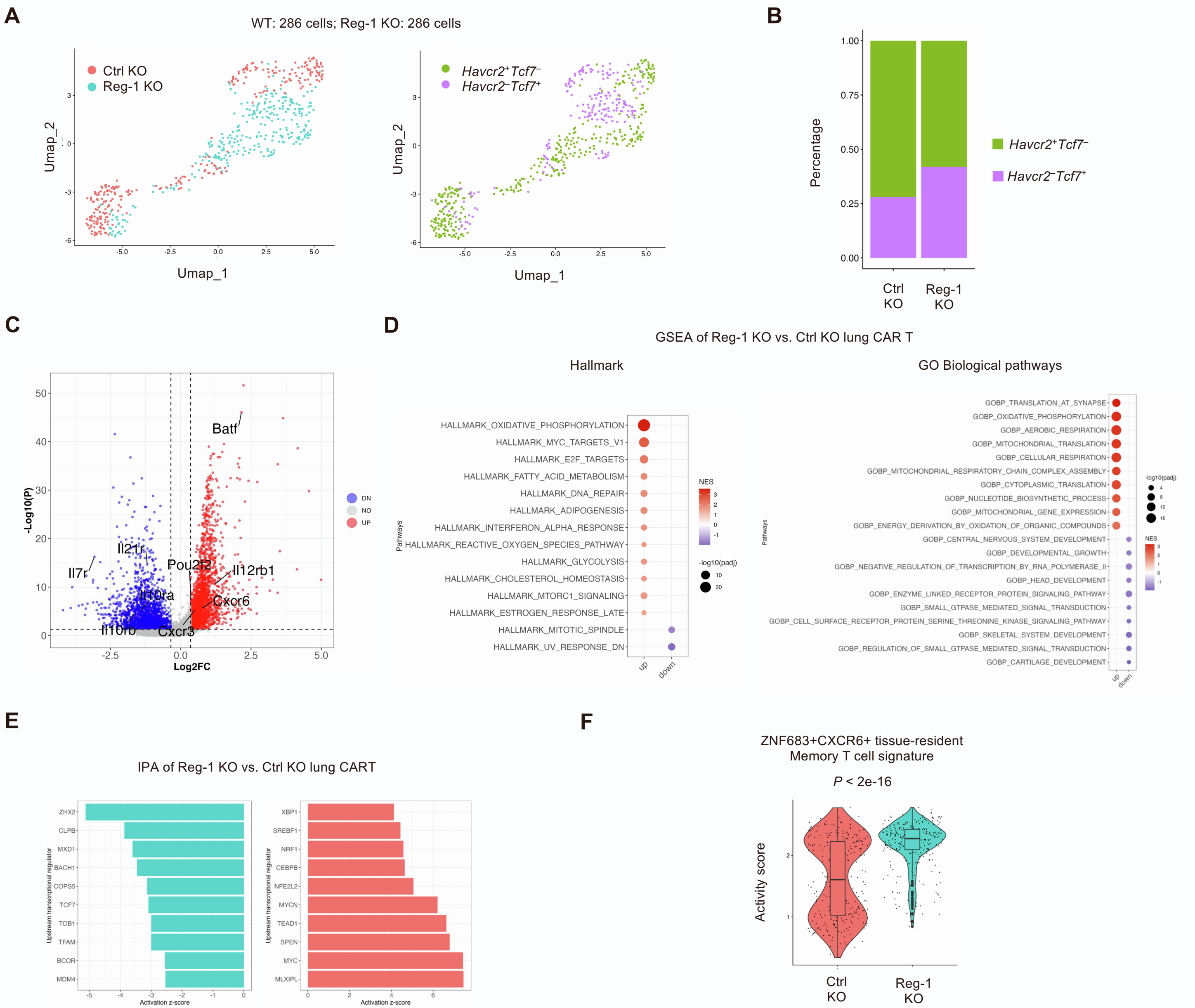

Figure S19: Repeat analysis using matched cell numbers for Ctrl and Reg-1 KO B7-H3-CAR T cells confirmed key findings shown in Figure 3D-I, related to Figure 3. Please see Figure 3 for details; 268 cells for each CAR T-cell populations were analyzed. (**A**) Uniform Manifold Approximation and Projection (UMAP) of Reg-1 KO or Ctrl KO B7-H3 CAR T cells (left) and *Hvacr2* (encoding TIM-3) or *Tcf7* (encoding TCF1) expression (right) in the lungs. (**B**) Proportion of *Havcr2* and *Tcf7* in the Reg-1 KO and Ctrl KO B7-H3-CAR T cells in the lungs. (**C**) Upregulated (red) or downregulated (blue) genes ($log_2FC > 0.5$ and adjusted $P < 0.05$) in Reg-1 KO B7-H3-CAR T cells compared to Ctrl KO B7-H3-CAR T cells. (**D**) Top 12 significantly (FDR < 0.05) upregulated and top 2 downregulated metabolism-related pathways (from Hallmark gene sets; left) or top 10 upregulated and downregulated GO biological pathways (right) in Reg-1 KO B7-H3-CAR T cells compared to Ctrl KO B7-H3-CAR T cells, as revealed by GSEA. (**E**) Ingenuity pathway analysis of transcriptional regulators of Reg-1 KO B7-H3-CAR T cells compared to Ctrl KO B7-H3-CAR T cells in the lungs. (**F**) Activity score of a CD8⁺ ZNF683⁺CXCR6⁺ tissue-resident memory T cell signature in Ctrl KO and Reg-1 KO B7-H3-CAR T cells in the lungs.

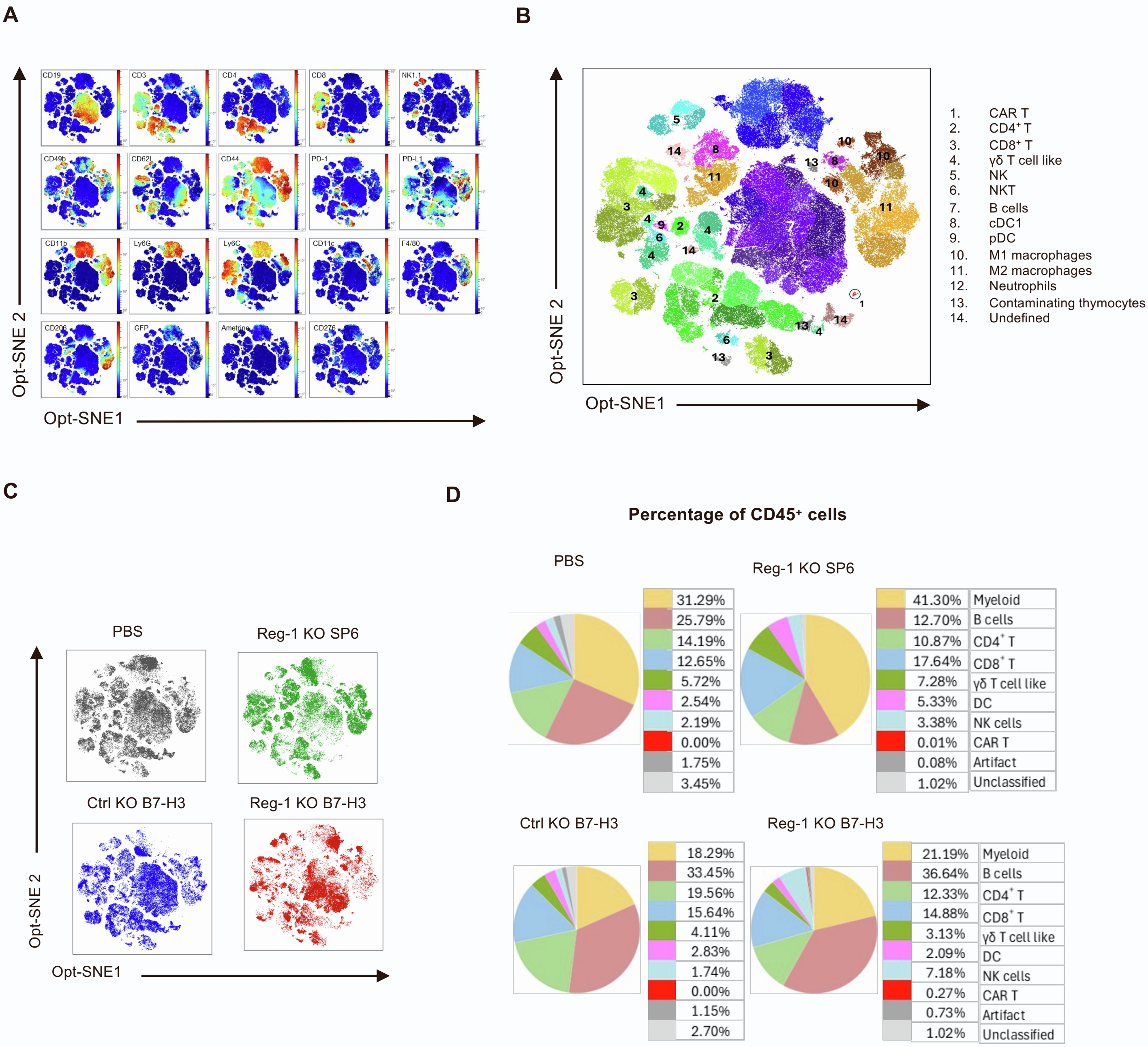

Figure S20: Global immune landscape changes post B7-H3-CAR T cell infusion, related to Figure 4. C57BL/6 mice ($CD45.2^+$) were injected with $1x10^6$ F331 cells (i.v.) and on day 7 received a single i.v. dose of edited CAR T cells ($Cas9^+CD45.1^+Ametrine^+$) population or PBS. On day 21 post CAR T cell infusion, mice were euthanized and lungs isolated for high dimensional flow cytometry analysis using a panel including 19 unique immune cell markers. An unbiased algorithm based opt-SNE analysis was performed on 26,000 viable $CD45^+$ lung cell isolates per sample, followed by an unguided clustering in Flow-SOM utilizing elbow metaclustering. (**A**,**B**) Concatenated data from the lungs of 12 mice. (**A**) Heatmaps presenting the relative mean fluorescence intensity of the immune markers assayed, localized across the t-SNE clusters. (**B**) t-SNE cluster grouping according to immune subtypes based on root phenotypic marker expression detailed in Table S2. (**C**) t-SNE plots for indicated treatment conditions (n=3 mice per condition). (**D**) Fraction of $CD45^+$ cells assigned to each immune cell subset, given as the mean from the 3 individuals in the indicated treatment group. t-SNE, t-distributed stochastic neighbor.

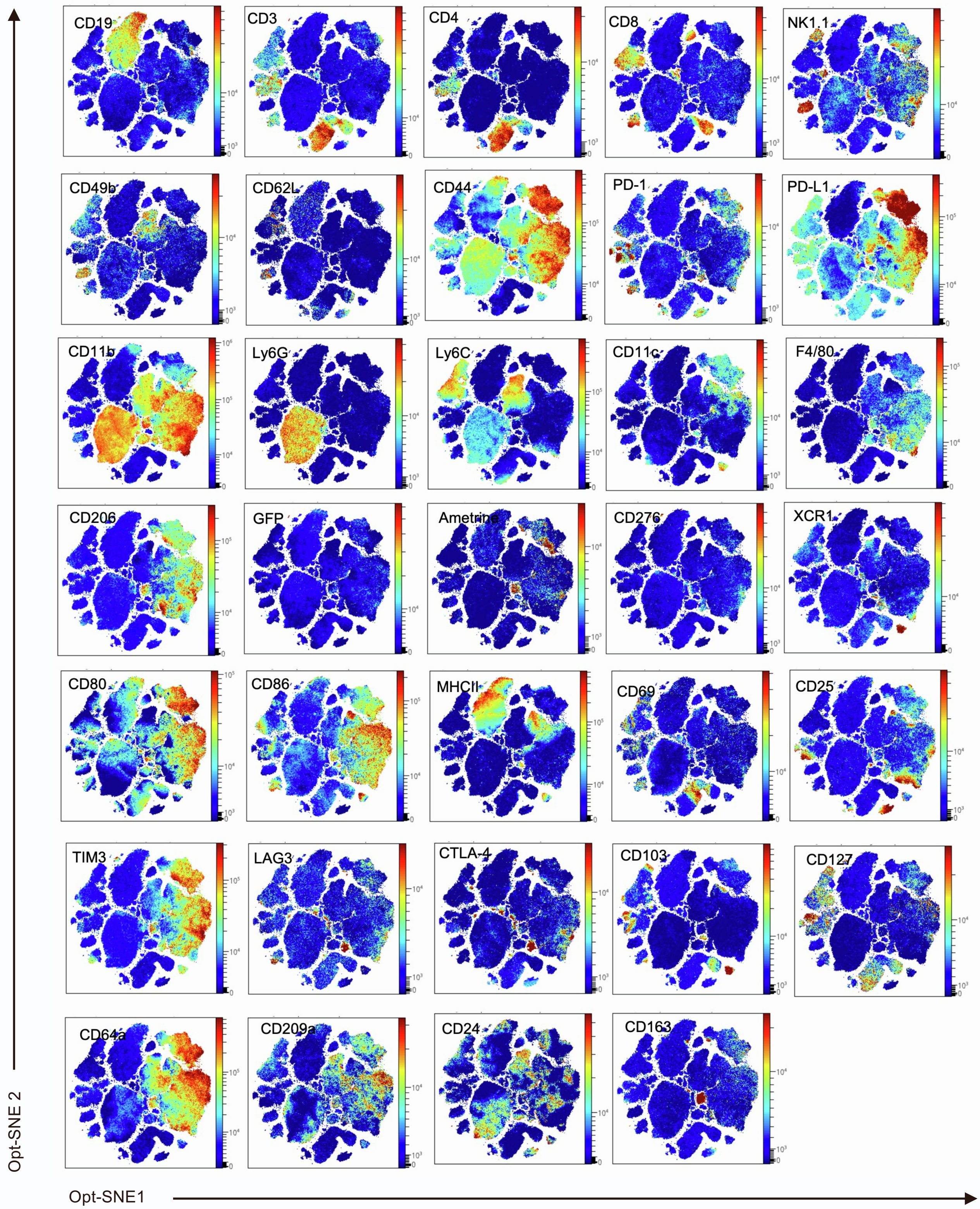

Figure S21: Marker expression across t-SNE associations in high-dimensional phenotyping analysis for 38-marker panel, related to Figure 4. Heatmaps present the relative mean fluorescence intensity of the immune markers assayed, localized across the several t-SNE clusters. t-SNE, t-distributed stochastic neighbor embedding.

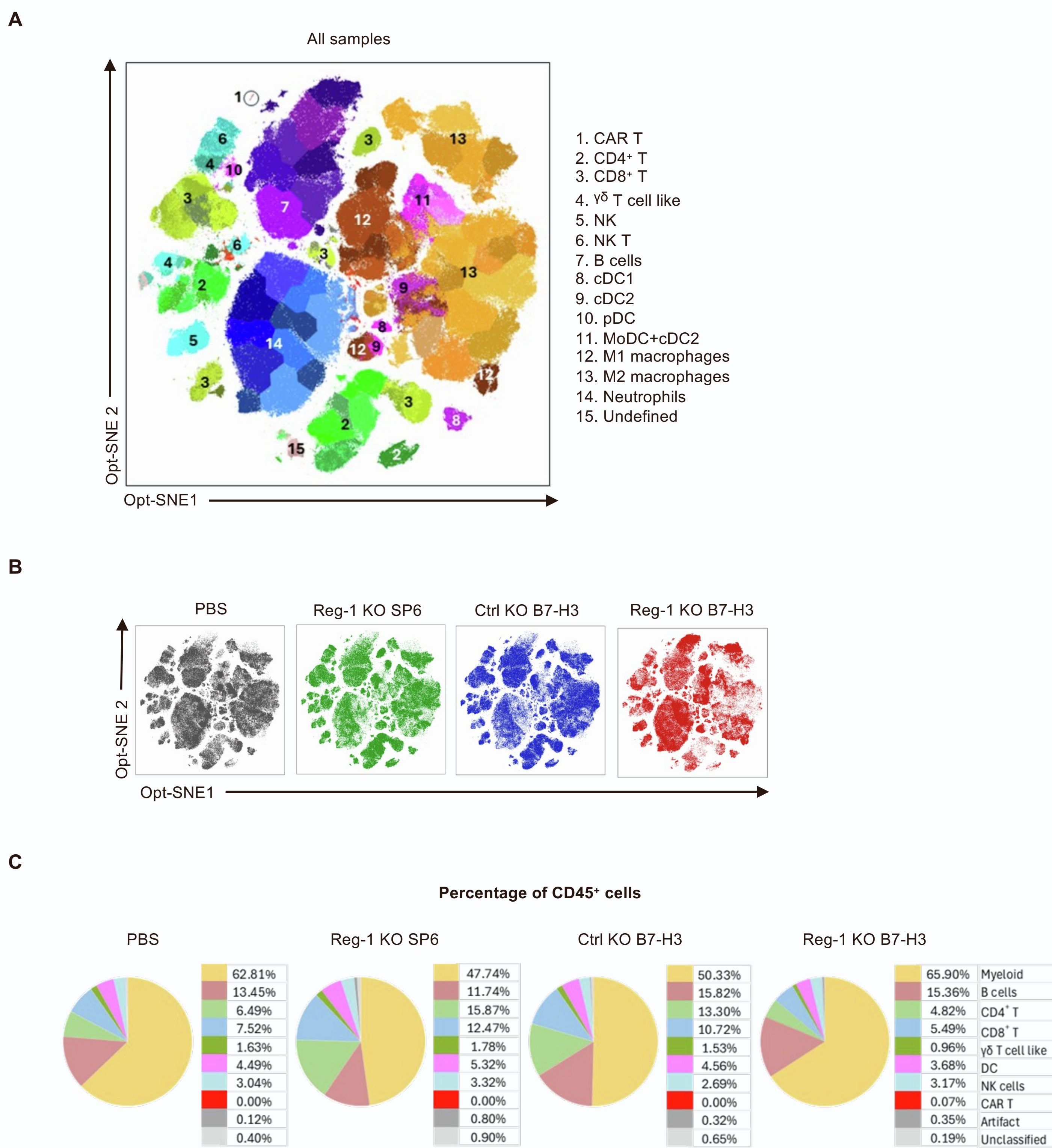

Figure S22: Frequency of distinct immune cells subsets among CD45⁺ cells, related to Figure 4. (**A**) t-SNE analysis of concatenated data from all 16 mice analyzed with the 38-marker panel described for Fig. S21 with phenotyping conducted as described in Table S4. (**B**) t-SNE plots for individual treatment conditions (n=4 mice per group). (**C**) Fraction of CD45⁺ cells assigned to each immune cell subset, given as the mean from the 4 individuals in the indicated treatment group. t-SNE, t-distributed stochastic neighbor embedding.

A

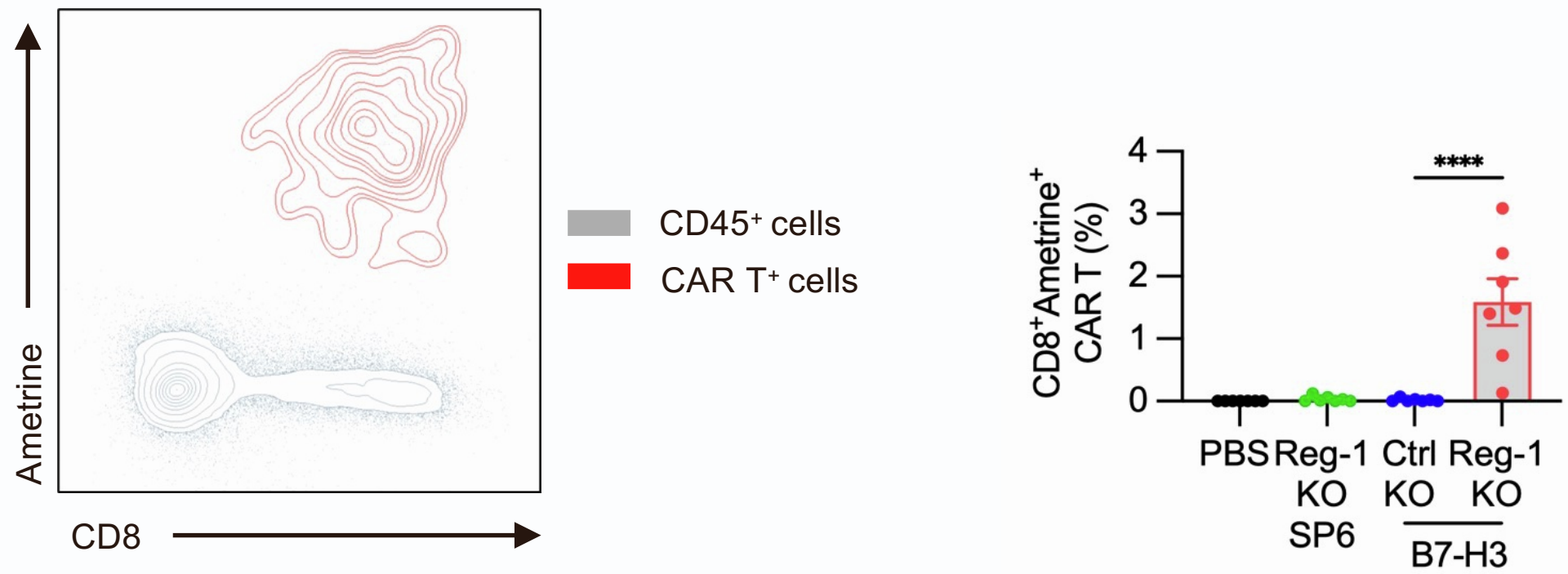

B

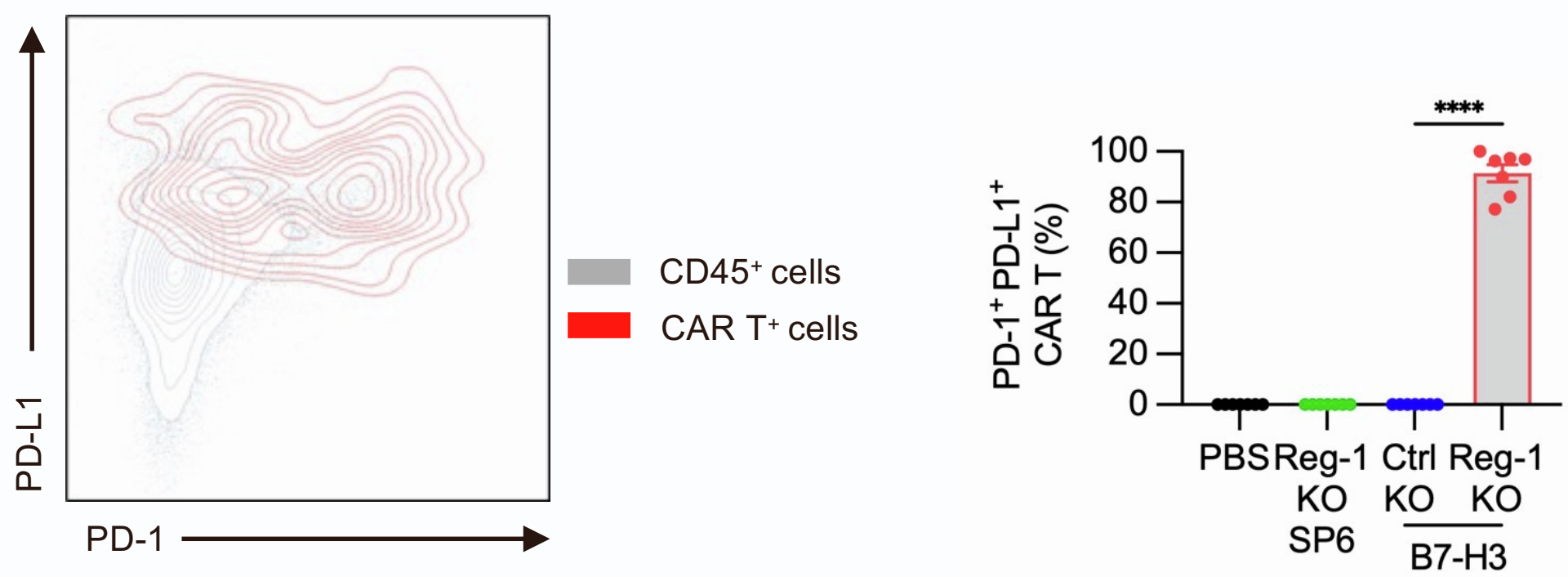

Figure S23: Reg-1 KO enhances B7-H3-CAR T-cell persistence on day 21 post infusion, related to Figure 4. Data was pooled from all 7 mice in each condition between both immunophenotyping experiments described for Fig. S20 and Fig. S22. (**A**) Bar graphs gives the fraction of CAR T cells observed under each treatment condition from total $CD45^+$ cells recovered, showing a unique persistence for Reg-1 KO B7-H3-CAR T cells. (**B**) Bar graphs gives the fraction of $PD\text{-}1^+PD\text{-}L1^+$ CAR T cells, indicating that nearly all CAR T cells persisting at this time point expressed these markers. Contour plots in (**A**, **B**) show co-expression (indicated in red) of Ametrine and CD8 (**A**) or PD-L1 and PD-1 (**B**) populations relative to concatenated data from all $CD45^+$ cells (grey); Data from two independent experiments represented as mean ± SEM. One-way ANOVA; ****:$p<0.0001$;

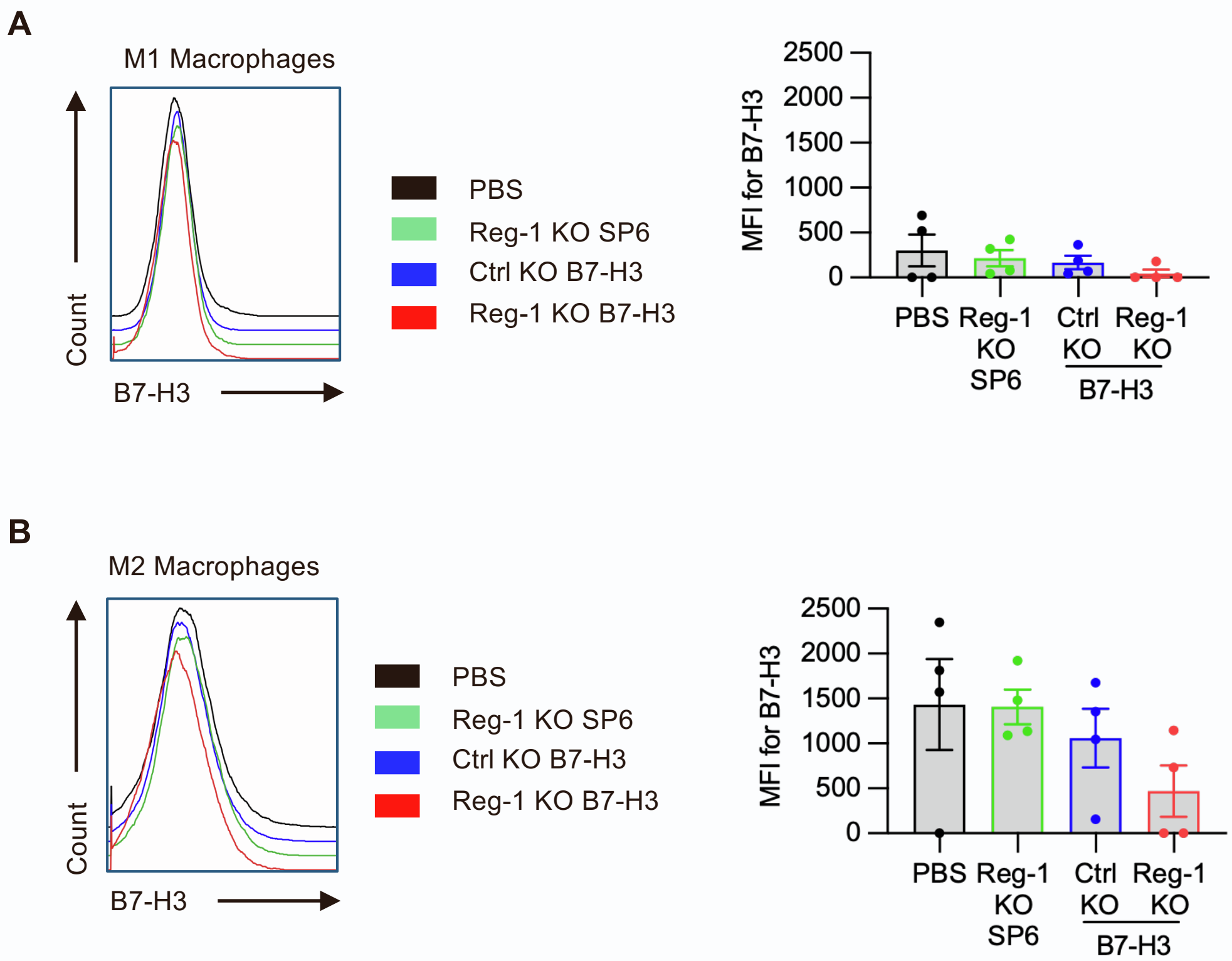

Figure S24: B7-H3 expression on lung-infiltrating macrophage subsets from F331 mice post-CAR T cell treatment, related to Figure 4. For experimental details, see Fig. S21-22 (**A**) Representative histogram showing B7-H3 expression (left) and MFI for B7-H3 (right) on M1 (A) and M2 (B) macrophages from F331 mice (n = 4). evaluated by flow cytometry on day 21 post CAR T cell infusion. Data are represented as mean ± SEM.

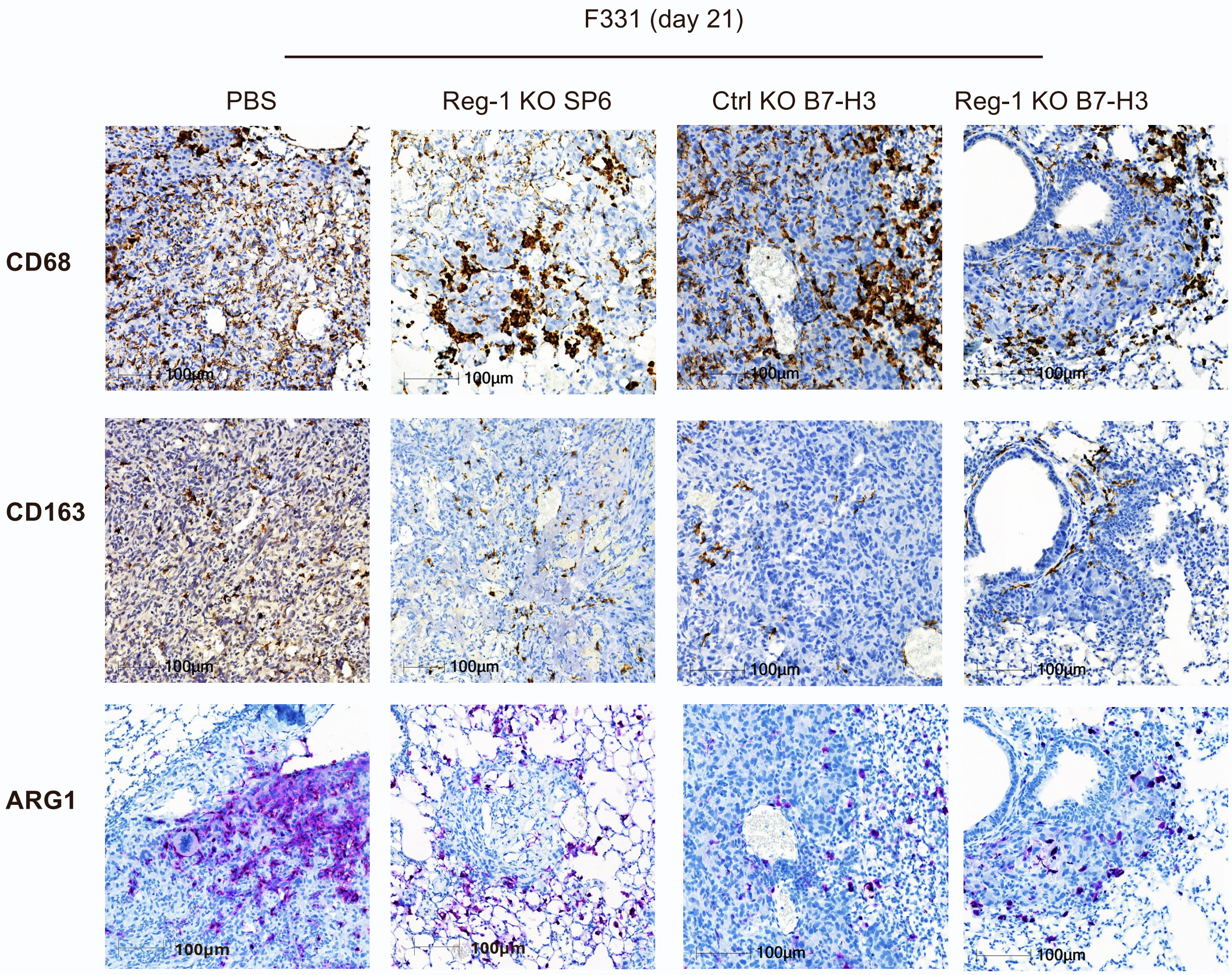

Figure S25: Reg-1 KO B7-H3-CAR T cells decrease macrophage signatures in osteosarcoma, related to Figure 4. IHC for a universal-macrophage marker (CD68 staining) or specific markers for M2 macrophages (CD163 and ARG1) in lung tumors from F331 tumor-bearing mice on day 21 after infusion with Reg-1 KO SP6, Ctrl KO or Reg-1 KO B7-H3-CAR T cells. 3,3'-Diaminobenzidine (DAB) chromogen with hematoxylin counterstain. Representative images. 20x magnification, 100 µm scale.

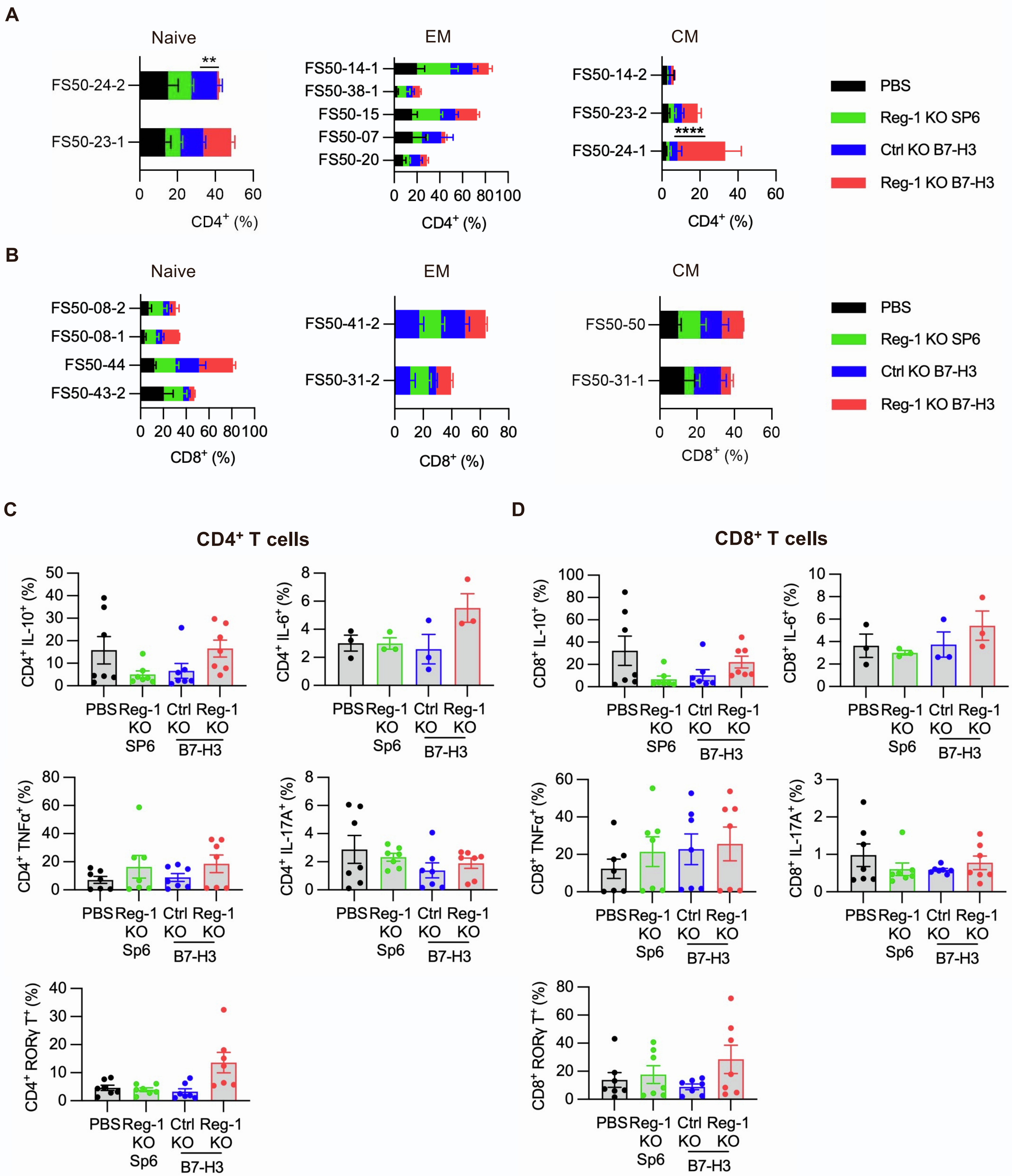

Figure S26: Reg-1 KO B7-H3-CAR T cells alter endogenous T-cell subsets and promote cytokine production of T cells, related to Figure 4. (**A, B**) The naive, EM and CM subsets of (**A**) CD4+ and (**B**) CD8+ T cell clusters (n=3 mice per group) related to Fig. 4G,H. Comparison was performed for the Reg-1 KO B7-H3-CAR T treatment condition relative to the Ctrl KO B7-H3-CAR T cell recipients. (**C,D**) Intracellular flow cytometry panel detailed in Table S6 and outlined in method section was conducted on an aliquot of single-cell suspensions from lungs of F331 tumor-bearing mice that received infusion of the indicated CAR T cells. Percentages of IL-10, IL-6, TNFα, IL-17A, and RORγ-expressing cells among (**C**) CD4+ and (**D**) CD8+ T cells. Data are represented as mean ± SEM. One-way and Two-way ANOVA, Tukey's multiple comparisons test; **:p<0.01; ****:p<0.0001.

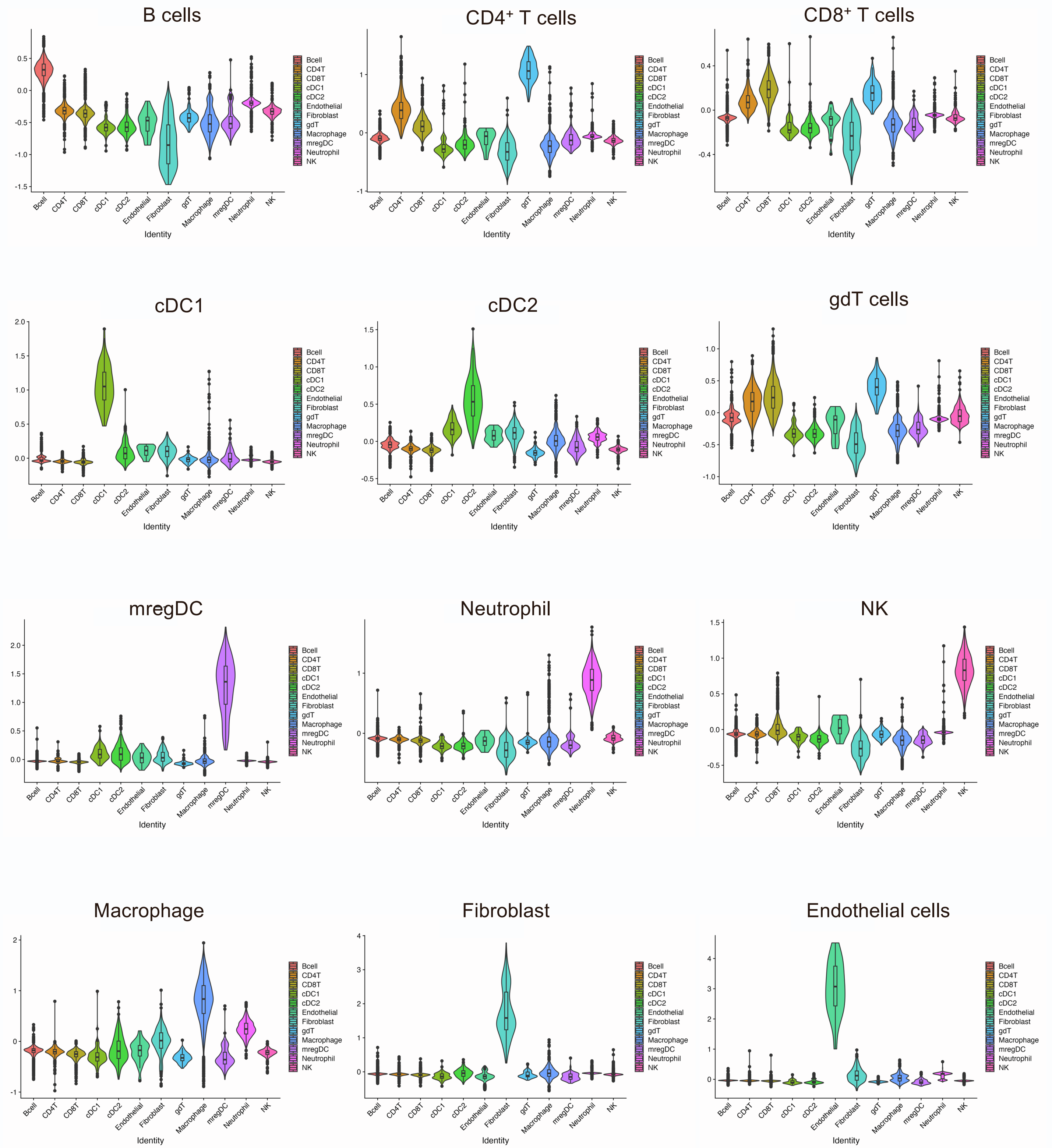

Figure 27: scRNA-seq annotation of lung-infiltrating immune cell subsets on day 21, related to Figure 5. Violin plots depicting cell type annotation of the immune cell subsets shown in Fig. 5B**.** For experimental details, see Fig. 5A.

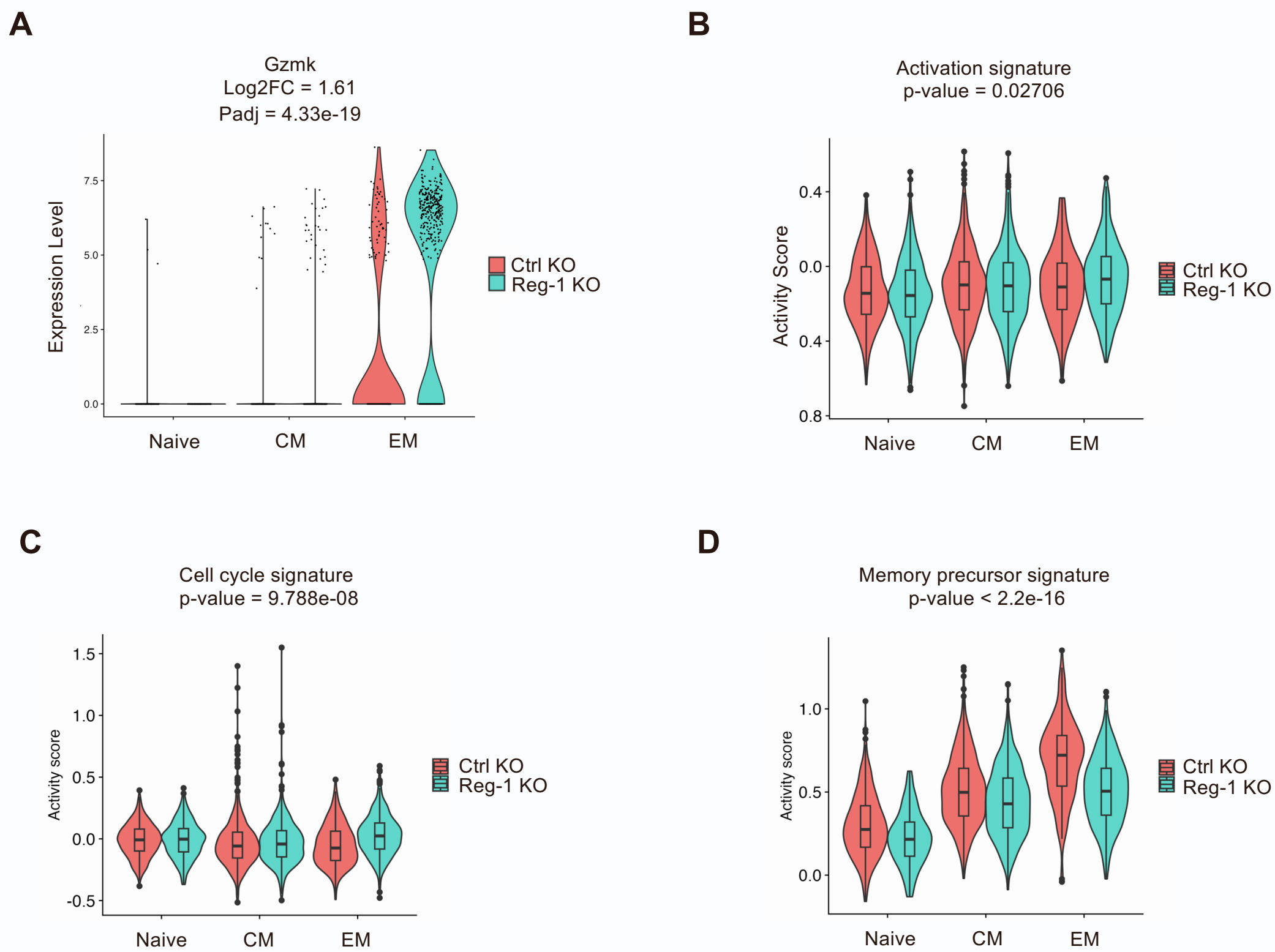

Figure 28: Transcriptional profiling reveals enhanced activation and effector differentiation of endogenous *Cd8⁺* T cells induced by Reg-1 KO B7-H3 CAR T cells, related to Figure 5. (A-D) Expanded analysis of Cd8+ T cells subsets perturbation related to **Fig. 5D** showing Gzmk expression (A), activation signature (B), cell cycle signature (C) memory precursor signature (D) among naïve, central memory (CM) and effector memory (EM) populations.

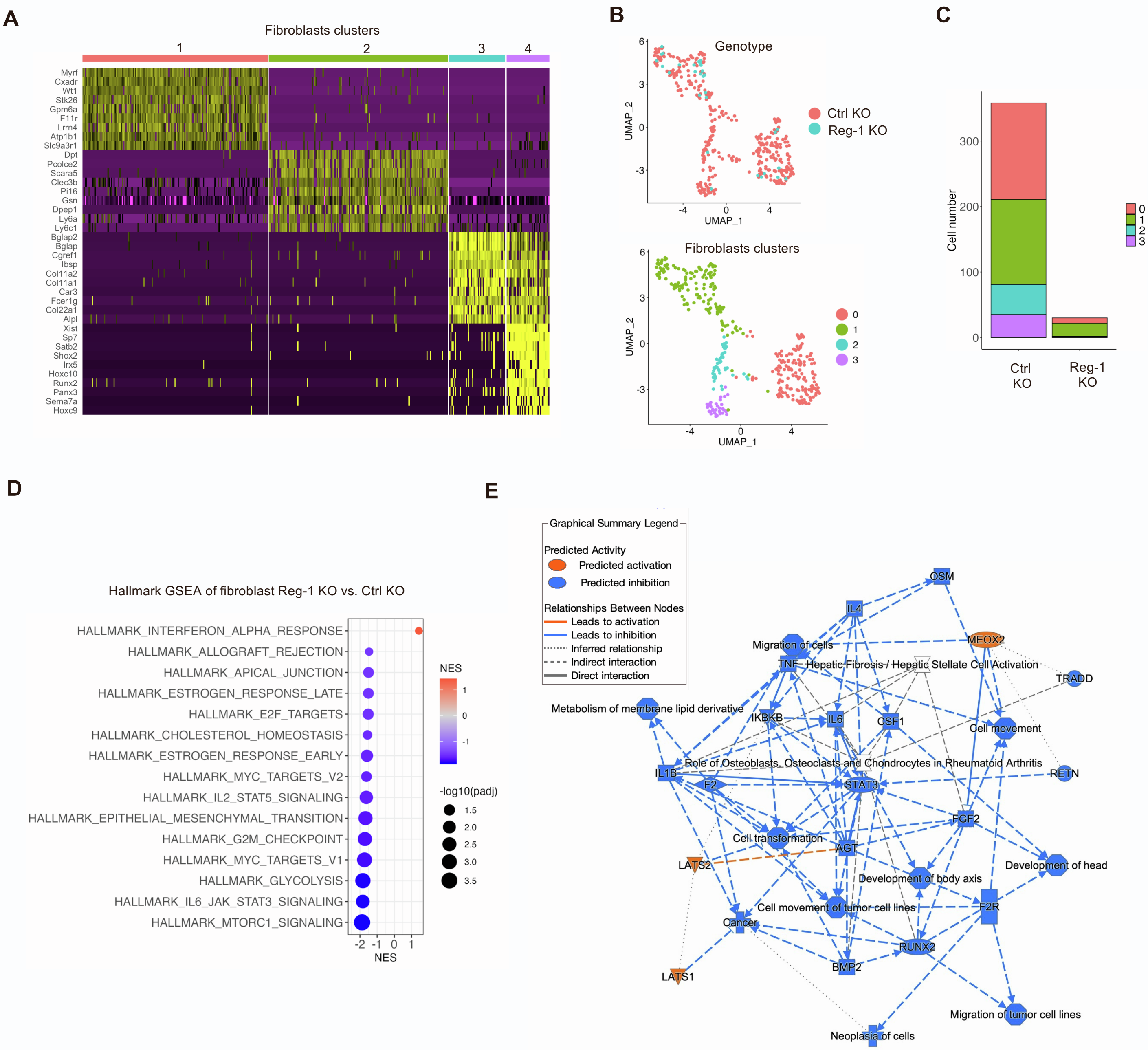

Figure S29: Reg-1 KO B7-H3-CAR T cells induced changes in lungs infiltrating fibroblasts, related to Figure 5. (**A**) Heatmap gene expression visualization of fibroblasts reveals four distinct fibroblast clusters. (**B**) UMAP projection of fibroblasts in the lungs after infusion with Reg-1 KO or Ctrl KO B7-H3-CAR T cells (n=2); genotype (top) or clusters (botton) are illustrated. (**C**) Absolute cell numbers of fibroblast clusters. (**D**) Significantly (FDR < 0.05) upregulated and top 14 downregulated pathways in fibroblasts, based on GSEA with Hallmark gene sets. (**E**) Ingenuity pathway analysis of fibroblasts in the lungs of mice that received Reg-1 KO compared to Ctrl KO B7-H3-CAR T cells.

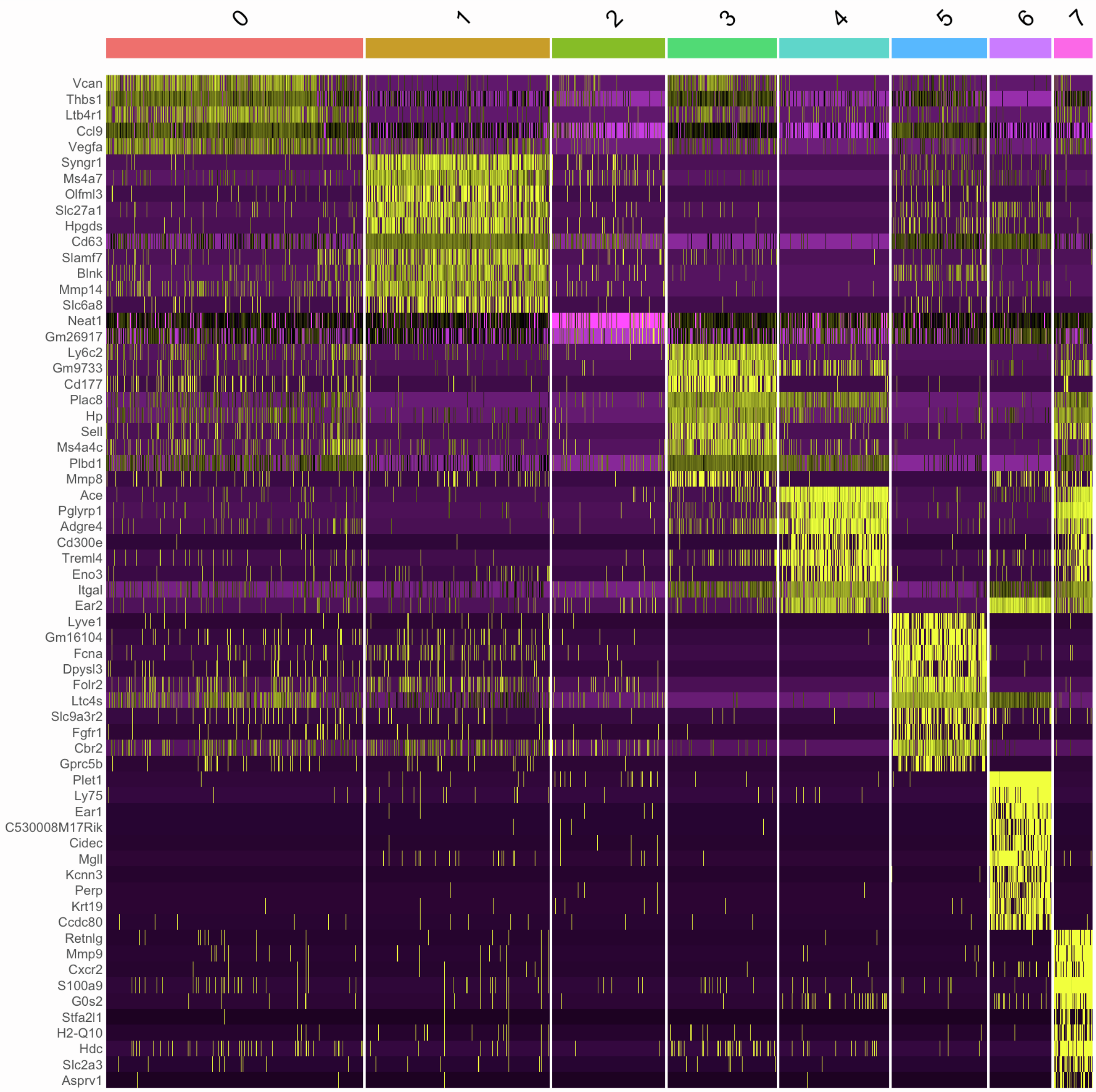

Figure S30: Heatmap gene expression visualization of macrophages reveals eight distinct macrophage clusters, related to Figure 6. For experimental details, see Fig. 6.

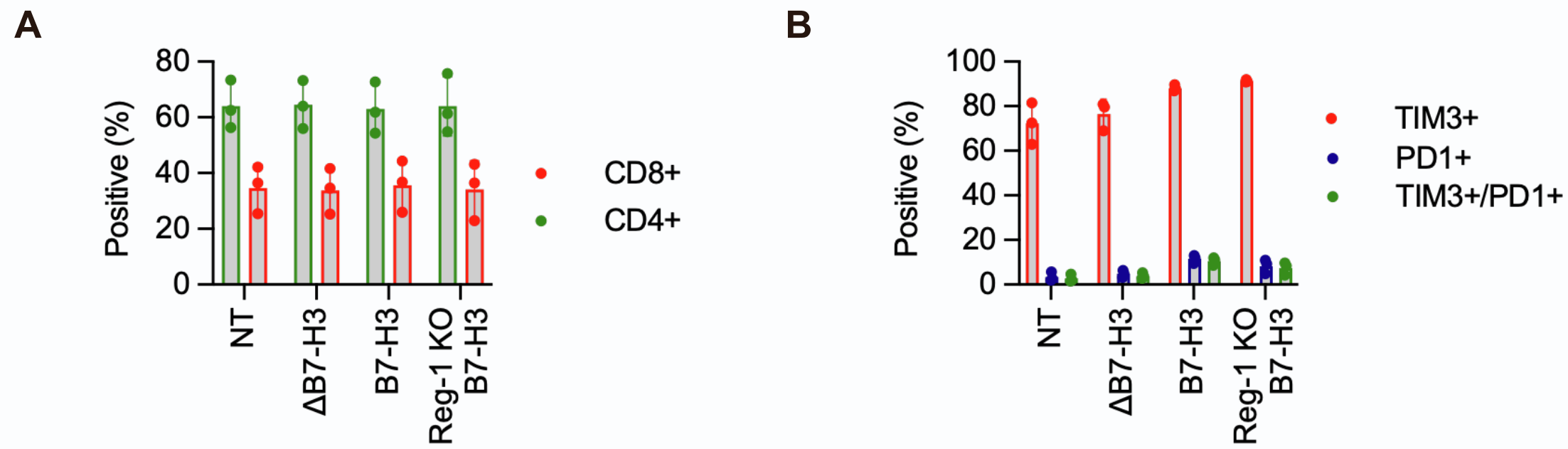

Figure S31: Immunophenotyping of human CAR T cells, related to Figure 7. (**A, B**) Immunophenotyping of human CAR T cells generated for the experiment described in Fig. 7. (**A**) percentages of $CD4^+$ and $CD8^+$ T cells; (B) percentages of $TIM3^+$, $PD1^+$ and $TIM3/PD1^+$ cells; n= 3 healthy donors. Data are represented as mean ± SEM.

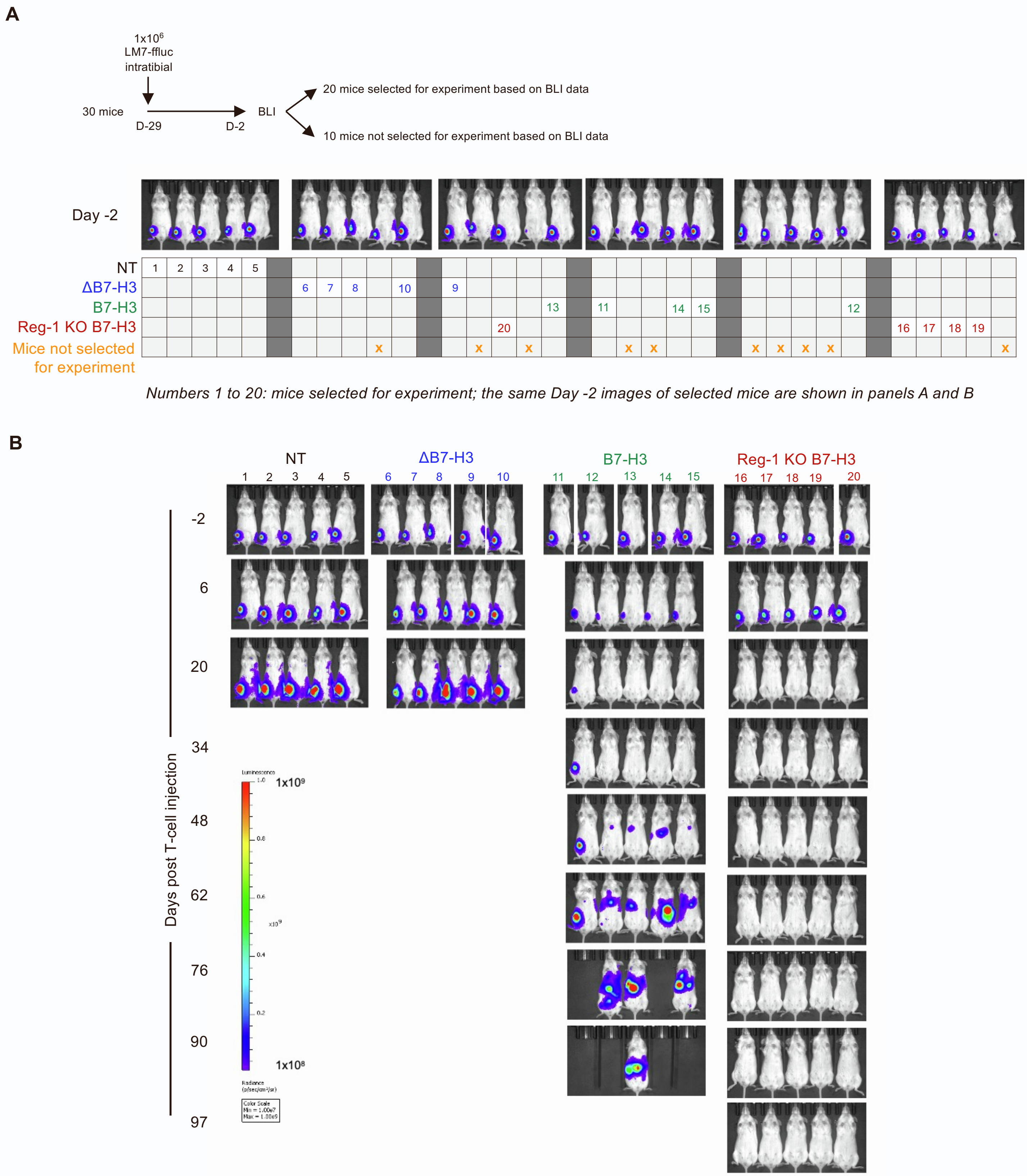

Figure S32: Reg-1 KO B7-H3-CAR T cells have improved antitumor activity *in vivo* and prevent development of pulmonary metastases in orthotopic OS model, related to Figure 7. (A) Scheme to select mice for experiment. 30 mice were implanted and on day -2 groups were assigned based on quantitative bioluminescence data as indicated below each image. (**B**) Representative images for one donor for the quantitative bioluminescence image data shown in Fig. 7G. Day -2 images are cropped due to group assignment and are the same images as shown in panels A and B of the selected mice.

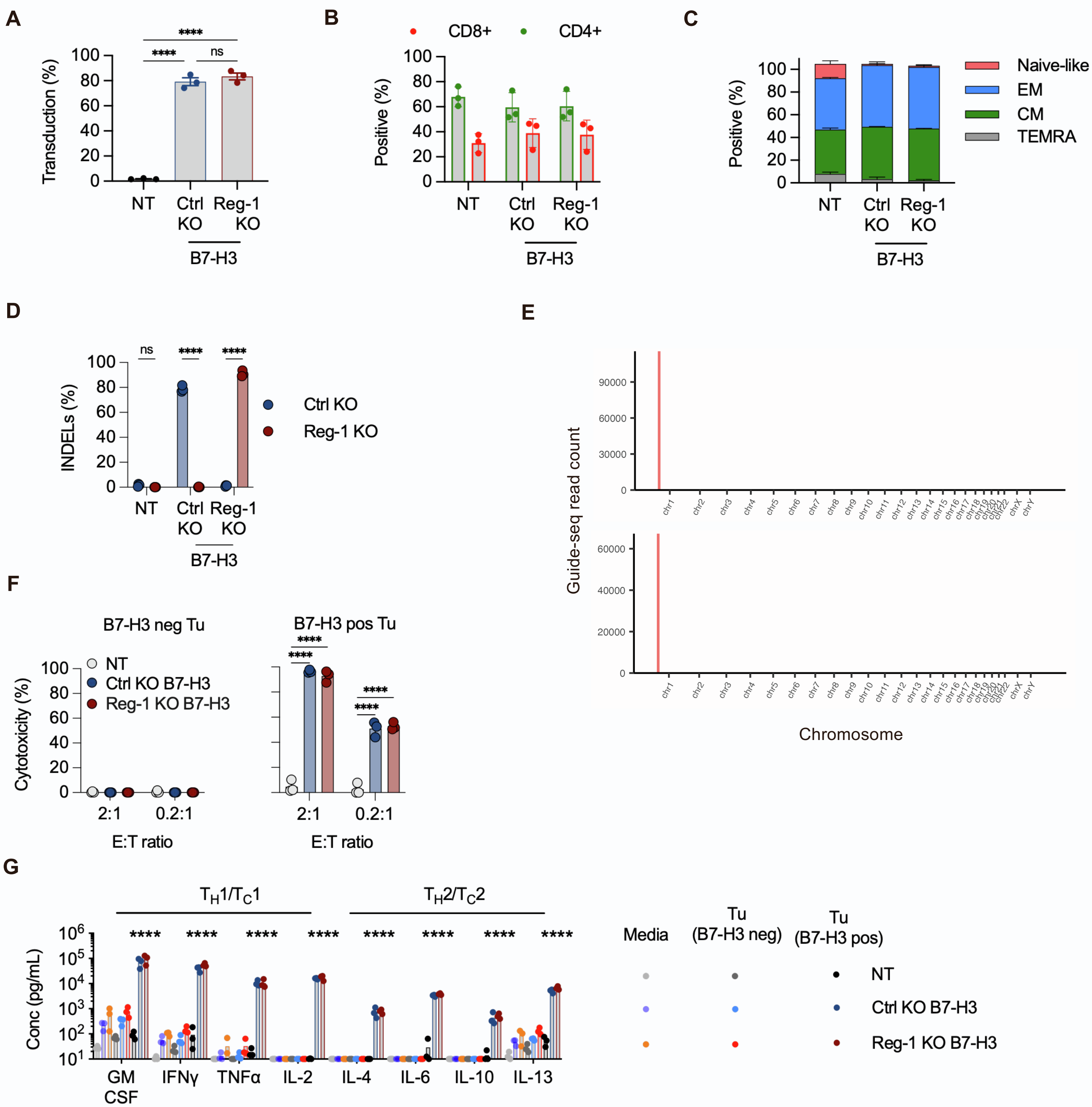

Figure S33: Characterization of human Reg-1 KO B7-H3-CAR T cells, related to Figure 7. Representative data from 3 donors except for Guide-seq-2 analysis (n=2). (**A**) Transduction efficiency of Ctrl KO and Reg-1 KO B7-H3-CAR T cells and non-transduced (NT) T cells, evaluated by flow cytometry, n=3. (**B, C**) Immunophenotyping for (**B**) CD4 and CD8, and (**C**) T cell subsets; naive-like ($CCR7^+CD45RO^-$), CM ($CCR7^+CD45RO^+$), EM ($CCR7^-CD45RO^+$) and TEMRA ($CCR7^-CD45RO^-$), Mean ± SEM. (**D**) Insertion and deletion (Indel) analysis. (**E**) Manhattan plots showing the genome-wide distribution of GUIDE-seq-2 detected editing sites with chromosomal position (x-axis) and read counts (y-axis). (**F**) ffluc-based cytotoxicity with B7-H3 positive (THP1) and B7-H3 negative (KG1a) target cells. (**G**) Coculture assay with THP and KG1a tumor cells. Media was collected and cytokine production was evaluated by ELLA. Data are represented as mean ± SEM. Statistical analysis for panels A, D, F, and G: two-way ANOVA with Tukey's test for multiple comparisons; ****p<0.001; for panel G, p values are shown for media vs B7-H3 target comparison for Ctrl KO and Reg-1 KO B7-H3-CAR T cells.

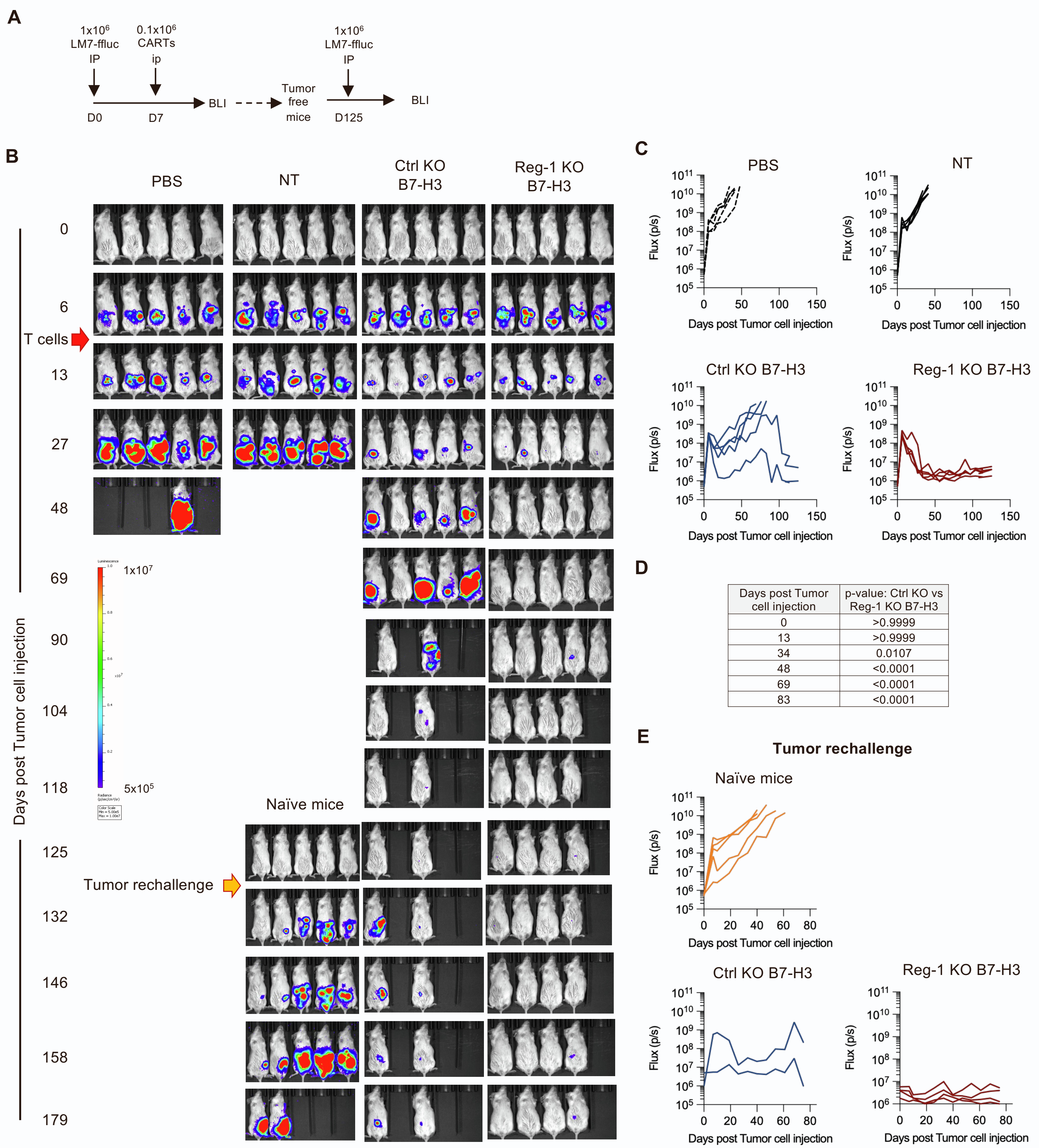

Days post Tumor cell injection	p-value: Ctrl KO vs Reg-1 KO B7-H3
0	>0.9999
13	>0.9999
34	0.0107
48	<0.0001
69	<0.0001
83	<0.0001

Figure S34: Reg-1 KO improves antitumor activity of B7-H3-CAR T cells and their functional persistence, related to Figure 7. (**A**) Experimental scheme. NSG mice received 1x10^6 LM7-ffluc cells intraperitoneally (i.p.) and on day 7 non transduced (NT), Ctrl KO B7-H3-CAR, or Reg-1 KO B7-H3-CAR T cells (1x10^5), or PBS i.p. (n=5 mice per group). On day 125 post tumor cell injection, long-term survivors were rechallenged with a second injection of 1x10^6 LM7-ffluc cells. (**B**) Representative bioluminescence images. One mouse in the Reg-1 KO B7-H3-CAR T cell group was euthanized due to signs of distress (hunched back, diarrhea) on day +90. (**C**) Quantified bioluminescence data until day +118 post 1st tumor cell injection. (**D**) Comparison between Ctrl KO B7-H3-CAR and Reg-1 KO B7-H3-CAR T cells and indicated time points, two-way ANOVA with Tukey's test for multiple comparisons. (**E**) Quantified bioluminescence data starting day +125 post 1st tumor cell injection.

Table S1: Statistical analysis of F331 co-culture experiments, related to Figure 1

TH1 Cytokine	NT vs Reg-1 KO SP6	NT vs Ctrl KO B7-H3	NT vs Reg-1 KO B7-H3	Reg-1 KO SP6 vs Ctrl KO B7-H3	Reg-1 KO SP6 vs Reg-1 KO B7-H3	Ctrl KO B7-H3 vs Reg-1 KO B7-H3
**G-CSF**	ns	ns	ns	ns	ns	ns
**M-CSF**	ns	*	*	ns	ns	ns
**GM-CSF**	ns	****	****	****	****	ns
**TNFα**	ns	****	****	****	****	ns
**IL-1α**	ns	****	****	****	****	ns
**IL-1β**	ns	ns	ns	ns	ns	ns
**IL-2**	*	****	****	****	****	ns
**IL-3**	ns	****	****	****	****	ns
**IL-7**	ns	ns	***	ns	***	ns
**IL-12p40**	ns	ns	ns	ns	ns	ns
**IL-12p70**	ns	*	ns	ns	ns	ns
**IL-15**	ns	****	****	****	****	ns
**IFNγ**	ns	****	****	****	****	ns
**TH2/17 Cytokines**						
**IL-4**	ns	ns	ns	ns	ns	ns
**IL-6**	ns	**	****	*	***	ns
**IL-9**	ns	ns	ns	ns	ns	ns
**IL-10**	ns	***	****	**	****	ns
**IL-13**	ns	***	****	***	***	ns
**IL-17**	ns	ns	*	ns	ns	ns
**LIF**	ns	****	****	****	****	ns
**Chemokines**						
**IP-10**	ns	****	****	**	**	ns
**CXCL1**	ns	*	**	*	*	ns
**LIX**	ns	*	**	*	**	ns
**MCP-1**	ns	**	***	**	**	ns
**MIP-1a**	**	****	****	****	****	ns
**MIP-1B**	***	****	****	****	****	ns
**MIP-2**	ns	**	**	ns	ns	ns
**MIG**	**	****	****	****	****	ns
**RANTES**	**	****	****	****	****	ns
**VEGF**	ns	****	****	***	***	ns

Two-way ANOVA with Tukey's test for multiple comparisons. *:$p<0.05$, **:$p<0.01$, ***:$p<0.001$, ****:$p<0.0001$.

Table S2: List of Extracellular flow cytometry Panel with 23 fluorophores, related to Figure 4, Figure S11, Figure S14 - S16 and Figure S23

Antibody	Conjugate	Clone	Source	Identifier
CD11b	BUV395	M1/70	BD	563553
CD11c	BUV615	HL3	BD	751265
CD19	BUV661	1D3	BD	612971
F4/80	BUV737	T45-2342	BD	749283
CD4	BUV805	GK1.5	BD	564922
CD62L	eFluor 450	MEL-14	Thermo	48-0621-82
CD3	BV480	500A2	BD	746776
Ly6C	BV570	HK1.4	BioLegend	128030
CD44	BV605	ID7	BD	563058
CD8a	BV711	53-6.7	BD	563046
CD45.1	BV786	A20	BD	740889
Ly6G	PerCP	1A8	BioLegend	127654
CD279 (PD-1)	BB700	J43	BD	566514
CD274 (PD-L1)	PE	10F.9G2	BioLegend	124308
CD49b	PE-Cy5.5	DX5	Thermo	35-5971-82
CD206	PE-Cy7	C068C2	BioLegend	141720
CD276 (B7-H3)	APC	MIH35	BioLegend	135607
CD45.2	Alexa Fluor 700	104	Thermo	56-0454-82
NK1.1	APC-Cy7	PK136	BD	560618
CD45	APC-Fire 810	30-F11	BioLegend	103174
**Other Fluorophores Present:**				
Live/Dead Fixable Blue	N/A	N/A	Thermo	L23105
GFP				
Ametrine				
		23 Total Chanels		

Table S3: List of FlowSOM clusters from 23-fluorophore flow cytometry panel, related to Figure 4, Figure S20, S23

Cluster ID:		Cluster Phenotype
**FS50-04**	CAR-T	CD8(+++) CD44(++) CD62dL(+++) PDL1(+++) PD1(++) CD49b(++) Ly6C(++)
CAR-T Root definition:		CD3(+) CD11b(-) CD19(-) Ametrine(+)
**FS50-24-1**	CD4-T, CM	PDL1(++) PD1(-) Ly6C(+)
**FS50-23-2**	CD4-T, CM	PDL1(++) PD1(-/Lo)
**FS50-14-2**	CD4-T, CM	PDL1(+++) PD1(-) CD49b(-) Ly6c(-)
**FS50-20**	CD4-T, EM	PDL1(+) Ly6C(+)
**FS50-07**	CD4-T, EM	PDL1(++)
**FS50-15**	CD4-T, EM	PDL1(++) PD1(Lo/-) CD49b(++) Ly6C(Lo/-)
**FS50-38-1**	CD4-T, EM	PDL1(++) PD1(Lo/-) CD49b(++) NK1.1(-) Ly6C(+++) CD11c(-)
**FS50-14-1**	CD4-T, EM	PDL1(+++) PD1(+++) CD49b(+) Ly6c(Lo/-)
**FS50-23-1**	CD4-T, Naïve	PDL1(Lo/-) PD1(-)
**FS50-24-2**	CD4-T, Naïve	PDL1(Lo/-) PD1(-) Ly6C(+)
CD4-T Root:		CD3(+) CD11b(-) CD19(-) CD4(+) CD8(-)
Naïve:		CD62L(+) CD44(-)
CM (Central memory):		CD62L(+) CD44(+)
EM (Effector memory):		CD62L(-) CD44(+)
**FS50-31-1**	CD8-T, CM	PDL1(+) PD1(-)
**FS50-43-2**	CD8-T, CM	PDL1(+) PD1(-) Ly6C(+++)
**FS50-50**	CD8-T, CM	PDL1(+) PD1(-) Ly6C(+++)
**FS50-44**	CD8-T, EM	PDL1(++) PD1(-/Lo) CD49b(++) Ly6C(++)
**FS50-08-1**	CD8-T, EM	PDL1(++) PD1(-/Lo) CD49b(++) Ly6C(Lo/-)
**FS50-08-2**	CD8-T, EM	PDL1(++) PD1(+++) CD49b(++) Ly6C(Lo/-)
**FS50-31-2**	CD8-T, Naïve	PDL1(-) PD1(-)
**FS50-41-2**	CD8-T, Naïve	PDL1(-) PD1(-) Ly6C(++)
**FS50-42**	CD8-T	CD44(-) CD62L(-) PDL1(Lo/-) Ly6C(++)
CD8-T Root:		CD3(+) CD11b(-) CD19(-) CD4(-) CD8(+)
**FS50-41-1**	$\gamma\delta$-T Like, CM	PDL1(+) Ly6C(+++) CD49b(Lo/-)
**FS50-34**	$\gamma\delta$-T Like, EM	PDL1(++) CD49b(+) Ly6C(+++)
**FS50-43-1**	$\gamma\delta$-T Like, EM	PDL1(+) Ly6C(+++)
**FS50-27-2**	$\gamma\delta$-T Like, EM	PDL1(+) PD1(-) CD49b(+)
**FS50-33**	$\gamma\delta$-T Like, EM	PDL1(+) PD1(+++) CD49b(+++) Ly6C(Lo/-)
**FS50-06-1**	$\gamma\delta$-T Like, Naï	PDL1(+++) PD1(+++)
$\gamma\delta$-T Like Root:		CD3(+) CD11b(-) CD19(-) CD4(-) CD8(-)
**FS50-46-2**	NK, CM	PDL1(+) CD49b(+++) CD11b(+)
**FS50-49**	NK, CM	PDL1(+) CD49b(+++) NK1.1(+++) CD11b(+) Ly6C(+)
**FS50-46-1**	NK, EM	PDL1(+) CD49b(++) CD11c(Lo/-)
NK Root:		CD11b(-/Lo) CD19(-) NK1.1(+) CD49b(+)
**FS50-38-3**	NK, NK-T, CM	CD3(+) PDL1(++) CD49b(++) NK1.1(++) Ly6C(+++) CD11c(-)

**FS50-16-3**	NK, NK-T, EM	CD3(++) CD4(-) PDL1(+) PD1(-) Ly6C(+) NK1.1(++)
**FS50-16-1**	NK, NK-T, EM	CD3(++) CD4(++) PDL1(+) PD1(-) Ly6C(+) NK1.1(++)
	NK-T Root:	CD3(+) CD11b(-/Lo) CD19(-) NK1.1(+) CD49b(+)
**FS50-30**	B Cell	CD44(+)
**FS50-25**	B Cell	CD62L(-) CD44(-) PDL1(+)
**FS50-22**	B Cell	CD62L(-) CD44(+)
**FS50-26**	B Cell	CD62L(-) CD44(+) PDL1(-)
**FS50-18**	B Cell	CD62L(+) CD44(+) PDL1(-) PD1(-) Ly6C(-)
**FS50-13**	B Cell	CD62L(+) CD44(++) PDL1(+) PD1(-) Ly6C(-)
**FS50-19-1**	B Cell	CD62L(+) CD44(++) PDL1(+++) Ly6C(-) CD11b(+/Lo)
**FS50-10**	B Cell	CD62L(++) CD44(++) PDL1(-) PD1(-) Ly6C(-)
**FS50-11**	B Cell	CD62L(++) CD44(++) PDL1(+) PD1(-/Lo) Ly6C(-) CD206(+)
**FS50-03**	B Cell	CD62L(+++) CD44(++) PDL1(+) PD1(-) Ly6C(-)
**FS50-19-2**	B Cell	CD62L(Lo/-) CD44(+) PDL1(++) Ly6C(+) CD11b(-)
**FS50-29-2**	B Cell	No additional markers
	B Cell Root:	CD3(-) CD11b(-) CD19(+)
**FS50-48**	Neutrophil	CD11b(++) CD44(++) PDL1(-) CD49b(-) Ly6C(++)Ly6G(+)
**FS50-45**	Neutrophil	CD44(++) PDL1(-) CD49b(+)
**FS50-35**	Neutrophil	CD44(++) PDL1(-) CD49b(++) CD206(Lo/-)
**FS50-39**	Neutrophil	CD44(+++) CD49b(++)
**FS50-32**	Neutrophil	CD44(+++) PDL1(++) CD49b(+++)
	Neutrophil Root:	CD3(-) CD11b(+) CD19(-) Ly6C(+) Ly6G(+)
**FS50-21**	Mac, M1	CD11b(++) CD44(+++) PDL1(++) CD49b(+++) Ly6C(+++) CD11c(+) F4/80(+++) CD206(-)
**FS50-17-2**	Mac, M1	CD11b(++) CD44(+++) PDL1(+++) PD1(-) CD49b(+) Ly6C(+) CD11c(+) F4/80(++) CD206(-)
**FS50-28-1**	Mac, M1	CD44(+++) Ly6C(+) CD11c(-/Lo) F4/80(++)
	M1 Macrophage Root:	CD3(-) CD11b(+) CD19(-) F4/80(+) CD206(-)
**FS50-37-1**	Mac, M2	CD11b(+) CD44(++) CD49b(-) Ly6C(-) F4/80(Lo/-) CD206(++)
**FS50-36**	Mac, M2	CD11b(+) CD44(++) PDL1(+) CD11c(+) F4/80(+) CD206(+)
**FS50-02**	Mac, M2	CD11b(++) CD44(++) PDL1(+++) PD1(-) Ly6C(-) CD11c(Lo/-) F4/80(++) CD206(+++)
**FS50-09**	Mac, M2	CD11b(++) CD44(+++) PDL1(+++) CD49b(+) Ly6C(-) CD11c(++) F4/80(+) CD206(++)
**FS50-17-1**	Mac, M2:	CD11b(++) CD44(+++) PDL1(+++) PD1(-) CD49b(+++) Ly6C(+) CD11c(+++) F4/80(Lo/-) CD206(++)
**FS50-01**	Mac, M2	CD11b(+++) CD44(+++) PDL1(++) PD1(-) Ly6C(-) CD11c(++) F4/80(++) CD206(++)
**FS50-12-2**	Mac, M2	CD11b(+++) CD44(+++) PDL1(++) PD1(-) Ly6C(+++) CD49b(+) CD11c(++) F4/80(++) CD206(+++)
**FS50-12-1**	Mac, M2	CD11b(+++) CD44(+++) PDL1(+++) PD1(-) Ly6C(+++) CD49b(++) CD11c(++) F4/80(+++) CD206(++)
**FS50-37-2**	Mac, M2	CD11b(Lo/-) CD44(+) PDL1(+) PD1(Lo/-) Ly6C(+) NK1.1(+++) CD49b(-) CD11c(-) F4/80(-) CD206(++)
	M2 Macrophage Root:	CD3(-) CD11b(+) CD19(-) F4/80(+) CD206(+)
**FS50-40**	DC, cDC:	CD11b(-/Lo) CD44(++) PDL1(+++) CD49b(-) Ly6C(-) CD11c(++) F4/80(-) CD206(Lo/-)
**FS50-28-2**	DC, cDC:	CD11b(-/Lo) CD44(+++) PDL1(+++) CD49b(-/+) Ly6C(-/Lo) CD11c(+++) F4/80(-)
	cDC Root:	CD3(-) CD11b(Lo/-) CD19(-) CD11c(+) Ly6C(Lo/+)
**FS50-38-2**	DC, pDC:	CD62L(-) CD44(++) PDL1(+) Ly6C(+++) CD49b(++) NK1.1(-) CD11b(-/Lo) CD11c(++)
	pDC Root:	CD3(-) CD11b(Lo/-) CD19(-) CD11c(Lo/+) Ly6C(+)

**FS50-06-2**	Artifact	(Residual Immature Thymocytes)
**FS50-29-1**	Artifact	(Residual Immature Thymocytes)
**FS50-16-2**	Artifact	Flow Artifact

**FS50-47**	Unclassified	CD19(Lo/-) CD3(Lo/-) CD11b(-) CD44(Lo/-) Ly6C(Lo/-) NK1.1(Lo/+) CD49b(-) CD206(Lo/-)
**FS50-37-3**	Unclassified*	CD11b(+) CD44(++) PDL1(+++) CD49b(+++) NK1.1(-) Ly6C(-) CD11c(-) F4/80(-) CD206(-)
**FS50-05-1**	Unclassified	Unclassified: All (-)
**FS50-05-2**	Unclassified	All(-) Except CD62L(++)
**FS50-27-1**	Unclassified	CD44(+++) PD1(+++) Ly6C(+) CD49b(+++)

*Potentially Immature Myeloid

Table S4: List of Extracellular flow cytometry Panel with 38 fluorophores, related to Figure 4, Figure S13, S23 – S24

Antibody	Conjugate	Clone	Source	Identifier
CD45.1	BUV395	A20	BD	565212
CD24	BUV496	M1/69	BD	612953
CD44	BUV563	IM7	BD	569705
CD11c	BUV615	HL3	BD	751265
CD11b	BUV661	M1/70	BD	565080
CD8a	BUV737	53-6.7	BD	612759
CD103	BUV805	M290	BD	741948
CD163	BV421	S15049I	BioLegend	155309
CD62L	eFluor 450	MEL-14	Thermo	48-0621-82
CD3	BV480	500A2	BD	746776
CD80	BV510	16-10A1	BioLegend	104741
Ly6C	BV570	HK1.4	BioLegend	128030
CD86	BV605	GL1	BioLegend	105037
NK1.1	BV650	PK136	BD	564143
CD206	BV711	C068C2	BioLegend	141727
CD90.1	BV750	OX-7	BD	746961
XCR1	BV785	ZET	BioLegend	148225
CD19	SparkBlue550	6D5	BioLegend	115566
CD4	SparkBlue574	GK1.5	BioLegend	100490
Ly6G	PerCP	1A8	BioLegend	127654
CD279 (PD-1)	BB700	J43	BD	566514
CD25	PerCP-eFluor 710	PC61.5	Thermo	46-0251-82
CD274 (PD-L1)	PE	10F.9G2	BioLegend	124308
F4/80	Spark YG 593	QA17A29	BioLegend	157312
CD276 (B7-H3)	PE-Dazzle594	MIH35	BioLegend	135612
CD127	PE-Cy5	A7R34	BioLegend	135016
CD49b	PE-Cy5.5	DX5	Thermo	35-5971-82
CD366 (TIM3)	PE-Cy7	RMT3-23	BioLegend	119716
MHC-Class II	PE-Fire810	M5/114.15.2	BioLegend	107667
CD209a	APC	5H10	BD	564928
CD64a/b	AlexaFluor647	X54-5/7.1	BD	558539
CD69	SparkNIR685	H1.2F3	BioLegend	104558
CD152 (CTLA-4)	APC-R700	UC10-4F10-11	BD	565778
CD223 (LAG3)	APC-Fire750	C9B7W	BioLegend	125240
CD45	APC-Fire810	30-F11	BioLegend	103174
**Other Fluorophores Present:**				
Live/Dead Fixable Blue	N/A	N/A	Thermo	L23105
GFP				
Ametrine				

38 Total Chanels

Table S6: List of Intracellular flow cytometry Panel with 25 fluorophores, related to Figure 4 and Figure S26

Antibody	Conjugate	Clone	Source	Identifier
IL-17	BUV395	TC11-18H10	BD	565246
CD11c	BUV615	HL3	BD	751265
CD11b	BUV661	M1/70	BD	565080
IFNy	BUV737	XMG1.2	BD	612769
CD4	BUV805	GK1.5	BD	564922
RORgt	BV421	Q31-378	BD	562894
CD3	BV480	500A2	BD	746776
CD25	BV510	PC61	Biol	102042
Ly6C	BV570	HK1.4	BioLegend	128030
TCRb	BV605	H57-597	BD	562840
TNFa	BV650	MP6-XT22	BD	563943
CD8a	BV711	53-6.7	BD	563046
CD45.1	BV786	A20	BD	740889
Ly6G	PerCP	1A8	BioLegend	127654
ARG1	PerCP-eFluor 710	A1exF5	Thermo	46-3697-82
IL-6	PE	MP5-20F3	BioL	504504
F4/80	Spark YG 593	QA17A29	BioLegend	157312
TCF1	Alexa594	C63D9 (??)	CST	35972S
IL-10	PE-Cy7	JES5-16E3	BioLegend	505026
Foxp3	APC	FJK-16s	Thermo	17-5773-82
B220	Alexa Fluor 700	RA3-6B2	Thermo	56-0452-82
CD45	APC-Fire 810	30-F11	BioLegend	103174
BATF	Alexa-Fluor 647	D7C5	CST	47914S
**Other Fluorophores Present:**				
Live/Dead Fixable Blue	N/A	N/A	Thermo	L23105
GFP				
Ametrine				

Comment: FoxP3 was replaced with BATF in the experiment where N=4

25 Total Chanels

Table S7: List of Extracellular flow cytometry Panel for Human CAR T cell analysis, related to Figure 7, Figure S31 and Figure S33

Antibody or Chimeric protein	Conjugate	Clone	Source	Identifier
B7-H3 chimeric protein	PE	-	Acro Biosystems	B7B-HP2H9-25tests
CD4	BV785	SK3	Biolegend	344642
CD8	BV510	SK1	BD	563919
CD45RO	BV650	UCHL1	Biolegend	304232
CCR7	FITC	REA546 | G043H7	Miltenyi Biotec	130-120-468
CD39	BV605	A1	Biolegend	328236
PD1	PerCP5.5	EH12.2H7	Biolegend	329920
TIM3	PE-Cy7	F38-2E2	Biolegend	345014
**Isotype control**	**Conjugate**	**Clone**	**Source**	**Identifier**
IgG1	BV785	MOPC-21	Biolegend	400170
IgG1	BV510	MOPC-21	Biolegend	400172
IgG2a	BV650	MOPC-173	Biolegend	400266
IgG1	FITC	REA293 | IS5-21F5	Miltenyi Biotec	130-113-437
IgG1	BV605	MOPC-21	Biolegend	400162
IgG1	PerCP5.5	MOPC-21	Biolegend	400150
IgG1	PE-Cy7	MOPC-21	Biolegend	400126
Dapi	PB450	-	BD	564907

Table S8: Antibodies and Methods used for Immunohistochemistry, related to Figure S2, Figure S5, Figure S6, Figure S10 and Figure S25

Antibody	Species	Clone	Dilution	Catolog number	Supplier
CD276 (B7-H3)	Rabbit monoclonal	EPR27429-173	1:100	ab313380[a]	Abcam
ARGINASE 1	Rabbit monoclonal	D4E2M	1:75	93668[b]	Cell Signaling
CD68	Rat Ig2a	FA-11	1:1000	MCA1957[a]	BioRad
CD163	Rabbit monoclonal	EPR19518	1:1200	ab182422[a]	Abcam

a Heat-induced epitope retrieval, Epitope Retrieval solution 2 (ER2), 20 minutes; Visualization with rabbit anti-goat (BA-5000; Vector Laboratories), Bond Polymer Refine Detection (DS9800, Leica Biosystems)

b Heat-induced epitope retrieval, Cell conditioning media 2 (Ventana Medical Systems, Tucson, AZ), 32 minutes; Visualization with DISCOVERY OmniMap anti-Rb HRP (760-4311; Ventana Medical Systems), DISCOVERY ChromoMap DAB kit (760-159; Ventana Medical Systems) or DISCOVERY ChromoMap Purple kit (760-229; Ventana Medical Systems)
